# Length of course-based undergraduate research experiences (CURE) impacts student learning and attitudinal outcomes: A study of the Malate dehydrogenase CUREs Community (MCC)

**DOI:** 10.1371/journal.pone.0282170

**Published:** 2023-03-09

**Authors:** Sue Ellen DeChenne-Peters, John F. Rakus, Amy D. Parente, Tamara L. Mans, Rebecca Eddy, Nicole Galport, Courtney Koletar, Joseph J. Provost, J. Ellis Bell, Jessica K. Bell

**Affiliations:** 1 Department of Biology, Georgia Southern University, Savannah, Georgia, United States of America; 2 Department of Chemistry, Marshall University, Huntington, West Virginia, United States of America; 3 Department of Chemistry and Biochemistry, Mercyhurst University, Erie, Pennsylvania, United States of America; 4 Department of Biology, North Hennepin Community College, Brooklyn Park, Minnesota, United States of America; 5 Cobblestone Evaluation and Applied Research, Inc., La Verne, California, United States of America; 6 Department of Chemistry and Biochemistry, University of San Diego, San Diego, California, United States of America; Touro University, UNITED STATES

## Abstract

Course-based undergraduate research experiences (CUREs) are laboratory courses that integrate broadly relevant problems, discovery, use of the scientific process, collaboration, and iteration to provide more students with research experiences than is possible in individually mentored faculty laboratories. Members of the national Malate dehydrogenase CUREs Community (MCC) investigated the differences in student impacts between traditional laboratory courses (control), a short module CURE within traditional laboratory courses (mCURE), and CUREs lasting the entire course (cCURE). The sample included approximately 1,500 students taught by 22 faculty at 19 institutions. We investigated course structures for elements of a CURE and student outcomes including student knowledge, student learning, student attitudes, interest in future research, overall experience, future GPA, and retention in STEM. We also disaggregated the data to investigate whether underrepresented minority (URM) outcomes were different from White and Asian students. We found that the less time students spent in the CURE the less the course was reported to contain experiences indicative of a CURE. The cCURE imparted the largest impacts for experimental design, career interests, and plans to conduct future research, while the remaining outcomes were similar between the three conditions. The mCURE student outcomes were similar to control courses for most outcomes measured in this study. However, for experimental design, the mCURE was not significantly different than either the control or cCURE. Comparing URM and White/Asian student outcomes indicated no difference for condition, except for interest in future research. Notably, the URM students in the mCURE condition had significantly higher interest in conducting research in the future than White/Asian students.

## Introduction

Careers in Science, Technology, Engineering, and Mathematics (STEM) fields are growing at a faster rate than non-STEM careers and provide higher salaries than the United States national average salary [1]. While interest in STEM careers among incoming college freshmen is high, only 50% to 60% of those students will graduate with a degree in a STEM major [2–4]. There is ample evidence that many of these students leave STEM for another major despite being highly qualified in indicators suggestive of STEM success [4]. Attrition rates are even higher for students whose ethnicity/race is traditionally under-represented in STEM fields (non-White/Asian groups) despite more under-represented minority (URM) students entering college intending to major in STEM fields [5]. Undergraduate research experiences (UREs) are among the several high impact educational practices shown to increase retention of STEM students in STEM [6–8].

Undergraduate research experiences (UREs) enhance students’ scientific processing skills, identity, self-efficacy, and content knowledge. They also increase graduation rates and the likelihood of pursuing post-baccalaureate education [7–11]. Additionally, UREs can have even stronger positive impacts for URM students [12–16]. Unfortunately, due to constraints on faculty time, money, and space, there are not enough URE opportunities for all students who wish to participate [17, 18]. Furthermore, students may not be aware that UREs are available, how to obtain one, or may lack the time outside of classes to participate [19]. These limitations on UREs may disproportionately impact URM students [18, 19]. Because course-based undergraduate research experiences (CUREs) can be implemented in normally scheduled classes, they lower most of these barriers to URE access [19, 20] while providing many of the same benefits [21].

CUREs are a natural extension of teaching (for faculty) and experiencing (for students) the scientific method in the curriculum. Distinct from standard “cookbook” or inquiry-based labs, CUREs incorporate five dimensions: broadly relevant problems, discovery, use of scientific processing skills, collaboration, and iteration [21]. CUREs have become widespread in multiple scientific disciplines, have been adopted in introductory to advanced courses, can be implemented for part of, or an entire course, and in small classes taught by a single faculty member to scaled up versions in large laboratory sections taught by graduate teaching assistants [e.g. 22, 23]. CUREs have multiple benefits including: student learning [24, 25], analytical skills [26, 27], attitudinal outcomes [22, 28–30], self-reported gains in research skills [31–34], and persistence in science [35].

While there are many benefits to involvement with UREs and CUREs, and faculty have implemented part and full-semester CURES, there is little research on the impacts of URE/CURE duration. There is some evidence that multi-semester UREs increase student GPA [36], probability of earning a doctoral degree [12], and various skills needed to be a research scientist [34]. Taking multiple CUREs increases graduation rates [37] and intent to become a research scientist [38]. These studies indicate it is probable that multiple semesters of UREs/CUREs improve student outcomes compared to single semester CUREs. However, development of single or multiple semester CUREs can be logistically difficult and costly, especially in already established and/or large enrollment courses [39, 40]. To reduce these challenges, CUREs can also be taught in modules within a full semester laboratory course [e.g. 41]. Preliminary evidence indicates that there are positive impacts for engaging in shorter modular CUREs (mCUREs) including increased interest in graduate school [42], improved project ownership [43], and increased problem solving [44]. While mCUREs can show some benefits, the limited studies comparing CURE duration indicate longer time increases benefits [27, 43, 45, 46]. Most of these studies used either student self-reported learning gains or content knowledge specific to the CURE. Comparing objective tests of student learning across different CURE subject matter is difficult. However, this emphasizes the need for broader objective tests of learning to understand student outcomes based on differential time spent in CUREs.

CUREs provide an equitable means to offer research experiences to all students. Despite calls for research on URM experiences in CUREs [47–49], only a few studies have examined possible differential student outcomes for URM students in CUREs. Most of these studies indicate that students, regardless of demographic status, experience CURE gains similarly [35, 37, 50, 51]. Several others have indicated an increased interest for graduate school [42], higher levels of project ownership [35], and improved self-efficacy [30] for URM students in CUREs. While these studies indicate that CUREs can be a vehicle to improve URM student outcomes, more research is needed to understand the impacts of CUREs on diverse student populations. Additionally, there are no studies that examine the impacts of CURE duration on URM student outcomes.

Several highly successful, long-running biochemistry and molecular biology CUREs have been established. The Genomics Education Partnership (GEP) currently includes more than 100 partner institutions which share resources, training, and mentorship in developing genomics- and bioinformatics-based CUREs [52]. The Science Education Alliance-Phage Hunters Advancing Genomes and Evolutionary Science (SEA-PHAGES) coalition began in 2014 and has expanded to include nearly 200 institutions including community colleges, research intensive universities, and primarily undergraduate institutions [53]. In 2016, we and others noted a general lack of protein-focused CURE projects and called on the community to address this need [54, 55]. The following year, we established the Malate dehydrogenase CUREs Community (MCC) with the goal of developing a robust, self-sustaining coalition of faculty implementing protein-focused CUREs using the Citric Acid Cycle enzyme malate dehydrogenase (MDH) as the shared model system. As of October 2021, the MCC has grown to include one or more faculty members from at least 25 U.S. institutions.

**Fig 1:**
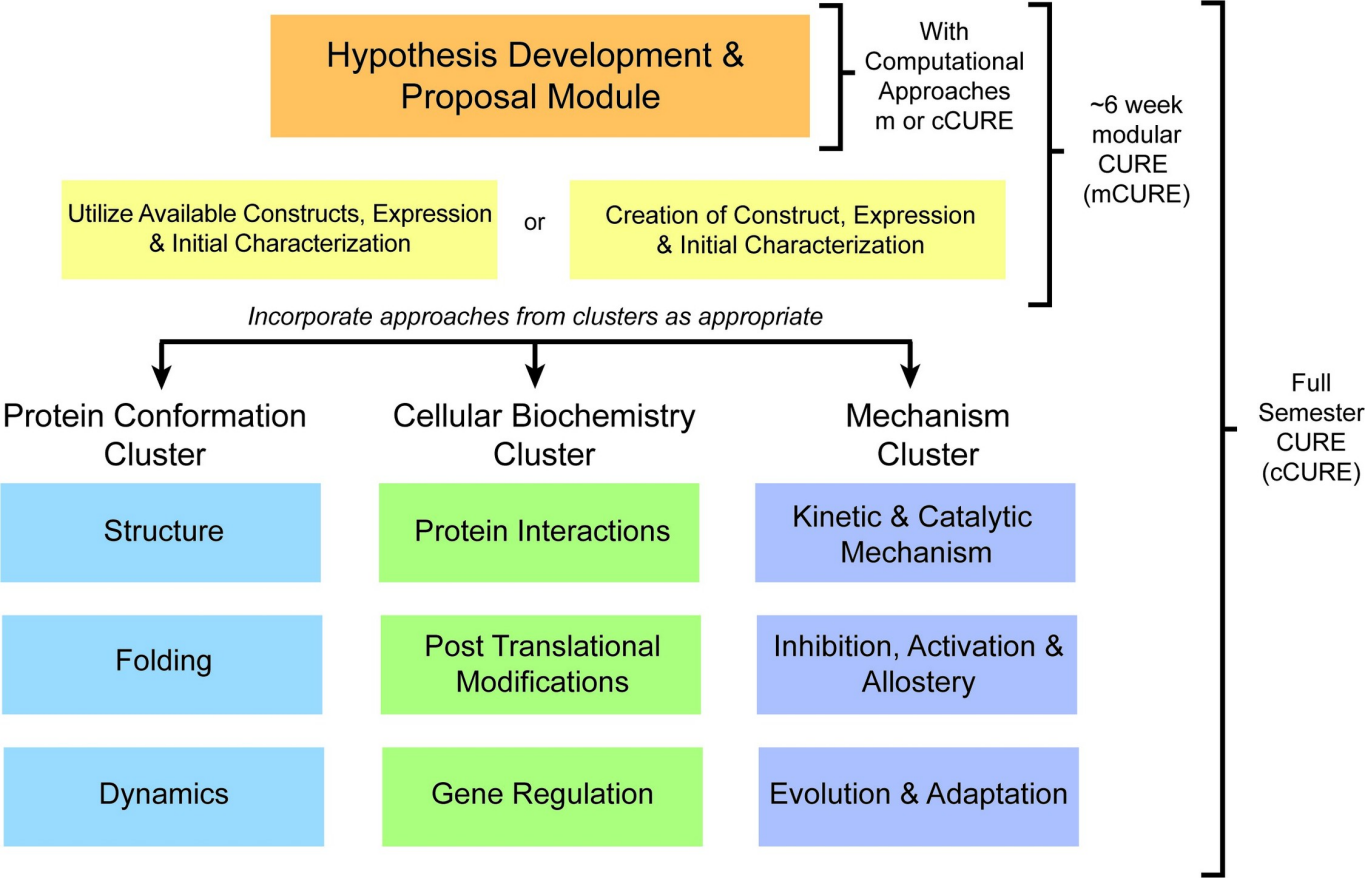
The Malate dehydrogenase CUREs Community (MCC) model. Faculty can implement a malate dehydrogenase focused CURE for a short module within a laboratory course (mCURE) or a full semester (cCURE). All CUREs start with the hypothesis module. There are three experimental clusters: Protein Conformation, Cellular Biochemistry, and Mechanism.

Members of the MCC have incorporated a variety of MDH-focused projects into their courses. Collectively, the courses include undergraduates in their first through senior year of study in biology, chemistry, and biochemistry, as well as non-science majors. Each MCC CURE incorporates the following features: relevance, literature review, hypothesis development, proposal, experiments, data analysis, drawing conclusions, and presentation of research. Though the specifics of the projects undertaken in each course vary widely, the MCC members have categorized their approaches in three clusters: Protein Conformation, Cellular Biochemistry, and Mechanism. The Protein Conformation Cluster focuses on MDH structure, folding, and dynamics. The Cellular Biochemistry Cluster investigates MDH protein-protein interactions, post-translational modifications, and genetic regulation. The Mechanism Cluster approaches MDH kinetics and the catalytic mechanism, allosteric inhibition and activation, and evolution and adaptation. All MCC projects begin with a Hypothesis Development Module [56]. The mCUREs, which are usually about six weeks, follow hypothesis development with initial characterization of previously generated relevant constructs so students have data to analyze and evaluate their hypotheses (Fig 1). The complete CURE (cCURE) lasts for a full semester and includes initial characterization following hypothesis development, but then provides additional time for students to conduct a more robust investigation which may involve several complementary approaches to interrogate their hypotheses (Fig 1). This model of related, but different, CUREs contrasts with SEA-PHAGES and GEP, which use very closely related CURE curricula. These unique features of the MCC allows the comparison of CURE outcomes across a wide variety of CURE curricula and time spent in CUREs, which is lacking in the current literature [45].

The MCC provides a unique opportunity to explore student outcomes across related, but different, CURE contexts. The institutions within the MCC encompass community colleges, primarily undergraduate institutions, and research institutions; represent both public and private institutions; and are highly diverse in student population demographics. CUREs were taught in lower-level and upper-division courses, for a full or partial semester, and control data were collected in non-CURE laboratory experiences. With almost 1,500 students, 22 faculty, and 19 institutions involved in the MCC educational research component, we explored the following research questions:

1. Is there a difference in laboratory course elements between control laboratory courses, mCUREs, and cCUREs?
2. Is there a difference in laboratory course elements between control laboratory courses, mCUREs, and cCUREs as perceived by URM students?
3. Is there a difference in student outcomes between control laboratory courses, mCUREs, and cCUREs?
4. Is there a differential impact for URM students between control laboratory courses, mCUREs, and cCUREs?

## Materials and methods

### Participating institutions

As part of a larger research study, we recruited 22 biology and chemistry faculty at 19 U.S. institutions. These institutions represented a wide variety of public and private college and university types including: four research intensive universities, 11 primarily undergraduate institutions, and four community colleges. They ranged in size from 1,000–26,000 enrolled undergraduate students. A total of 1,478 students consented to participate in the research: 603 in the control classes, 549 in mCUREs, and 326 in cCUREs (Table 1). Students classified as URM included those traditionally under-represented in STEM disciplines: American Indian/Alaska Native, Black/African American, Hispanic/Latino, Native Hawaiian/Pacific Islander, and two or more races/ethnicities.

**Table 1:** Student demographics.

Demographic	Control	mCURE	cCURE
Female	230 (38.1%)	213 (38.8%)	153 (46.9%)
Male	371 (61.5%)	327 (59.6%)	172 (52.8%)
Unknown Gender	2 (0.4%)	9 (1.6%)	1 (0.3%)
American Indian/Alaska Native	2 (0.3%)	3 (0.5%)	0 (0.0%)
Asian	87 (14.4%)	65 (11.8%)	45 (13.8%)
Black or African American	21 (3.5%)	33 (6.0%)	18 (5.5%)
Hispanic or Latino	98 (16.3%)	114 (20.8%)	22 (6.7%)
Native Hawaiian or Other Pacific Islander	1 (0.2%)	6 (1.1%)	0 (0.0%)
White	331 (54.9%)	252 (45.9%)	205 (62.9%)
Two or more races	23 (3.8%)	24 (4.4%)	16 (4.9%)
Other^a^	16 (2.7%)	36 (6.6%)	12 (3.7%)
Unknown ethnicity	24 (4.0%)	16 (2.9%)	8 (2.4%)
URM^b^	145 (24.0%)	180 (32.8%)	56 (17.2%)
White/Asian	418 (69.3%)	317 (57.7%)	250 (76.7%)

^a^Includes designations from institutional research departments of non-resident alien, international, foreign national, and other.

^b^Includes American Indian/Alaska Native, Black or African American, Hispanic or Latino, Native Hawaiian or Other Pacific Islander, and Two or more races.

All MCC participating institutions received institutional review board (IRB) approval for the research study, either through their university IRB or via IRB authorization agreements with the originating institution, University of San Diego (USD) (Table 2). Participants’ informed consent were collected in written form through an informed consent document.

**Table 2:** IRB agreement numbers and authorizing institutions.

Institution	IRB # or Authorization
Cobblestone Applied Research and Evaluation, Inc.	USD #IRB-201864
Georgia Southern University	USD #IRB-201864
Hamline University	USD #IRB-201864
Hampden-Sydney University	Letter indicating approval by Human Research Committee
Illinois Valley Community College	File # 003–2019
Malone University	Malone U. Protocol KHui-CHEM374#2-CUREs
Marshall University	USD #IRB-201864
Mercyhurst University	MU IRB Reg # IRB00007637, FWA# 00024187
North Hennepin Community College	NHCC IRB #: 190823_Galport
Rensselaer Polytechnic Institute	USD #IRB-201864
San Francisco State University	Protocol Number: X18-63
Southwestern Community College	USD #IRB-201864
St. John Fisher College	File No: 3817-122117-16
Suffolk University	USD #IRB-201864
University Connecticut—Hartford	USD #IRB-201864
University of Massachusetts—Amherst	Letter stating that UMass Amherst HRPO approves request
University of Nebraska—Lincoln	IRB Number: 20171217498 EX; Project ID: 17498
Union College	Email confirmation of exemption from chair
University of New Mexico–Albuquerque	USD #IRB-201864
University of San Diego (USD)	#IRB-2018-64

### Data collection

Data were collected by the external evaluator (Cobblestone Inc., La Verne, CA) over a period of two years in a total of 76 courses/sections with 43 having a CURE component (22 mCURE, 21 cCURE) and the remaining 33 courses serving as non-CURE controls (these courses did not meet all the elements of a CURE) [21]. The control labs varied from standalone verification laboratory exercises to inquiry laboratory exercises. In inquiry laboratory exercises, the instructor knows the outcome, but the students are designing and carrying out the experimental protocol [57]. CURE courses were taught by MCC faculty and control courses were either taught by MCC faculty members or by faculty recruited by MCC members. Not all institutions provided control courses. Data were collected during Spring 2018, Fall 2018, Spring 2019, and Fall 2019 semesters. Due to the COVID-19 pandemic, data collection was interrupted in Spring 2020 and not included in this analysis. Fifteen faculty members began collecting data in the Spring semester of 2018, three more joined data collection in Fall 2018, and four more joined Fall 2019. Evaluation consent forms and surveys were administered to students by the participating MCC faculty members; consent forms and pretest assessments were administered to students on the first day of class and posttest assessments were administered on the last day of class. All evaluation surveys were completed online except for the EDAT, which students completed by pen and paper and were sent to Cobblestone by the faculty.

### Instruments

The following seven instruments were used in this study:

1. The Laboratory Course Assessment Survey (LCAS) is a post-only 17-item survey instrument that assesses students’ perceptions of collaboration, discovery, and iteration in lab courses. The instrument is meant to differentiate between traditional laboratory courses and CUREs. Cronbach’s alpha for each LCAS scale ranges between 0.80–0.85 [58]. Similar results were found with our samples (collaboration, α = 0.81; discovery and relevance, α = 0.91; iteration, α = 0.88) and mean composite scores for each scale were calculated.
2. The Test of Scientific Literacy Skills (TOSLS) is a 28-item, pretest/posttest multiple choice survey that assesses students’ scientific literacy skills such as recognizing and analyzing methods as well as interpreting quantitative data [59]. The percentage of questions that students answered correctly was calculated for both pretest and posttest.
3. The Experimental Design Ability Test (EDAT) is a pretest/posttest that asks students to describe how they would design an experiment to a provided scenario. Student responses are assessed using a standard rubric [60]. A total of 1,187 students completed the EDAT both prior to and after completing their course. Each EDAT was scored by two faculty members from institutions other than the students’ own. Faculty members indicated the presence or absence of ten elements of experimental design, resulting in scores ranging from zero to ten. Some completed EDAT posttests in Year 3 were not rated by any faculty members, reducing the total matched pretest and posttest number to 1,139. Scores from three raters who had an interrater reliability of less than 0.500 on the posttest were removed, resulting in 1,044 matched student pretest and posttest scores available for analysis. For EDAT responses with two usable ratings, the student’s score was represented by the mean of the two ratings. For EDAT responses with only one usable rating, the single rater’s rating was used as the student’s score. For the 862 pretest responses with valid scores from two faculty members, there was an interrater reliability of *r* = 0.704 (*p* < 0.001). For the 629 posttest responses with valid scores from two faculty members, there was an interrater reliability of *r* = 0.666 (*p* < 0.001). These correlations are below those reported by Sirum and Humburg in the development of the EDAT (*r* = 0.835, *p* < 0.001).
4. The Student CURE survey is a pretest/posttest survey that assesses students’ experiences in CUREs. Topics assessed include: (a) course elements, (b) overall evaluation, (c) learning gains, (d) positive/negative attitudes toward science, and (e) beliefs about science/science learning [61].
  - Course Elements: At pretest, students rated their experience with 25 learning activities that occur in science courses using a scale from 1 = “No experience or feel inexperienced” to 5 = “Extensive experience or mastered this element.” At the posttest, students rated their learning gains related to each of the 25 activities using the scale 1 = “No gain or very small gain” to 5 = “Very large gain.” Items were analyzed individually.
  - Overall Evaluation: On the posttest, students responded to four items evaluating the course overall using a scale from 1 = “Strongly disagree” to 7 = “Strongly agree.” Items were analyzed individually.
  - Learning Gains: On the posttest, students indicated the extent to which they gained 21 different benefits from participating in their course on a scale from 1 = “No gain or very small gain” to 5 = “Very large gain.” The items were used individually and as a composite score. The items were found to be internally consistent (F061 = 0.97) and a composite score was calculated by using the mean of all 21 items.
  - Positive/Negative Attitudes Towards Science: On the pretest, students responded to five items concerning positive attitudes towards science using a scale from 1 = “Strongly disagree” to 7 = “Strongly agree.” On the posttest, students responded to the same five items using a scale from 1 = “Strongly disagree” to 5 = “Strongly agree.” The inter-item correlations were acceptable at both pre- (F061 = 0.71) and posttest (F061 = 0.75) and mean composite scores were calculated for each. On the pretest, students responded to six items concerning negative attitudes towards science using a scale from 1 = “Strongly disagree” to 7 = “Strongly agree.” On the posttest, students responded to the same six items using a scale from 1 = “Strongly disagree” to 5 = “Strongly agree.” The inter-item correlations were low at pretest (F061 = 0.65) and acceptable at posttest (F061 = 0.80). The use of different scales pre- and posttest was due to a data entry error in the survey. Mean composite scores were calculated for each.
  - Beliefs about Science/Science Learning: On the pretest, students responded to 11 items concerning their beliefs using a scale from 1 = “Strongly disagree” to 7 = “Strongly agree.” On the posttest, students responded to the same 11 items using a scale from 1 = “Strongly disagree” to 5 = “Strongly agree.” Items were analyzed individually across the 11 statements.
5. The Student Posttest Survey was created by Cobblestone Evaluation and Applied Research, Inc. specifically for this study. The posttest asks students about (a) previous research experiences and (b) includes the STEM Career Interest Scale. The reported Cronbach’s alpha for this scale was 0.94 [62].
  - Three yes/no questions were asked about prior research experience inside and outside of class and interest in future research. Each item was assessed independently.
  - For STEM support, STEM career interest, and STEM importance at posttest, students responded to four items regarding support, four items regarding career interest, and four items on importance on a scale from 1 = “Strongly disagree” to 5 = “Strongly agree.” The inter-item correlations were high (support F061 = 0.83, career interest F061 = 0.89, importance F061 = 0.85) and a mean composite was calculated for each scale.
6. The Institutional Data Request, developed by Cobblestone, included nine demographic and academic variables: sex, ethnicity, Pell grant eligibility, educational level, major, department, enrollment, cumulative GPA, MCC course grade, and graduation information. Not all institutions responded to this request for information, lowering the sample size for these analyses.
  - One-year post-CURE cumulative GPA was examined for students who participated in a CURE in Winter/Spring 2018 (GPA in Spring 2019), Fall 2018 (GPA in Spring 2019), or Spring 2019 (GPA in Spring 2020). GPA was only examined for students enrolled one-year post CURE (this excludes students who graduated).
  - One-year post-CURE retention was examined for students who participated in a CURE in Winter/Spring 2018 (enrollment/grad status in Spring 2019), Fall 2018 (enrollment/grad status in Spring 2019), or Spring 2019 (enrollment/grad status in Spring 2020). Students who had graduated by the term of interest were counted as retained.
7. A post semester faculty survey that assessed the implementation of CURE elements in the courses to compare to (a) student results from the CURE survey [61] and (b) the grant definition of elements of a CURE.
  - Faculty rated their experience with 25 activities that occur in science courses using 0 “not applicable” to 3 “major”.
  - MCC faculty members reported on the extent to which they implemented CURE-related elements into their course. These elements include the three described by the LCAS (discovery, iteration, and collaboration) as well as those identified in the NSF grant proposal (scientific background, hypothesis development, proposal creation, experiments/teamwork to test hypothesis, data analysis and conclusions, and presentation). The percentage of faculty who reported engaging in these practices in their course was calculated for each category.

### Data analysis

All data analysis was completed by Cobblestone. To compare conditions, all pretest/posttest measures (TOSLS, EDAT, Course Elements, Positive/Negative Attitudes about Science, and Beliefs about Science/Science Learning) were analyzed using analysis of covariance (ANCOVA) with the pretest score as the covariate. Analysis of variance (ANOVA) was run for all posttest-only measures (LCAS, Overall Evaluation, Learning Gains, STEM Career Interest, and GPA). When ANCOVAs or ANOVAs were statistically significant, Tukey HSD post-hoc tests or simple contrasts were run to determine which group(s) was/were significantly different. For ANOVA and ANCOVA tests, the skewness statistic was examined to test distributions for normality. A skewness ≤ |0.50| was normally distributed. A skewness > |0.50| and < |1.00| was considered moderately non-normal. A skewness ≥ |1.00| was considered highly non-normal. Homogeneity of variances was assessed by a Levene’s test for equality of variances. If the Levene’s test was not significant, homogeneity of variances was assumed. For ANCOVA tests, homogeneity of regression slopes was tested by regressing the following on the dependent variable: each level of the independent variable, the covariate, and an interaction between each level of the independent variable and the covariate. If the interaction terms were not significant, homogeneity of regression slopes was assumed. In some cases, one or two assumptions of the parametric tests were violated. ANOVA and ANCOVA are robust to violations of assumptions when they are slight to moderate or when other assumptions are met [63].

Pearson chi-squared analyses were run for dichotomous variables (retention and plans to conduct research). Significance for statistical tests was set at *p* < 0.05. For tests with multiple individually analyzed items, the *p*-value was corrected for family-wise errors (the likelihood of reporting a false positive when doing multiple hypothesis tests) using a Bonferroni correction (*p* < 0.05/(# of tests)). All outliers were retained in the analysis. Missing data was excluded pairwise. All statistical tests were run in SPSS Ver. 27.

## Results

For readability, all test values and effect sizes are in the corresponding supplemental table, only the *p*-values are reported in the text. Students and faculty reported their experiences with various course elements in their classes. The LCAS was used to report student experiences and a faculty survey of ten CURE elements was used to report the use of CURE elements in their classes. We also compared students’ reported learning activities with a faculty reported inventory of course elements using the CURE survey [61], which has 25 laboratory classroom activities.

### Student course elements

**Fig 2:**
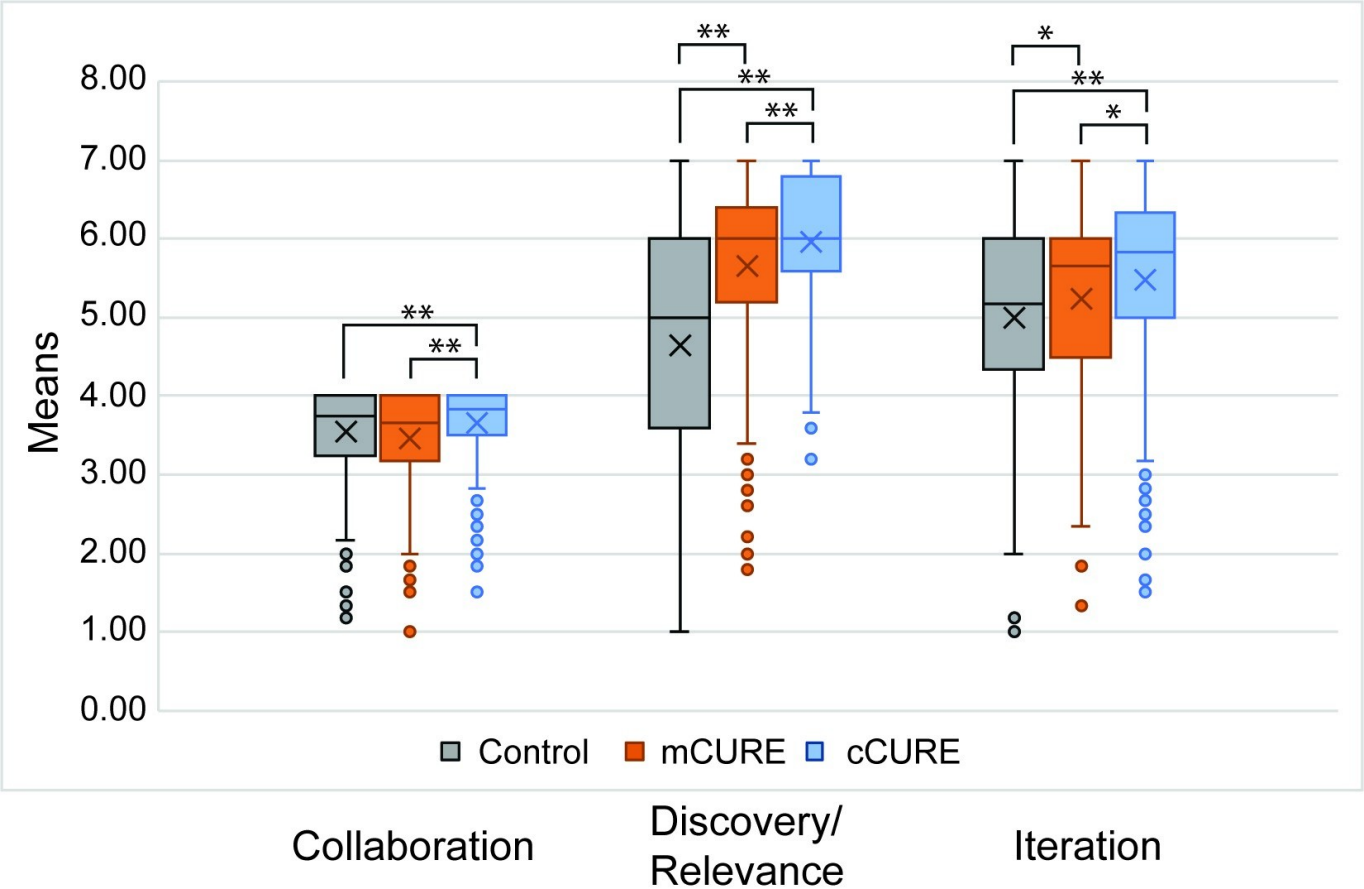
Students experience more CURE elements in cCUREs than mCUREs or control laboratories. Students reported their experiences with three elements of a CURE at the end of the semester. Collaboration was scored using 1 (Never), 2 (One or two times), 3 (Monthly), and 4 (Weekly). Iteration and discovery/relevance elements were scored 1 (Strongly Disagree) to 7 (Strongly Agree). An ANOVA indicates that iteration and discovery/relevance are significantly different (cCURE > mCURE > control). For collaboration, cCURE is significantly higher than mCURE and control. See S1 Table for complete results. ** *p <* 0.0001, * *p* < 0.05.

One-way ANOVAs comparing the three conditions (control, mCURE, and cCURE) were significant for each course element (collaboration: *p* < 0.001; discovery/relevance: *p* < 0.001; iteration: *p* < 0.001) (Fig 2 and S1A Table). For the discovery/relevance and iteration subscales, all three conditions were significantly different from each other based on the Tukey HSD post-hoc test (*p* < 0.05). For collaboration, cCUREs were higher than mCUREs and control courses (*p* < 0.001). There were no differences in collaboration between mCUREs and control courses (*p* > 0.05).

We ran two (URM vs. White/Asian) by three (cCURE, mCURE, control) condition ANOVAs for each course element on the LCAS. There was no significant main effect of URM status and no significant interaction between URM status and condition for the collaboration and discovery/relevance subscales of the LCAS. There was a significant main effect for URM status on the iteration subscale (*p* = 0.005), indicating that URM students, regardless of condition, reported more iteration. However, there was not a significant effect for the interaction between URM status and condition, indicating that the amount of iteration reported by URM students in each condition did not differ. See S1B Table for complete results.

### Faculty course elements

**Fig 3:**
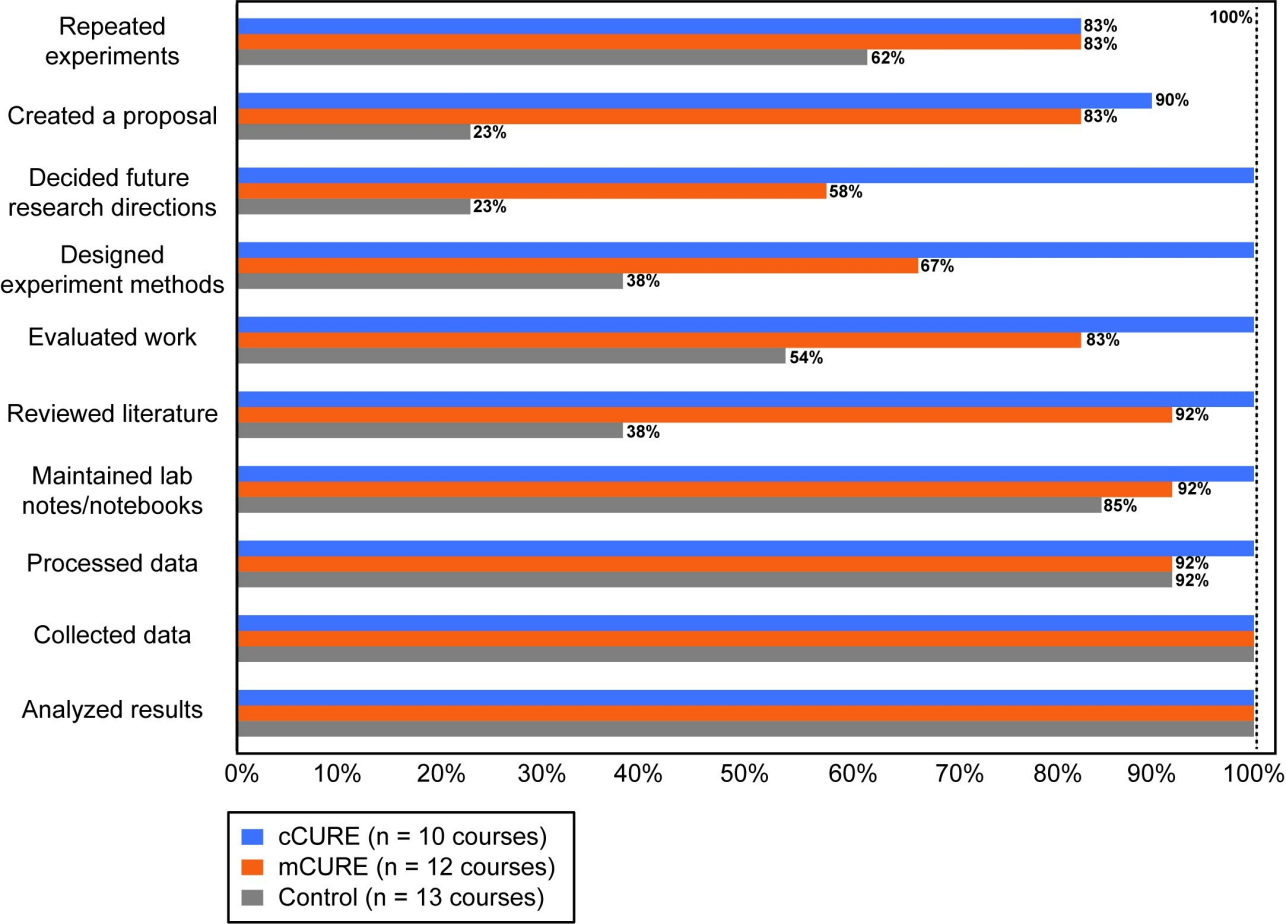
Faculty descriptions of their course elements are consistent with CURE and control classes. The ten elements of a CURE are listed with the percentage of faculty that indicated each element was included in their course. Due to the small sample size, statistical analysis was not done. Descriptively, most cCURE faculty included all 10 elements, most mCURE faculty included 8 of the 10 elements, and most control faculty included 4 of the 10 elements.

Since the number of faculty surveyed was small, faculty data were not compared statistically. Most faculty members indicated that students collected data, processed data, analyzed results, and maintained laboratory notebooks regardless of whether they taught control or CURE courses (Fig 3). The biggest descriptive differences between control and CURE faculty were in reviewing literature, evaluating work, creating proposals, and repeating experiments, with CURE faculty reporting more of these elements in their courses. Two elements, designing experiments and deciding future research directions, appeared descriptively different between all three conditions. Instructors of cCUREs reported more of these elements than mCURE instructors, who reported more than control faculty.

### Comparison of student and faculty learning activities (CURE survey)

**Fig 4:**
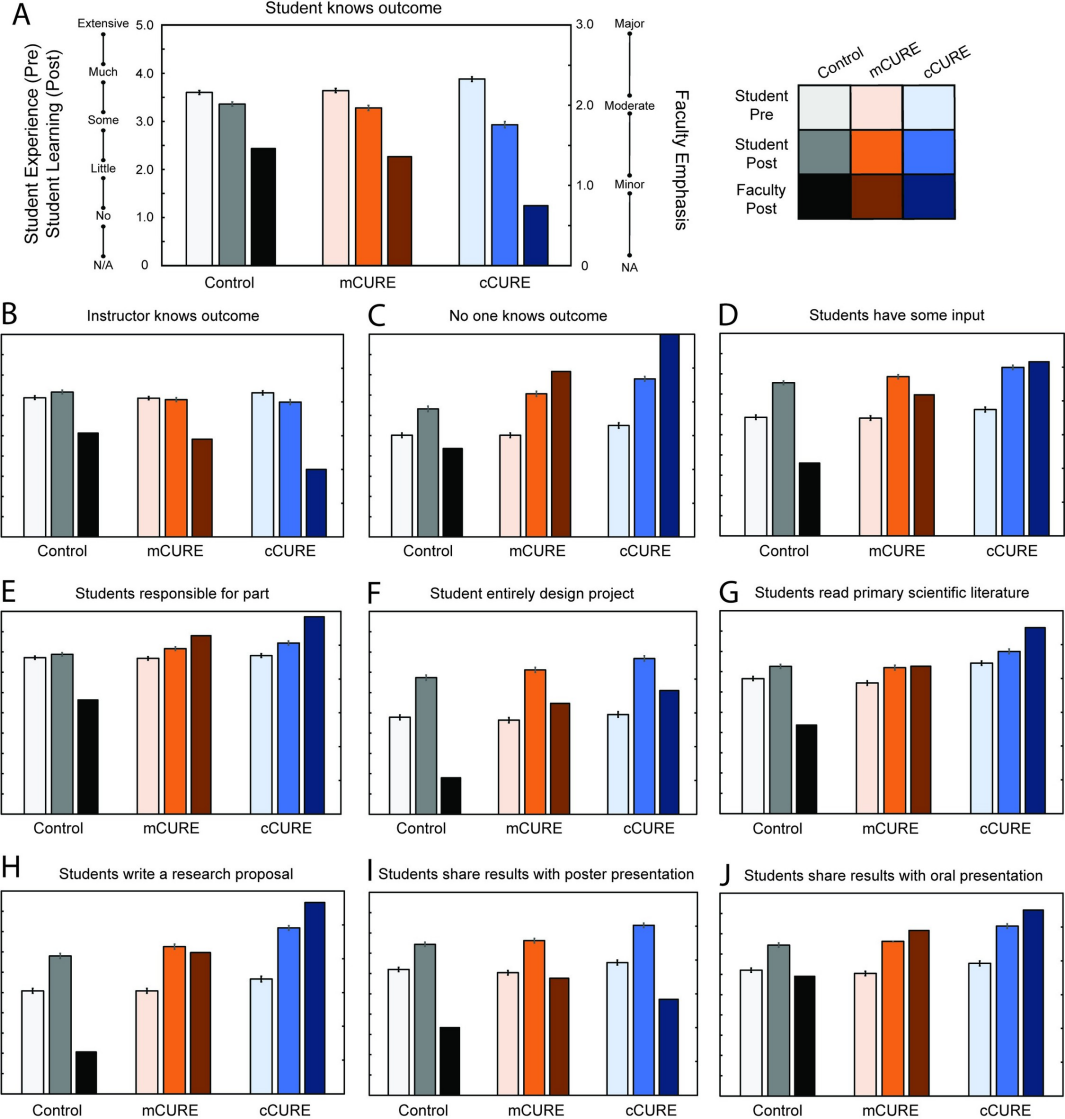
Faculty and student perception of learning activities in courses is consistent with expected CURE outcomes. Students and faculty scored the same 25 learning activities in their classes. On the pretest, students scored each activity on their level of experience with that activity prior to the course (1 = no experience or feel inexperienced to 5 = extensive experience or mastered this element). On the posttest, students scored each activity on how much learning they gained from the activity in their class (1 = no gain or very small gain to 5 = very large gain). After the course, faculty scored how much emphasis they placed on the learning activity on a scale of 0 (not applicable) to 3 (major). An ANCOVA controlling for pretest scores and incorporating a Bonferroni correction for 25 tests (*p* < 0.002) indicated ten learning activities that were significantly different between conditions as reported by students. These ten learning activities are from three groups: 1) knowledge of experimental outcomes (4A – 4C), student involvement in research projects (4D – 4H), and presenting results (4I & 4J). Panel 4A shows detailed axes labels that are used in Panels 4B – 4J. Color coding is as follows: gray to black hue is the control group, peach to dark orange is the mCURE group, and light blue to dark blue is the cCURE group. The lightest hue is the student pre-assessment. The medium hue is the student post-assessment. The darkest hue is the faculty post-assessment. The specific topics in each category are: *Knowledge of experimental outcomes*—(A) lab/project where student knows outcome (mCURE & control > cCURE), (B) lab/project where instructor knows outcome (control > cCURE & mCURE), (C) lab/project where no one knows outcome (cCURE > mCURE > control); *Student involvement in research projects*—(D) student input into research (cCURE > mCURE & control, (E) responsible for part of the project (cCURE > control), (F) project entirely of student design (cCURE > mCURE & control), (G) read primary literature (cCURE > mCURE & control), (H) write research proposal (cCURE > mCURE > control); and *Presenting results*—(I) present poster (cCURE > mCURE & control), (J) present orally (cCURE > mCURE & control). There were no significant differences between conditions for the statements in the data handling and analysis group or course structure group. For all the statistics see S2 Table.

The CURE survey [61] includes 25 student self-reported gains among elements that might be included in a course. We organized the statements into five groups of related items: knowledge of experimental outcomes (three statements), student involvement in research (seven statements), presenting results (three statements), course structure (eight statements) and data handling (four statements). We conducted an ANCOVA on each individual item and found statistically significant differences in 10 of the 25 learning gains (Fig 4 and S2A Table) and 15 non-significant differences (S2B Table). Faculty also reported on the amount that the same 25 elements were incorporated into their courses and their reports were descriptively compared to the students’ responses of the significant student statements (Fig 4 and S2 Table). Statistically significant differences in gains between conditions occurred in knowledge of experimental outcomes (three of three), student involvement in research projects (five of seven), and presenting results (two of three). No significant differences between conditions were observed in the course structure and data handling and analysis.

All three knowledge statements were significantly different between student groups (for all statistics see S2 Table). For a scripted lab or project in which the students know the expected outcome, (*p* < 0.001), the cCURE was significantly less than both mCURE (*p* < 0.001) and control (*p* < 0.001) (Fig 4A). For a lab or project in which only the instructor knows the outcome, (*p* = 0.001), the control was significantly greater than both cCURE (*p* = 0.002) and mCURE (*p* = 0.016) (Fig 4B). For a lab or project where no one knows the outcome, (*p* < 0.001), all were significantly different from each other (*p* < 0.001) with cCURE greater than mCURE greater than control (Fig 4C). Descriptively, the faculty agreed with the students in how they used these learning activities in their courses Fig 4A–4C). Statistical significance of student response data is represented as standard error, which was not calculated for faculty response data.

Five of the seven student involvement in research projects statements were significantly different between groups (for all statistics see S2 Table). For a project in which students have some input into the research process and/or what is being studied, (*p* < 0.001), the cCURE was significantly greater than both mCURE (*p* = 0.014) and control (*p* < 0.001) (Fig 4D). For become responsible for part of a project *(p* < 0.001), the cCURE was significantly greater than control (*p* < 0.001) (Fig 4E). For project entirely of student design *(p* < 0.001), the cCURE was significantly greater than mCURE (*p* = 0.015) and control (*p* < 0.001) (Fig 4F). For read primary scientific literature (*p* = 0.001), the cCURE was significantly greater than mCURE (*p* = 0.004) and control (*p* < 0.002) (Fig 4G). For write a research proposal *(p* < 0.001), the cCURE was significantly greater than mCURE (*p* < 0.001) and control (*p* < 0.001) and the mCURE was significantly greater than control (*p* = 0.018) (Fig 4H). Descriptively, the faculty agreed with the students for three of these statements about how they used these learning activities in their courses (Fig 4E, 4G and 4H). For the statements about students having some input (Fig 4D) and students entirely design project (Fig 4F), the control faculty score these lower than mCURE or cCURE faculty whereas the control students’ scores are equivalent to scores of mCURE students. This could be due to different interpretations between faculty and students about what constitutes experimental design.

Two of the three presenting results statements were significantly different between groups (for all statistics see S2 Table). For present posters (*p* < 0.001), the cCURE was significantly greater than mCURE (*p* < 0.001) and control (*p* < 0.001) (Fig 4I). For present results orally (*p* < 0.001), the cCURE was significantly greater than mCURE (*p* < 0.001) and control (*p* < 0.001) (Fig 4J). In this category there was a descriptive discrepancy between how the faculty reported using these learning activities and students reported learning from them. Faculty in the mCURE reported using more poster presentations than cCURE faculty, but students in the cCURE reported more learning from the poster presentations. Presenting orally was compatible between students’ reported learning and faculty usage. It’s possible that students viewed presenting a poster as also presenting orally, while faculty distinguished poster presentations and oral presentations.

We ran a two (URM vs. White/Asian) by three (cCURE, mCURE, control) ANCOVA of all 25 student learning activities across the three conditions (S3 Table). There was no significant interaction between URM status and condition for any statement indicating that there was no difference between conditions by URM status.

### Student learning outcomes

Student outcomes included several measures of learning (overall CURE evaluation, EDAT, TOSLS, CURE benefits), attitudinal changes (positive/negative attitudes, beliefs about science/science learning, the STEM Career Interest Scale, plans to conduct future research), and distal measures (GPA, STEM retention). Those results are organized below by each measure reporting the overall results and then the URM results.

#### Overall evaluation

**Fig 5:**
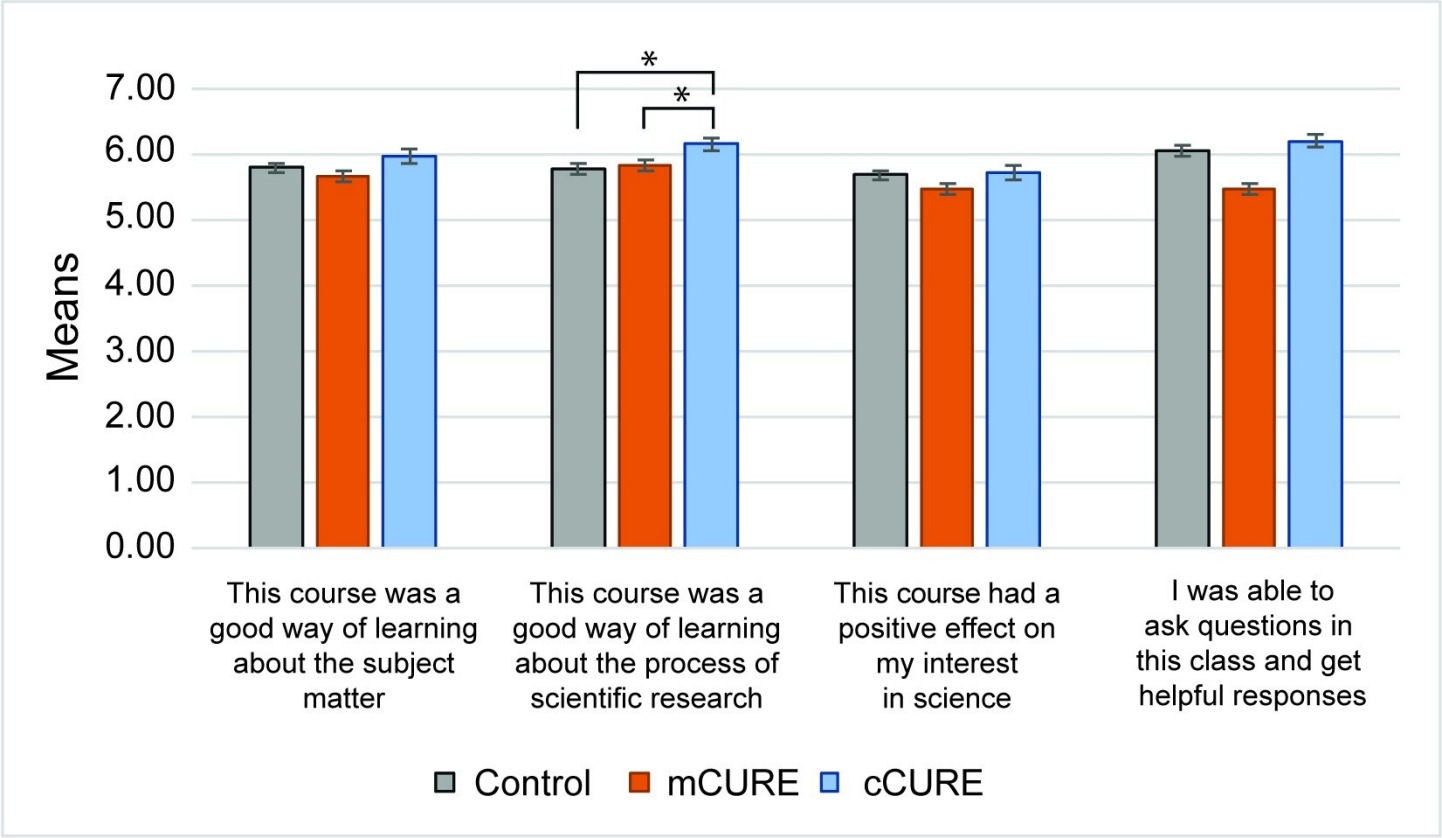
cCURE students report greater course satisfaction, while URM students report greater course satisfaction than White/Asian students in all conditions. Students reported their experiences with the course at the end of the semester. Statements were scored 1 (Strongly Disagree) to 7 (Strongly Agree). ANOVAs, with a Bonferroni correction for the four items tested (*p* < 0.0125), found the statement “This course was a good way of learning about the process of scientific research” rated significantly higher in the cCURE than the mCURE and control laboratories. See S10 Table for results. * *p* < 0.05.

There were four overall evaluation statements in the CURE survey which were analyzed individually. To correct for Type I errors, the critical alpha value was adjusted using a Bonferroni correction to *p* < 0.013. One-way ANOVAs found a significant difference in one statement: “This course was a good way of learning about the process of scientific research” (*p* < 0.001) (Fig 5). A Tukey post hoc test found the responses to this statement in the cCURE condition were significantly greater than the mCURE (*p* = 0.005) and control conditions (*p* < 0.001). Two (URM vs. White/Asian) by three (cCURE, mCURE, control) mixed factorial ANOVAs found a significant main effect for URM status for all four statements. However, there was no interaction effect for all four statements, indicating that URM students reported higher overall course evaluation than White/Asian students in all conditions, but that the overall evaluation reported by URM students was not different between any course type (cCURE, mCURE, and control). See S10 Table for complete statistical results.

#### Learning gains

A one-way ANOVA of the composite mean score of all 21 items indicated no significant differences in ratings of learning gains between the three conditions (*p* = 0.302) (S4 Table). A two (URM vs. White/Asian) by three (cCURE, mCURE, control) ANOVA was run to compare ratings of learning gains across the three conditions and URM status (S4 Table). There was a significant main effect of URM status (*p* < 0.001) where URM students reported greater benefits than White/Asian students regardless of condition. However, there was no significant interaction between URM status and condition (*p* = 0.602), indicating that the URM students self-reported learning gains did not differ by course condition.

**Fig 6:**
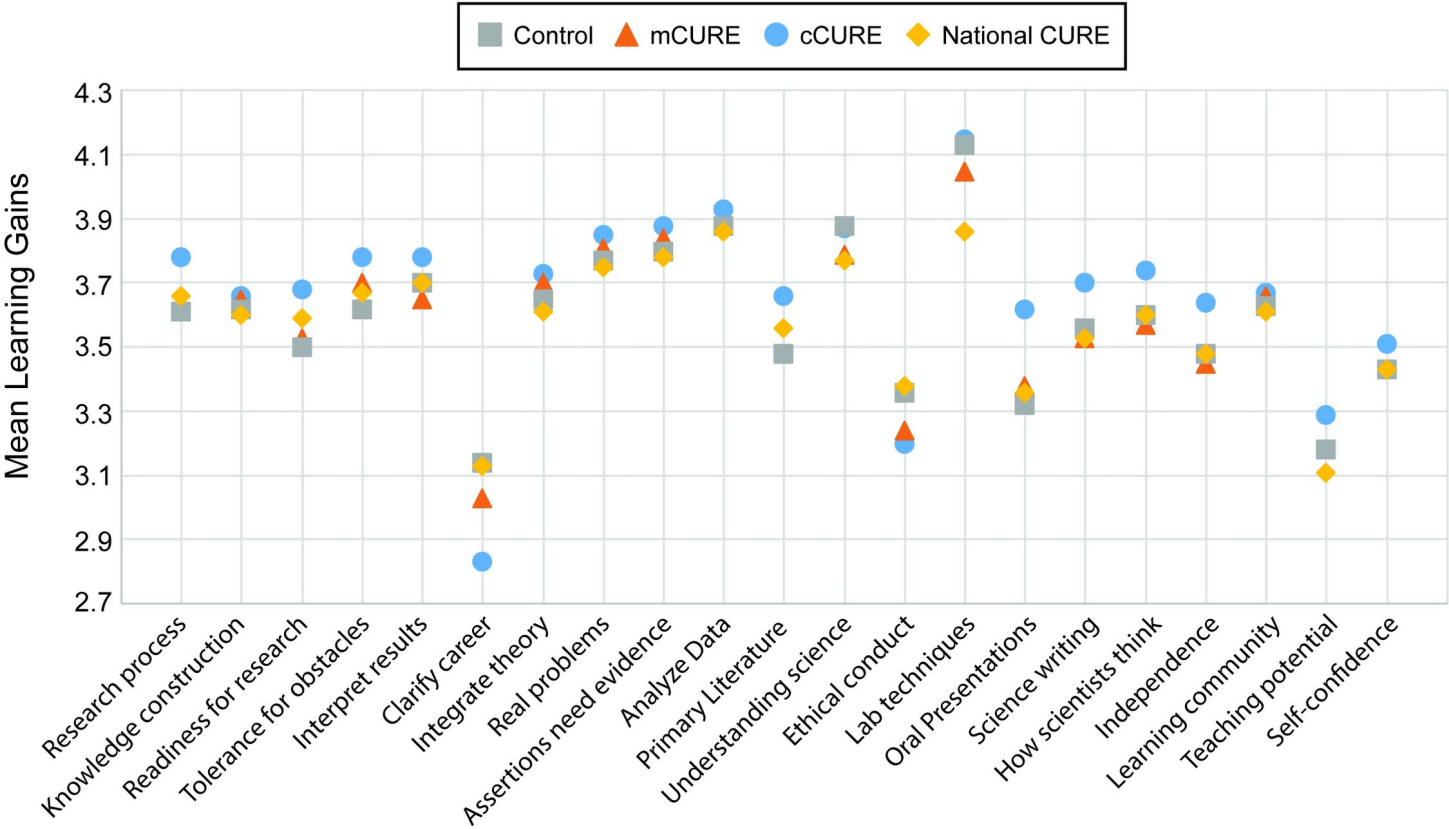
CURE students self-reported learning gains are not significantly different from control courses. Students self-reported learning gains on 21 items at the end of the semester from 1 = “No gain or very small gain” to 5 = “Very large gain.” A national dataset of CURE responses [64] is included. ANOVAs found no significant differences in student self-reported learning between the three conditions. Thirteen items in the national CURE dataset (yellow dots) fall within the range of the control, mCURE, and cCURE courses in the MCC dataset. See S4 Table for complete results.

Comparing the MCC data to national CURE survey data [64] indicates descriptive differences between conditions for some learning gains statements (Fig 6). Clarification of career path is lower for cCURE than mCURE, control, or national data sample. All MCC students, regardless of condition, indicated higher learning of laboratory techniques than the national sample. The cCURE students also indicated higher learning gains in oral presentations than those in mCURE or control courses or the national dataset. Almost two-thirds of the national CURE data fall within the range of the MCC control, mCURE, and cCURE data. Importantly, ANOVAs of each of the 21 individual statements (Bonferroni correction, *p* < 0.002) showed no significant difference between the three MCC conditions for any learning gain statement (S4 Table).

#### Experimental design

**Fig 7:**
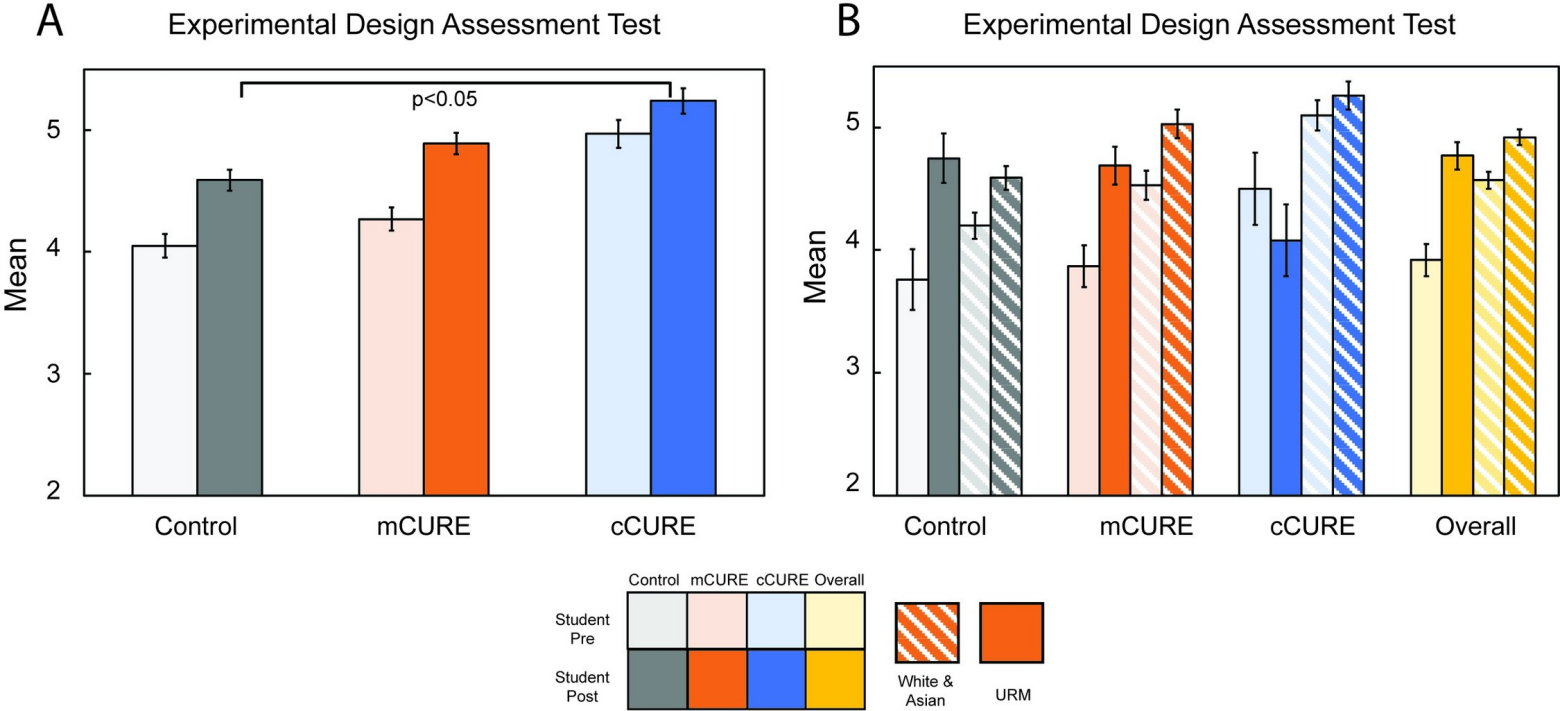
cCURE students score significantly higher on experimental design regardless of URM status. The experimental design assessment test (EDAT) [60] was given at the beginning and end of each semester. Responses were scored from 0 to 10 and an ANCOVA controlling for pretest scores was used to compare conditions. (A) Students in the cCUREs scored significantly higher than students in control courses, while students in the mCURE were not significantly different from either the control or cCURE students. (B) There was no significant difference in the performance of students by URM status within or between conditions. Statistical significance is represented as standard error (S.E.). See S11 Table for full results.

An ANCOVA controlling for EDAT pretest scores found a significant difference between conditions on EDAT posttest scores (*p* < 0.05) (Fig 7A and S11 Table). Simple contrasts found that students in cCUREs scored significantly higher on the posttest after controlling for the pre-test score than students in control courses (*p* < 0.05). Students in the mCURE post-test scores after controlling for pre-test scores were not significantly different than either control or cCURE students. A two (URM vs. White/Asian) by three (cCURE, mCURE, control) ANCOVA was run to examine differences in posttest scores while controlling for pretest scores (Fig 7B and S11 Table). There was no significant main effect for URM status (*p* = 0.585) and no significant interaction between URM status and condition (*p* = 0.268), indicating that URM students performed similarly to White/Asian students regardless of condition.

#### Scientific literacy

An ANCOVA controlling for TOSLS pretest scores found no significant differences in TOSLS posttest scores between conditions (*p* = 0.169) (S5 Table). A two (URM vs. White/Asian) by three (cCURE, mCURE, control) ANCOVA was run to examine differences in posttest scores while controlling for pretest scores. There was no significant main effect for URM status (*p* = 0.229) and no significant interaction between URM status and condition (*p* = 0.216) (S5 Table).

### Attitudinal student outcomes

#### Positive/negative CURE attitudes

**Fig 8:**
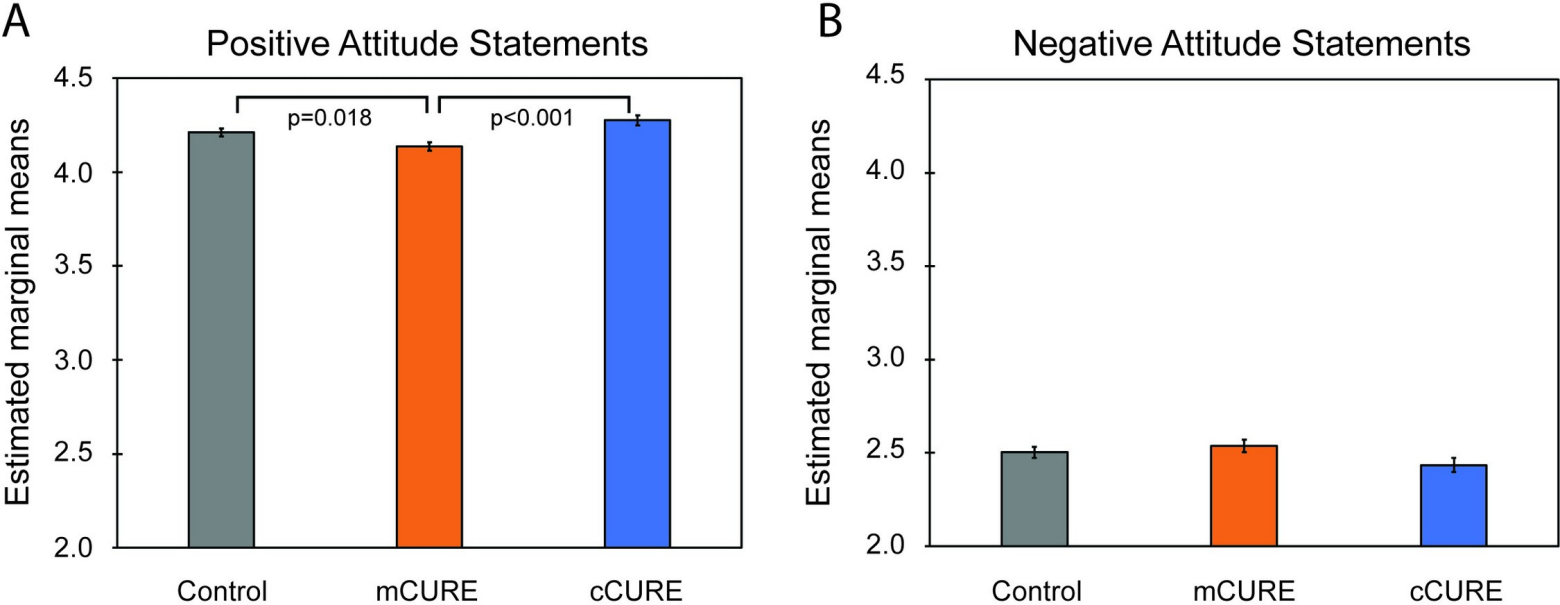
Students in mCUREs have the lowest positive attitudes towards science while there are no differences between conditions for negative attitudes. Students responded to (A) five statements that reflect a positive attitude and (B) six items that reflect a negative attitude towards science. Items were scored on a scale of 1 (Strongly Disagree) to 7 (Strongly Agree) on the pretest and 1 (Strongly Disagree) to 5 (Strongly Agree) on the posttest. An average composite score was calculated for each scale. An ANCOVA to compare between conditions while controlling for pretest scores was run. Students in the mCURE had significantly lower positive attitudes towards science than the cCURE and control groups. There were no differences between groups on negative attitudes. Statistical significance of student response data is represented as standard error (S.E.). See S6 Table for complete results.

For positive attitudes, an ANCOVA controlling for pretest ratings found significant differences in posttest scores by condition (*p* < 0.001). Pairwise comparisons of the estimated marginal means using a simple contrast found that ratings in the mCURE condition were significantly lower than ratings in both the cCURE (*p* < 0.001) and control conditions (*p* = 0.018) (Fig 8A and S6 Table). A two (URM vs. White/Asian) by three (cCURE, mCURE, control) ANCOVA controlling for positive attitudes at pretest found no significant main effect of URM status (*p* = 0.646) and no significant interaction between URM status and condition (*p* = 0.548) (S6 Table). For negative attitudes, a one-way ANCOVA controlling for pretest ratings found no significant differences in posttest scores by condition (*p* = 0.118) (Fig 8B and S6 Table). A two (URM vs. White/Asian) by three (cCURE, mCURE, control) ANCOVA controlling for negative attitudes at pretest found no significant main effect of URM status (*p* = 0.781) and no significant interaction between URM status and condition (*p* = 0.362) (S6 Table).

#### Beliefs about science/science learning

ANCOVAs controlling for pretest ratings including a Bonferroni correction of *p* < 0.005 found no significant differences in posttest ratings between conditions on 11 individual statements about students’ beliefs about how science is conducted and how it is taught (S7 Table). Two (URM vs. White/Asian) by three (cCURE, mCURE, control) ANCOVAs found no differences between URM and White/Asian students on any of these statements nor any significant interactions between URM status and condition (S7 Table).

#### STEM support, career interest, and importance

**Fig 9:**
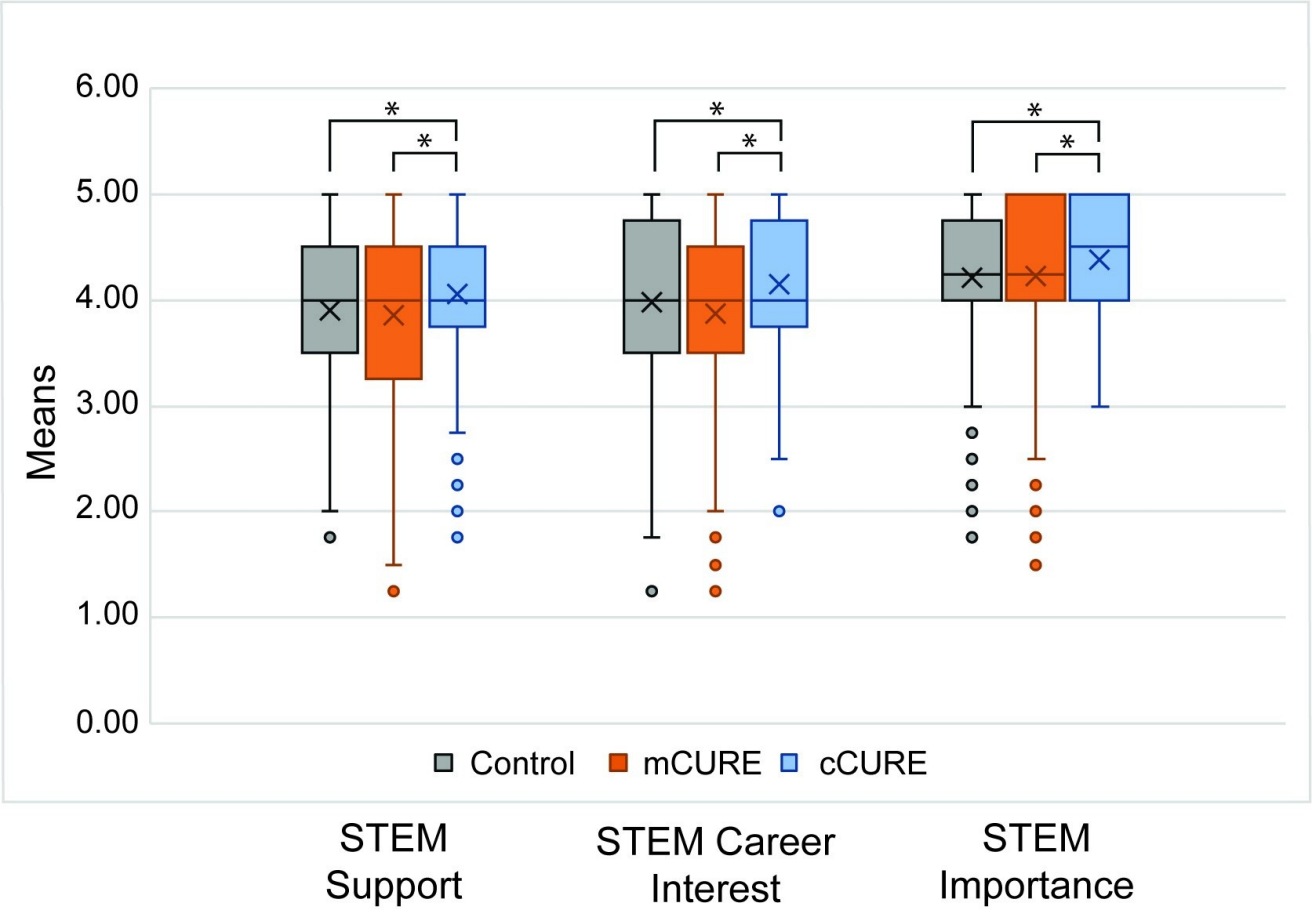
Students in cCUREs report the highest levels of STEM support, career interest, and importance. On the posttest, students responded to four items each about STEM support, STEM career interest, and importance and a scale of 1 (Strongly Disagree) to 5 (Strongly Agree). An average of each scale was calculated, and the three conditions were compared with an ANOVA. Tukey post-hoc tests found cCURE students reported significantly more STEM support, career interest, and importance than mCURE and control students. See S8 Table for complete results. *p < 0.05.

An ANOVA found significant differences in STEM support, STEM career interest, and STEM importance across conditions (support, *p* = 0.006; career interest, *p* < 0.001; importance, *p* = 0.001). Tukey post-hoc tests found that students in the cCURE condition reported significantly greater STEM support, STEM career interest, and STEM importance than students in both the mCURE (support, *p* = 0.006; career interest, *p* < 0.001; importance, *p* = 0.005) and control conditions (support, *p* = 0.030; career interest, *p* = 0.013; importance, *p* = 0.001) (Fig 9 and S8 Table). Two (URM vs. White/Asian) by three (cCURE, mCURE, control) ANOVAs were run to examine differences in STEM support, career interest, and importance across the three conditions and URM status (S8 Table). For STEM support, there was no significant main effect for URM status (*p* = 0.226) and no significant interaction between URM status and condition (*p* = 0.396). For STEM career interest and STEM importance, there was a significant main effect of URM status (career interest, *p* = 0.040; importance, *p* = 0.021) where URM students reported significantly greater STEM career interest and importance than White/Asian students. However, there was no significant interaction between URM status and condition (career interest, *p* = 0.439; importance, *p* = 0.397).

#### Plans to conduct research

**Fig 10:**
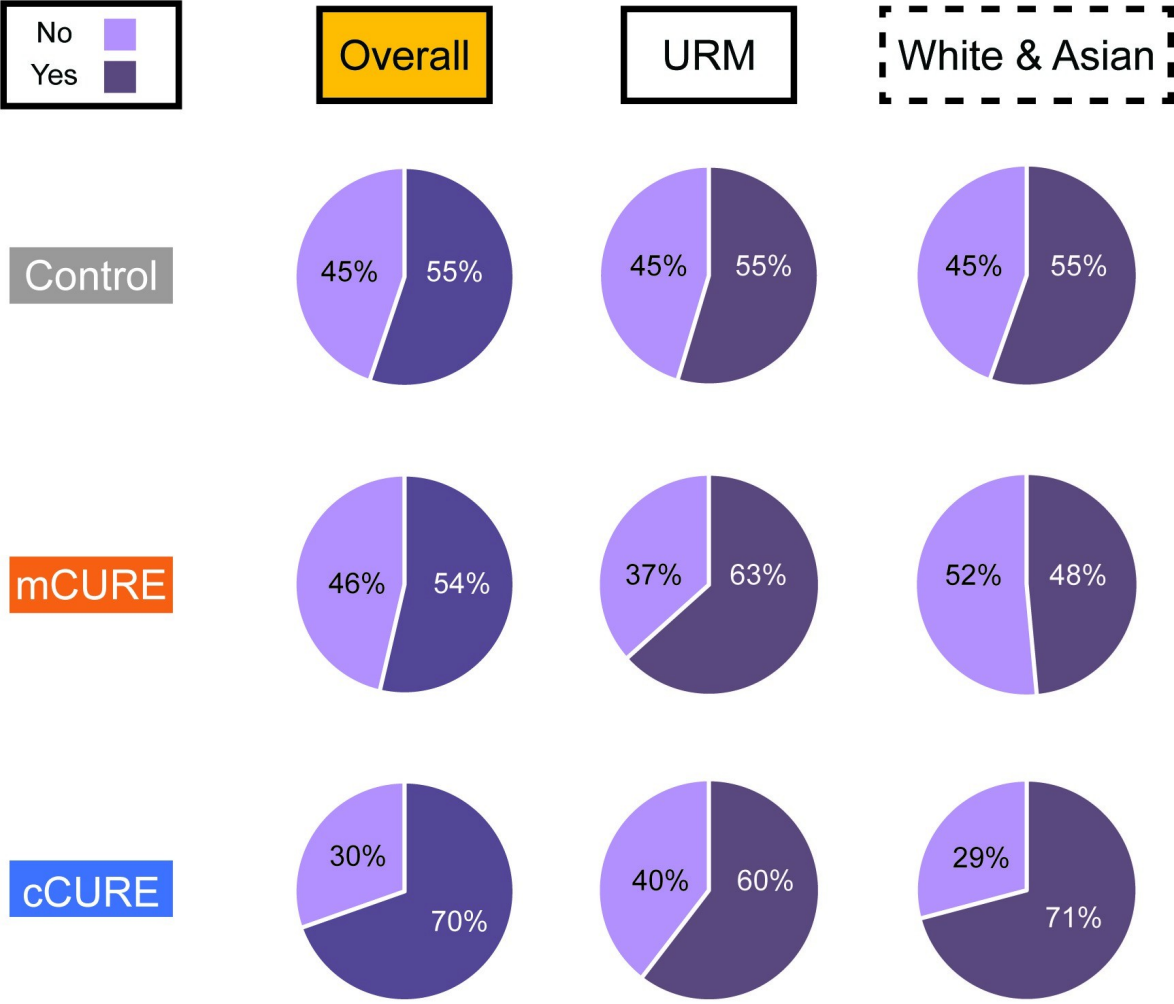
More students in the cCURE report plans to conduct undergraduate research while more URM students in the mCURE report plans to conduct research. At the end of the semester, students reported on their plans to conduct research in the future and conditions were compared using χ^2^ analysis. Significantly more cCURE students planned to conduct research in the future than in the mCURE or control students. Student plans to conduct research by URM status and condition found more URM students in the mCURE planned to conduct research than White/Asian students in the mCURE. There was no significant difference by URM status in the cCURE or control conditions. See S12 Table for complete results.

On the posttest, students indicated their plans to conduct research in the future (Fig 10 and S12 Table). A chi-square test of independence found a significant difference in the distribution of responses across conditions (*p* < 0.001). Students in the cCURE condition were more likely to indicate they wished to conduct research in the future than students in the mCURE and control conditions. Chi-square tests of independence were conducted to determine if there were differences in URM students’ plans to conduct research both within and across conditions (Fig 10 and S12 Table). Overall, there were no differences in plans to conduct research when comparing all students by URM and White/Asian status (*p* = 0.716). While there were no differences in URM and White/Asian students’ plans to conduct research within the control (*p* = 0.888) and cCURE conditions (*p* = 0.154), there were significant differences in the mCURE condition (*p* = 0.012). URM students in mCUREs were more likely to report that they planned to conduct research in the future than White/Asian students in mCUREs (Fig 10 and S12 Table).

#### GPA and one-year retention

An ANOVA found no significant differences in one-year post-course cumulative GPA between conditions (*p* = 0.709) (S9 Table). For one-year retention, a chi-square test of independence found no significant differences in retention between conditions (*p* = 0.054) (S9 Table). Due to low sample size, URM vs. White/Asian analyses of these parameters were not conducted.

## Discussion

### Less time spent in the CURE correlates with lower reported amount of CURE elements and activities

Control courses contain the data collection and analysis elements, while mCUREs have more elements of a CURE experience, and cCUREs include all CURE activities and elements. We assessed whether students and faculty identified differences in lab course elements between the conditions. Students in mCUREs perceive discovery/relevance and iteration to a significantly greater degree than those in control courses but the duration of the experience was important; they perceived less of these elements than students in cCUREs. Students in mCUREs did not perceive more collaboration than students in control courses, though students in cCUREs perceived significantly more collaboration than students in control courses (Fig 2). Faculty in the cCUREs reported using more CURE elements than those in mCUREs. Control faculty largely reported using laboratory elements related to data collection and analysis (Fig 3). When evaluating the same 25 CURE activities, faculty and student results are mostly consistent with the student LCAS and faculty elements results (Fig 4 and S2 Table). These results establish a difference in how students were taught within the cCURE, mCURE, and control courses. Most prior studies [27, 42–44, 46] have relied on descriptions of the course designers’ intentions. A few studies have shown differences in students’ perception of CURE versus control laboratory courses [58, 65]. As done in this study, Mader et al. [45] used student and faculty reports on the same elements but did not use multiple lines of evidence to examine both the students’ experience and faculty reports of CURE elements included in the course. Without knowledge of the actual implementation of CURE elements, conclusions about impacts have limited generalizability [66]. This is the first study to use multiple lines of evidence that these three conditions do vary in the students’ exposure to the elements of a CURE, increasing the generalizability of these results.

### Laboratory condition does not impact URM students’ experience of the laboratory course

This is the first study to use the LCAS to examine URM student experiences of laboratory courses. We assessed whether there was a difference in URM students’ perceptions of laboratory course elements in control laboratory courses, mCUREs, and cCUREs, as compared with White/Asian students. In all conditions, URM students did not perceive differences in discovery/relevance or collaboration compared to White/Asian students. However, URM students reported more learning activities and iteration than White/Asian students in all conditions. Across the three conditions, URM students perceived nine of the 25 student learning activities (S3 Table) and the CURE element iteration (S1 Table) as occurring more regularly than their White/Asian counterparts. For the other 16 learning activities, collaboration, and discovery/relevance, URM student perceptions were similar to White/Asian students. Overall, the consistency of the differences across both instruments indicates that these differences between URM and White/Asian students are not a CURE-specific phenomenon.

### A longer CURE contributes to improvements in some student learning areas

We evaluated the difference in student outcomes between control laboratory courses, mCUREs, and cCUREs. The most impactful means for students to learn about the process of scientific research and experimental design is a cCURE, but measures of self-reported learning gains and scientific literacy were not impacted by CURE exposure. Students reported that cCUREs were a better way to learn about the process of research than mCUREs or control courses (Fig 5), but they reported no differences between conditions in learning about the subject matter, getting answers to questions, or interest in science. The mCURE and control students generally scored three of the four overall evaluation statements lower than cCURE students. The fourth statement, “asking questions,” should not depend on the type of laboratory course as it is an indication of the students’ perceptions that the instructor was helpful, which can occur in all types of laboratory courses. In a large dataset such as this one, this statement serves as an internal control indicating that students are answering consistently across conditions.

There were no significant differences between the three conditions in students self-reported learning gains (Fig 6 and S4 Table). This contrasts with two studies comparing CURE length [45, 46] which showed learning gains from GEP and a consortium of CURE faculty were higher than faculty mentored research and/or other CUREs. In contrast to the MCC students, when Shaffer et al. [46] averaged the student learning gains for GEP students, they saw significantly lower scores when students had 11–24 hours in the GEP (similar to an mCURE) and students who had more than 36 hours in the GEP (similar to the cCURE). Mader et al. [45] compared CUREs of less than one semester to semester-long CUREs across several types of CUREs including SEA-PHAGES, GEP, and faculty developed CUREs. Analyzed individually, all learning gains were significantly higher in the semester-long CUREs than ones less than one semester. Neither study included control laboratories.

The MCC control data presented here is contradictory to the original CURE survey study which showed substantial descriptive differences between CURE and control students, although statistical comparisons were not reported [61]. However, when comparing MCC students to a national dataset of more than 11,000 CURE students [64] 13 of the 21 learning gains reported in the national dataset fall between the minimum and maximum MCC reported learning gains (Fig 6). This is the second study to report comparisons of control and CURE laboratory courses with the CURE survey learning gains statements. Both projects were implemented in a wide variety of biology and chemistry courses [46, 61], so the student populations should be similar. However, there may have been differences in the type of experience students received in the control groups of these two studies. MCC control groups were generally taught by the same faculty who taught the CURE and were always laboratory classes. These laboratories often included substantial experimental design for projects where the instructor knew the outcome (and the students could search the literature for the answer). This means that, while many MCC control courses lacked discovery/relevance, they often included substantial iteration and collaboration, though significantly lower than in the cCURE (Fig 2). Many of the learning gains statements are relevant to either of those conditions. Conversely, the GEP control courses could include either laboratory or lecture courses described as “courses without research” [61, pg. 684].

Recent research shows higher learning on objective tests of student knowledge when comparing students within one type of CURE to control students [20, 25, 44, 46, 52, 67–76]. When comparing different CUREs, it is difficult to find objective measures of student knowledge that are not content specific. This study included two objective measures of student learning (EDAT and TOSLS). Controlling for pre-test score students in MCC, cCUREs showed significantly more learning about experimental design compared to those in the control (Fig 7A). Compared to CURE-specific data, this is a striking response since the EDAT measures general experimental design, which was not necessarily aligned to the types of experimental design carried out in the various MCC CUREs. However, after controlling for pre-test score, there was not a significant difference between the students in the mCURE and control or the cCURE, indicating that the students in the mCURE bridged the significant gap between cCURE and control. There were no differences between conditions on science literacy, in fact students’ TOSLS scores dropped slightly over the semester in all conditions (S5 Table). Motivation and survey fatigue are possible reasons for the lower scores across all conditions. All posttest assessments were given at a similar time and, while the other posttest questions were mostly Likert scale, the TOSLS (28 questions) assesses skills and requires students to interpret text and figures rather than give opinions. Similarly, the EDAT is a free response test asking students to design an experiment. The post survey questions were not given for a grade, but instructors were encouraged to give some credit for finishing the post survey questions, providing motivation to finish, but not necessarily for students to do their best. While the TOSLS has rarely been used in CURE research, Sandquist et al. [77] also found no significant pre-/posttest differences using a reduced version of the TOSLS (13 questions). They used the reduced test after finding that students rushed through the full TOSLS at the end of the semester, further supporting the possible lack of motivation and survey fatigue contributing to the TOSLS results for MCC students. Given the possibility that students’ motivation on posttests was related to finishing, but not necessarily doing well, and that students did not study specifically for the EDAT and TOSLS questions, the significant improvement in their EDAT scores becomes even more important. This demonstrates a change in students’ core understanding of the experimental design process.

### A longer CURE experience is required to affect student STEM support, career interest, and importance, but does not impact students’ attitudes and beliefs about science

Students in cCUREs reported higher STEM support, career interest, and importance than those in mCURE and control courses (Fig 9). These elements support proposed mechanisms by which CUREs influence students’ increased persistence in STEM [21, 35]. When students feel supported in their desire to pursue STEM, participate in a course that increases their interest in science and influences their beliefs about the importance of STEM. This should lead to an increased desire to obtain a STEM degree. This also supports research showing increased interest in graduate school [78], improved course pass rates [79, 80], and student retention [15, 40, 47, 53, 79, 80] for CURE students compared to control students.

There was no difference in negative attitudes (Fig 8B) or beliefs about science (S7 Table) between conditions. However, students in the mCURE had less positive attitudes than cCURE or control students (Fig 8A). Lopatto and Jawalski [64] also found little difference in students’ pre/post science attitudes in CUREs, but their average scores for post-course attitudes in CUREs were similar to our results (positive = 4.13, negative = 2.51) with the overall mean of the mCURE is slightly higher (mCURE positive = 4.15) than CURE students in their data. These data are also consistent with the stability of attitudes towards engineering [81]. Attitudes may, therefore, be a more stable construct than can be affected in one semester of a single course.

### A longer CURE positively impacts students’ intentions to conduct research in the future, but not future GPA or retention in the sciences

Students in the cCURE were more likely to plan on doing future research than mCURE or control students (Fig 10). This is consistent with prior research indicating that CUREs increase interest in future undergraduate research compared to control courses [25]. There were no differences in GPA or retention between conditions (S9 Table). This is not surprising considering Rodenbusch et al. [37], who found that increased retention required three semesters of a CURE. The limited research [37, 40, 50] on the lack of impact on future GPA is also supported by this study. Here, GPA was only examined for students enrolled one-year post CURE (excluding students who graduated or transferred/changed institutions). Additionally, the GPA and retention data came from a small subset of the sample population and the sample size for the cCURE condition was low (S9 Table). GPA and retention data were collected from offices of institutional research at the institutions participating in this study. Despite prior agreements, institutional response rates were low, possibly due to differences on the type of institutional research data they would provide.

### Shorter CURE experiences had a greater impact on URM students in terms of their intention to conduct research than White/Asian students

URM students who have experienced an mCURE said they were more likely to conduct research than White/Asian students in mCUREs. This difference between URM and White/Asian students does not appear in control or cCURE courses. While there is no direct comparison of URM status on future research plans, Tootle et al. [42] also found increased interest in future research after a short CURE module at a historically black college or university. However, there was no control comparison in that study. Since immersion in undergraduate research is a key experience for increased graduation rates and acceptance to graduate and professional schools for URM students [12, 14, 15, 48, 82, 83], this finding has significant implications for improving URM representation in STEM fields.

### In general, laboratory course type did not have an impact on URM students compared to White/Asian students

We compared the impact of URM students in the three conditions to determine if there was a differential impact compared to White/Asian students. URM students reported greater learning of subject matter and the process of research, increased interest in science, and ability to ask questions/get help than White/Asian students regardless of condition. URM students also reported greater learning gains than White/Asian students regardless of condition (S4 Table). This contrasts with Shaffer et al. [46], who did not find any differences in self-reported learning gains between demographic groups (first-generation, URM, nontraditional, and commuter) in their study of the GEP consortium. In our study, URM students reported more STEM career interest and STEM importance across all conditions (S8 Table).

URM students performed equally well on experimental design (Fig 7B) and scientific literacy (S5 Table) as White/Asian students regardless of condition. This is consistent with Ing et al., [50] who found no difference in course grades between URM and White/Asian students. It is also consistent with Rodenbusch et al., [37] who found no impact by demographics on student outcomes in CUREs. There was no difference between URM and White/Asian students on beliefs about science and science learning (S7 Table), positive or negative attitudes (S6 Table), or STEM support (S8 Table) regardless of condition. While there are no direct comparisons for these items in the URM CURE literature, these results are supported by other indicators of motivation and attitudes [30, 35].

### Considerations for generalizability

There are several considerations for the generalizability of this study to other populations. The reliability of the EDAT scoring is lower than the published inter-rater reliability (*r* = 0.835, *p* < 0.001, [60]). This warrants caution when interpreting the results. However, lower reliability may increase Type II errors (not detecting a relationship that exists) in subsequent statistical testing [84] which offsets the low reliability. Since all these scales relied on post-semester scores, they do not account for any students who dropped the class who might have had lower scores than those who completed the course. This was true for all conditions including the control. With evidence of higher pass rates in CURE courses, this should also have increased the likelihood that we would see a false negative (Type II error). There is also the possibility that URM students and mCURE results might be conflated since there were more URM students in mCUREs than cCURE or control classes (Table 1). However, it is vitally important to the impacts on different student demographics if URM representation in STEM careers is to be increased.

Due to an error on the pretest, the positive and negative scales (Fig 8) were run on a scale of 1 to 7 on the pretest and 1 to 5 on the posttest. We used the pretest score as a covariate, so the statistical relationships are sound. However, this made comparing our results to other studies more difficult. Additionally, the pre and post-survey were either assigned as extra credit or as an assignment for completion, so the students were incentivized to do the assessment, but not necessarily take the time to do their best. The posttest was fairly long which might have contributed to survey fatigue. This might also explain why TOSLS scores were lower at the end of the semester since it has 28 text heavy questions. Student assessments of their own learning gains do not necessarily correlate with actual learning as measured on objective tests of learning [85, 86]. This may be why there was no difference in student learning gains between conditions (Fig 6). However, Lopatto [87] found a correlation between student self-reported learning gains and students who planned to continue their coursework into graduate school. It is also true that learning gains from these CUREs were in areas other than those included in this research.

### Implications for future research

In general, CUREs should improve students’ understanding of the nature of science, experimental design, data collection, analysis, and presenting scientific results. The challenge is to find objective measures that are as context independent as possible. Unfortunately, learning is often context dependent and students find it difficult to apply what they learned in one context to another completely different setting. We used two general objective tests of learning about experimental design (EDAT) and scientific literacy (TOSLS), both of which are completely divorced from the context of proteins and enzymes. The significant differences in students’ experimental design abilities shown between cCURE and control courses is then especially important. During the inception phase of the MCC, these were the only choices of objective measures available, and few if any have been developed since. In CURE research, there is a great need for the development of such measures which would allow comparisons across many different CUREs and CURE studies. The same is true for attitudinal measures. The CURE/SURE survey asks students about attitudes, but is not designed to measure specific attitudinal variables such as self-efficacy, science identity, and motivation. Several measures of attitudinal variables are available (e.g., CLASS [88], MSLQ II [89]). Most of these have been designed to measure these variables in other student populations, but can be applied to CURE students. One CURE specific instrument is the Persistence in the Sciences [90] which combines several validated attitudinal measures with others that were specifically designed for CUREs. Again, it would be easier to compare studies if one or more of these were used more consistently across studies.

Large numbers of CURE students doing different CUREs allows generalizability about CURE impacts and characterizations of multiple demographic student groups, institution types, and CURE types. While studies of network CUREs (e.g., GEP, SEA-PHAGES) allow for disaggregation by demographic groups and institution types, they are limited by utilizing the same CURE in all conditions. While this allows for easier objective tests of learning, it also sets limits on instructor choices, course types, and typical levels of students (upper- or lower-division) courses. Using different CUREs allows greater generalizability because the content, implementation, and course levels can vary greatly. The disadvantage for this research is that finding objective measures of learning that can be generalized to multiple CURE contexts is difficult. Unfortunately, while students’ self-reported learning gains are easier to compare across different types of CURES, there is evidence that students might not be good at estimating what they have learned when compared to objective measures of student learning [85, 86].

Another factor for future research is using multiple measures of the fidelity of implementation of the CURE. As we did here, researchers need to compare what the faculty think they did in their class with what the students experienced. What faculty think they have implemented doesn’t always correspond to what they really did [91], so comparing student experiences with faculty reports is important. Future studies of multiple CUREs should include measures of both faculty perceived and student reported implementation of the CUREs. As done in Corwin et al. [38], these measures can then be linked to specific outcomes giving us a more nuanced understanding of the CURE elements that lead to specific outcomes.

This is the first large-scale study to compare CUREs less than a semester with semester-long CURES and non-CURE controls. In general, we found that students in mCUREs were more likely to be similar to the control than the cCURE. However, there are multiple instances where a shorter CURE experience embedded in a traditional course is the more feasible option. We were not able to measure all the possible outcomes of a CURE for students and it is possible that students in mCUREs benefited from other outcomes such as science identity, research self-efficacy, and project ownership. Future research also should consider the implications for student outcomes of experiencing multiple mCUREs within one course or over time in several semesters. There is preliminary evidence that student learning gains occur when doing several mCUREs in one semester [27], but no studies that look at longitudinal mCURE experiences.

There are few CURE studies that disaggregate their results by student demographics. We were encouraged to see that CUREs were at least as impactful for URM students as the White/Asian students in our study. The increased interest in future research for URM students created by participation in a mCURE warrants more investigation. Is this because there were more URM students in mCUREs, were more students in mCUREs in lower division classes, or some other reason? We did not collect data about course level for this study which would help disentangle this question. As mentioned in the introduction, there are very few CURE studies that generate data broken down by any student demographics. To increase participation of URMs in STEM careers, we first need to understand what experiences impact URM retention in STEM degrees. However, this requires a large study to have enough participants to break-down by student demographics. We chose to combine data from all students in various URM categories into one group and contrasted with White/Asian student data. Grouping students in just two categories does not give a nuanced picture of how CUREs are impacting different groups of students. Nor did we have a chance to isolate the effect of the CUREs on students categorized in other ways, such as first-generation students, veterans, and other non-traditional students that also have lower STEM degree retention rates. Future research needs to investigate CURE impacts for students in these demographics.

### Implications for teaching and learning

Our findings indicate that, while students may not necessarily experience the entire spectrum of beneficial outcomes from a mCURE, these shorter CUREs do not negatively affect student outcomes, particularly among URM students. This is especially important for URM students, who are more likely to attend institutions that have less infrastructure available for implementing a cCURE [92, 93]. Faculty who have other constraints in their courses that make implementing a cCURE difficult or who are not able to change a whole course in one semester, can be confident that implementing a mCURE will help be beneficial for some students. However, when it is possible to implement a cCURE, the student learning and attitudinal outcomes are better. Faculty who wish to implement a CURE do not need to use extensive pedagogical assessment or compare student outcomes to other courses to be confident that changing a class to a CURE will improve their students’ outcomes. Arguments that science faculty do not have the expertise to successfully implement active learning strategies [94] are not supported by this study. Only one Discipline-Based Education Research faculty member (author—SEDP) had students who participated in this study (contributing less than 1% of the students in this dataset), indicating that pedagogical research expertise is not necessary to effectively implement CUREs. Importantly, implementing mCURE experiences can be an effective strategy in closing the opportunity gap. The significantly higher interest in future research exhibited by URM students compared to White/Asian students in mCUREs (Fig 10) is by itself reason enough to implement mCUREs where cCUREs are not feasible.

**Fig 11:**
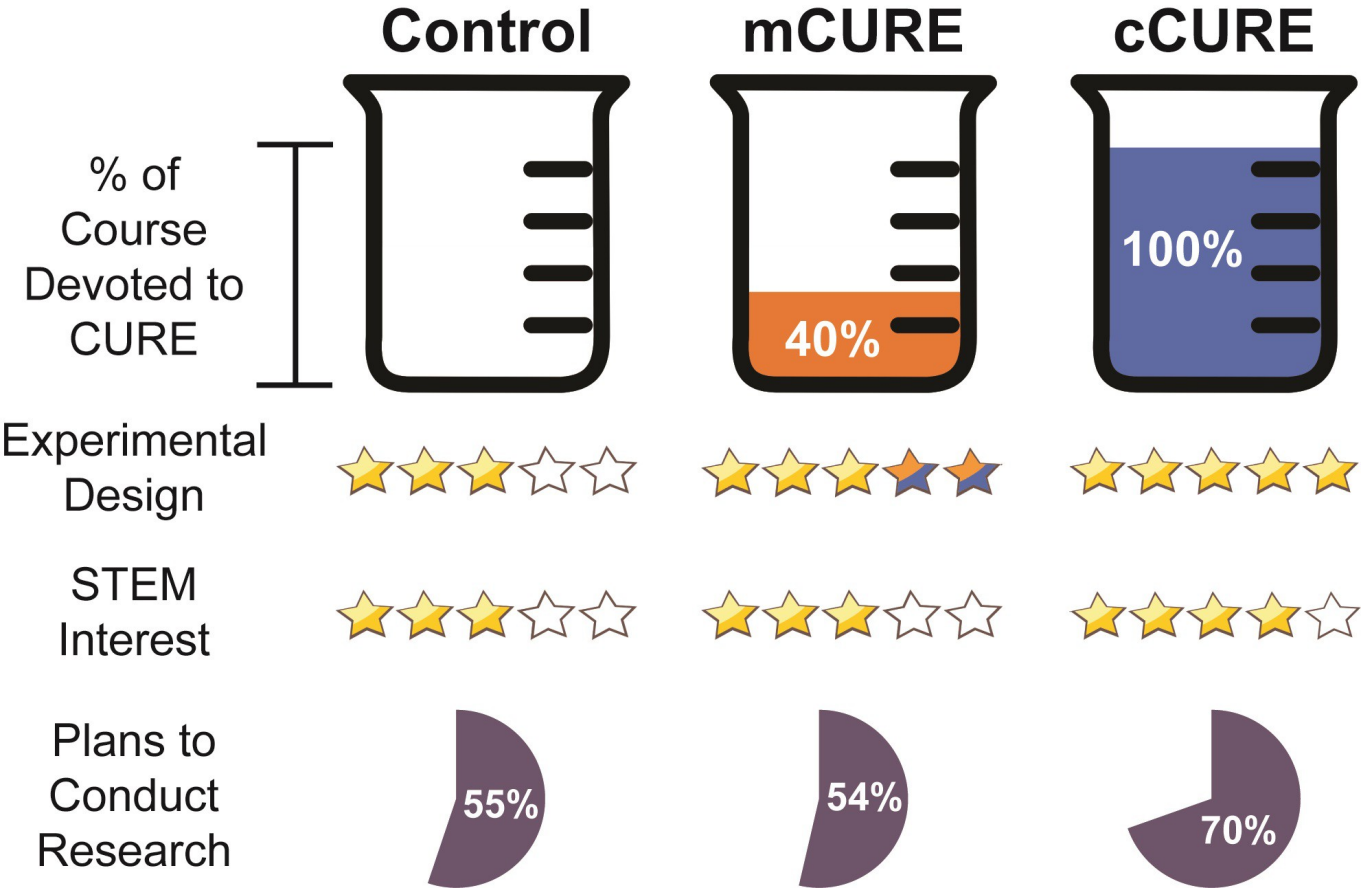
Overall student outcomes. The CURE courses included more time in the course devoted to elements of a CURE. Student improvement in experimental design was higher in cCUREs than controls, while mCURE students were not significantly different than either. STEM interest and plans to conduct future research were higher in cCUREs compared to mCUREs and controls.

One MCC goal was to implement protein-centric CUREs to improve student learning and attitudinal outcomes (Fig 11). Evidence supports that this goal was met. More than 1,000 students took part in MCC CURES across the U.S. over two years in entry level and advanced courses and across all types of institutions. The MCC continues to grow and add new faculty and their students. Student evidence from the MCC indicates that full-semester CUREs implemented by science faculty are effective compared to equivalent non-CURE courses, often taught by the same faculty. Importantly, evidence indicates that mCURES, which might be easier for some faculty to implement, particularly when first beginning to teach with CUREs, are at least as effective as control laboratories and equivalent with cCUREs in learning of experimental design. Short duration CUREs also provided significant interest in pursuing future research opportunities for URM students compared to White/Asian students. This finding is intriguing for the possibility of improving the diversity of STEM fields and needs further investigation. Finally, cCUREs provide many benefits to students compared to control courses across multiple different CUREs. The evidence from this study should be encouraging to faculty who wish to implement CUREs in their courses.

## Supporting information

S1 Table LCAS by CURE condition. On the post-test the students reported their experiences with three elements of a CURE. Iteration and discovery/relevance were comprised of five items scored 1 (Strongly Disagree) to 7 (Strongly Agree). Collaboration included six items scored using 1 (Never), 2 (One or two times), 3 (Monthly), and 4 (Weekly).
(DOCX)

S2 Table Student and faculty learning activities. On the pretest, students rated their experience with 25 activities that occur in science courses using a scale from 1 = “No experience or feel inexperienced” to 5 = “Extensive experience or mastered this element.” At posttest, students rated their learning gains related to each of the 25 activities using the scale 1 = “No gain or very small gain” to 5 = “Very large gain.” The 15 activities shown in this table were found to be not significantly different by CURE condition for students (Bonferroni correction p < 0.002 is significant). Faculty rated each item by 0 = “not applicable” to 3 = “major”.
(DOCX)

S3 Table Student learning activities by URM status. On the pretest, students rated their experience with 25 activities that occur in science courses using a scale from 1 = “No experience or feel inexperienced” to 5 = “Extensive experience or mastered this element.” At posttest, students rated their learning gains related to each of the 25 activities using the scale 1 = “No gain or very small gain” to 5 = “Very large gain.” Bonferroni correction *p* < 0.002 is significant.
(DOCX)

S4 Table CURE learning gains. Table A: CURE post learning gains by CURE condition. Table B: CURE learning gains by URM status and interaction of status/condition. Table C: CURE individual post learning gains (Bonferroni correction for 21 statements, *p*<0.002).
(DOCX)

S5 Table TOSLS data. Table A: TOSLS by CURE condition. Table B: TOSLS by URM status and interaction of status/condition.
(DOCX)

S6 Table Positive and negative attitudes by URM status. On the pretest, students responded to five items concerning their attitudes towards science using a scale from 1 = “Strongly disagree” to 7 = “Strongly agree.” On the posttest, students responded to the same five items using a scale from 1 = “Strongly disagree” to 5 = “Strongly agree”.
(DOCX)

S7 Table Beliefs about science and science learning. On the pretest, students responded to eleven items concerning their attitudes towards science using a scale from 1 = “Strongly disagree” to 7 = “Strongly agree.” On the posttest, students responded to the same eleven items using a scale from 1 = “Strongly disagree” to 5 = “Strongly agree.” Table A: Beliefs about Science and Science Learning by CURE Condition. Table B: Beliefs about science and science learning by URM status and interaction of status/condition.
(DOCX)

S8 Table STEM support, career interest, importance. Table A: STEM support, career interest, importance by CURE condition. Table B: STEM support, career interest, importance by CURE post benefits by URM status and interaction of status/condition.
(DOCX)

S9 Table GPA and retention. Data collected from institutional data. Not all institutions responded to request for this data. Table A: GPA. One year post-CURE cumulative GPA from students still enrolled (excludes graduates). Table B: Retention. One year post-CURE enrollment or graduation status. Students who graduated or were still enrolled were included.
(DOCX)

S10 Table Overall evaluation. Table A. Overall Evaluation. Table B. Overall Evaluation by URM status and interaction of status/condition. For the four statements, Bonferroni correction indicated that *p* < 0.013 was significant.
(DOCX)

S11 Table EDAT data. Table A: EDAT by CURE condition. Table B: EDAT by URM Status and Interaction of Status/Condition.
(DOCX)

S12 Table Plans to conduct research. Table A: Plans to conduct research by CURE condition. Table B: Plans to conduct research by URM status and interaction of status/condition. At the end of the semester, students were asked if they planned to conduct research in the future (yes or no).
(DOCX)

## References

[1] Fayer S, Lacey A, Watson A. STEM occupations: Past, present, and future. Spotlight on Statistics. 2017;1:1–35.

[2] Chen X. STEM attrition: College students’ paths into and out of STEM fields. Statistical Analysis Report. In: Statistics NCfE, editor. 2013.

[3] Seymour E, Hewitt NM. Talking About Leaving: Why Undergraduates Leave the Sciences. Boulder: Westview Press; 1997. 429 p.

[4] Thiry H, Weston TJ, Harper RP, Holland DG, Koch AK, Drake BM. Talking About Leaving Revisited: Persistence, Relocation, and Loss in Undergraduate STEM Education: Springer Nature; 2019.

[5] Excluded Asai D. J Microbiol Biol Educ. 2020;21: 10. 10.1128/jmbe.v21i1.2071 PMC7148151 32313599

[6] Kuh G, Kinzie J, Buckley J, Hayek J. What matters to student success: A review of the literature (Executive summary). Commissioned report for the National Symposium on Postsecondary Student Success. 2006.

[7] Russell SH, Hancock MP, McCullough J. Benefits of undergraduate research experiences. Science. 2007;316: 548–549. 17463273 10.1126/science.1140384

[8] National Academies of Sciences E, Medicine. Undergraduate research experiences for STEM students: Successes, challenges, and opportunities: National Academies Press; 2017.

[9] National Academies of Sciences E, and, Medicine. Integrating Discovery-Based Research into the Undergraduate Curriculum: Report of a Convocation. Washington D.C.: National Academies Press; 2015.

[10] Sadler TD, McKinney L. Scientific research for undergraduate students: A review of the literature. J Coll Sci Teach. 2010;39: 43–49.

[11] Spell RM, Guinan JA, Miller KR, Beck CW. Redefining authentic research experiences in introductory biology laboratories and barriers to their implementation. CBE Life Sci Educ. 2014;13: 102–110. doi: 10.1187/cbe.13-08-0169 24591509 PMC3940451

[12] Carter FD, Mandell M, Maton KI. The influence of on-campus, academic year undergraduate research on STEM Ph. D. outcomes: Evidence from the Meyerhoff Scholarship Program. Educ Eval Policy Anal. 2009;31: 441–462. 21785521 10.3102/0162373709348584 PMC3139981

[13] Chang JC. Women and Minorities in the Science, Mathmatics and Engineering Pipeline. ERIC Diges. 2002; Report No. EDO-JC-02-06.

[14] Espinosa L. Pipelines and pathways: Women of color in undergraduate STEM majors and the college experiences that contribute to persistence. Harv Educ Rev. 2011;81: 209–241.

[15] Nagda BA, Gregerman SR, Jonides J, von Hippel W, Lerner JS. Undergraduate student-faculty research partnerships affect student retention. Rev High Ed. 1998;22: 55–72.

[16] Sadler TD, Burgin S, McKinney L, Ponjuan L. Learning science through research apprenticeships: A critical review of the literature. J Res Sci Teach. 2010;47: 235–256.

[17] McDonald KK, Martin AR, Watters CP, Landerholm TE. A faculty development model for transforming a department’s laboratory curriculum with course-based undergraduate research experiences. J Coll Sci Teach. 2019;48: 14–23.

[18] Wei CA, Woodin T. Undergraduate research experiences in biology: alternatives to the apprenticeship model. CBE Life Sci Educ. 2011;10: 123–131. doi: 10.1187/cbe.11-03-0028 21633057 PMC3105915

[19] Bangera G, Brownell SE. Course-based undergraduate research experiences can make scientific research more inclusive. CBE Life Sci Educ. 2014;13: 602–606. doi: 10.1187/cbe.14-06-0099 25452483 PMC4255347

[20] Shapiro C, Moberg-Parker J, Toma S, Ayon C, Zimmerman H, Roth-Johnson EA, et al. Comparing the impact of course-based and apprentice-based research experiences in a life science laboratory curriculum. J Microbiol Biol Educ. 2015;16: 186–197. doi: 10.1128/jmbe.v16i2.1045 26751568 PMC4690559

[21] Corwin LA, Graham MJ, Dolan EL. Modeling course-based undergraduate research experiences: An agenda for future research and evaluation. CBE Life Sci Educ. 2015;14: es1. doi: 10.1187/cbe.14-10-0167 25687826 PMC4353087

[22] Olimpo J, DeChenne-Peters SE, Fisher GR. Development and evaluation of the Tigriopus course-based undergraduate research experience: Impacts on students’ content knowledge, attitudes, and motivation in a majors introductory biology course. CBE Life Sci Educ. 2016;15: ar72. 10.1187/cbe.15-11-0228 PMC5132369 27909022

[23] Roberts R, Hall B, Daubner C, Goodman A, Pikaart M, Sikora A, et al. Flexible implementation of the BASIL CURE. Biochem Mol Biol Educ. 2019;47: 498–505. doi: 10.1002/bmb.21287 31381264

[24] Barral AM, Makhluf H, Soneral P, Gasper B. Small World Initiative: crowdsourcing research of new antibiotics to enhance undergraduate biology teaching (618.41). FASEB J. 2014;28: 618.41.

[25] Brownell SE, Kloser MJ, Fukami T, Shavelson RJ. Undergraduate biology lab courses: Comparing the impactof traditionally based "Cookbook" and authentic research-based courses on student lab experiences. J Coll Sci Teach. 2012;41: 36–45.

[26] Brownell SE, Hekmat-Scafe DS, Singla V, Seawell PC, Imam JFC, Eddy SL, et al. A high-enrollment course-based undergraduate research experience improves student conceptions of scientific thinking and ability to interpret data. CBE Life Sci Educ. 2015;14: ar21. doi: 10.1187/cbe.14-05-0092 26033869 PMC4477737

[27] Winkelmann K, Baloga M, Marcinkowski T, Giannoulis C, Anquandah G, Cohen P. Improving students’ inquiry skills and self-efficacy through research-inspired modules in the general chemistry laboratory. J Chem Educ. 2014;92: 247–255.

[28] Gin LE, Rowland AA, Steinwand B, Bruno J, Corwin LA. Students who fail to achieve predefined research goals may still experience many positive outcomes as a result of CURE Participation. CBE Life Sci Educ. 2018;17: ar57. doi: 10.1187/cbe.18-03-0036 30417757 PMC6755884

[29] Olimpo J, Apodaca J, Hernandez A, Yok-Fong P. Disease and the environment: A health disparities CURE incorporating civic engagement education. Sci Educ Civ Engagem. 2019;11: 13–24.

[30] Martin A, Rechs A, Landerholm T, McDonald K. Course-based undergraduate research experiences spanning two semesters of biology impact student self-efficacy but not future goals. J Coll Sci Teach. 2021;50: 33–47.

[31] Auchincloss LC, Laursen SL, Branchaw JL, Eagan K, Graham M, Hanauer DI, et al. Assessment of course-based undergraduate research experiences: a meeting report. CBE Life Sci Educ. 2014;13: 29–40. doi: 10.1187/cbe.14-01-0004 24591501 PMC3940459

[32] Bur SK, Pomerantz WC, Bade ML, Gee CT. Fragment-based ligand discovery using protein-pbserved 19F NMR: A second semester organic chemistry CURE project. J Chem Educ. 2021;98:1963–73. 10.1021/acs.jchemed.1c00028 PMC10237086 37274366

[33] Khoukhi A. A structured approach to honours undergraduate research course, evaluation rubrics and assessment. J Sci Educ Technol. 2013;22: 630–650.

[34] Thiry H, Weston TJ, Laursen SL, Hunter A-B. The benefits of multi-year research experiences: differences in novice and experienced students’ reported gains from undergraduate research. CBE Life Sc Educ. 2012;11: 260–272. doi: 10.1187/cbe.11-11-0098 22949423 PMC3433299

[35] Hanauer DI, Graham MJ, Betancur L, Bobrownicki A, Cresawn SG, Garlena RA, et al. An inclusive Research Education Community (iREC): Impact of the SEA-PHAGES program on research outcomes and student learning. Proc Natl Acad Sci U S A. 2017;114: 13531–13516. 29208718 10.1073/pnas.1718188115 PMC5754813

[36] Fechheimer M, Webber K, Kleiber PB. How well do undergraduate research programs promote engagement and success of students? CBE Life Sci Educ. 2011;10: 156–163. doi: 10.1187/cbe.10-10-0130 21633064 PMC3105922

[37] Rodenbusch SE, Hernandez PR, Simmons SL, Dolan EL. Early engagement in course-based research increases graduation rates and completion of science, engineering, and mathematics degrees. CBE Life Sci Educ. 2016;15: ar20. doi: 10.1187/cbe.16-03-0117 27252296 PMC4909342

[38] Corwin LA, Runyon CR, Ghanem E, Sandy M, Clark G, Palmer GC, et al. Effects of discovery, iteration, and collaboration in laboratory courses on undergraduates’ research career intentions fully mediated by student ownership. CBE Life Sci Educ. 2018;17: ar20. doi: 10.1187/cbe.17-07-0141 29749845 PMC5998318

[39] Shortlidge EE, Bangera G, Brownell SE. Faculty perspectives on developing and teaching course-based undergraduate research experiences. BioScience. 2016;66: 54–62.

[40] Vora NJ, Vatcheva K, Saldivar MG, Nair S, Lehker MW, Chew SA. Biomedical Freshman Research Initiative: A course-based undergraduate research experience at a Hispanic-Serving Institution. J Lat Educ. 2020: 1–14.

[41] Werby SH, Cegelski L. Design and implementation of a six-session CURE module using biofilms to explore the chemistry–biology interface. J Chem Educ. 2019;96: 2050–2054.

[42] Tootle TL, Hoffmann DS, Allen AK, Spracklen AJ, Groen CM, Kelpsch DJ. Mini-course-based undergraduate research experience. J Coll Sci Teach. 2019;48: 44–54.

[43] Hanauer DI, Nicholes J, Liao F-Y, Beasley A, Henter H. Short-term research experience (SRE) in the traditional lab: Qualitative and quantitative data on outcomes. CBE Life Sci Educ. 2018;17: ar64. doi: 10.1187/cbe.18-03-0046 30496032 PMC6755894

[44] Dahlberg CL, Wiggins BL, Lee SR, Leaf DS, Lily LS, Jordt H, et al. A short, course-based research module provides metacognitive benefits in the form of more sophisticated problem solving. J Coll Sci Teach. 2019;48: 22–30.

[45] Mader CM, Beck CW, Grillo WH, Hollowell GP, Hennington BS, Staub NL, et al. Multi-institutional, multidisciplinary study of the impact of course-based research experiences. J Microbiol Biol Educ. 2017;18: 18.2.44. doi: 10.1128/jmbe.v18i2.1317 28861141 PMC5577972

[46] Shaffer CD, Alvarez CJ, Bednarski AE, Dunbar D, Goodman AL, Reinke C, et al. A course-based research experience: how benefits change with increased investment in instructional time. CBE Life Sci Educ. 2014;13:111–130. doi: 10.1187/cbe-13-08-0152 24591510 PMC3940452

[47] Corwin LA, Dolan EL, Graham MJ, Hanauer DI, Pelaez N. The need to be sure about CUREs: Discovery and relevance as critical elements of CUREs for nonmajors. J Microbiol Biol Educ. 2018;19: 19.3.102. doi: 10.1128/jmbe.v19i3.1683 30377476 PMC6203632

[48] Krim JS, Coté LE, Schwartz RS, Stone EM, Cleeves JJ, Barry KJ, et al. Models and impacts of science research experiences: A review of the literature of CUREs, UREs, and TREs. CBE Life Sci Educ. 2019;18: ar65. doi: 10.1187/cbe.19-03-0069 31782694 PMC6889846

[49] Linn MC, Palmer E, Baranger A, Gerard E, Stone E. Undergraduate research experiences: Impacts and opportunities. Science. 2015;347):1 261757. 10.1126/science.1261757 25657254

[50] Ing M, Burnette JM III, Azzam T, Wessler SR. Participation in a course-based undergraduate research experience results in higher grades in the companion lecture course. Educ Res. 2020: 0013189X20968097.

[51] Shuster MI, Curtiss J, Wright TF, Champion C, Sharifi M, Bosland J. Implementing and evaluating a course-based undergraduate research experience (CURE) at a Hispanic-Serving Institution. Interdiscip J Probl-based Learn. 2019; 13(2).

[52] Shaffer CD, Alvarez C, Bailey C, Barnard D, Bhalla S, Chandrasekaran C, et al. The Genomics Education Partnership: successful integration of research into laboratory classes at a diverse group of undergraduate institutions. CBE Life Sci Educ. 2010;9: 55–69. doi: 10.1187/09-11-0087 20194808 PMC2830162

[53] Jordan TC, Burnett SH, Carson S, Caruso SM, Clase K, DeJong RJ, et al. A broadly implementable research course in phage discovery and genomics for first-year undergraduate students. mBio. 2014;5: e01051–13. doi: 10.1128/mBio.01051-13 24496795 PMC3950523

[54] Bell JK, Eckdahl TT, Hecht DA, Killion PJ, Latzer J, Mans TL, et al. CUREs in biochemistry—where we are and where we should go. Biochem Mol Biol Educ. 2017;45: 7–12. doi: 10.1002/bmb.20989 27357379 PMC5297992

[55] Provost JJ, Bell JK, Bell JE. Development and use of CUREs in biochemistry. Biochemistry Education: From Theory to Practice: ACS Publications; 2019. p. 143–71.

[56] Callahan KP, Mans T, Zhang J, Bell E, Bell JK. Using bioinformatics and molecular visualization to develop student hypotheses in a malate dehydrogenase oriented CURE. CourseSource; 2021.

[57] DeChenne SE, Carew J, Stains M. Published freshman lab exercises as indicators of level of awareness and adoption of instructional practices grounded in discipline-based education research. J of Coll Sci Teach. 2014;43: 60–70.

[58] Corwin LA, Runyon C, Robinson A, Dolan EL. The laboratory course assessment survey: a tool to measure three dimensions of research-course design. CBE Life Sci Educ. 2015;14: ar37. doi: 10.1187/cbe.15-03-0073 26466990 PMC4710398

[59] Gormally C, Brickman P, Lutz M. Developing a Test of Scientific Literacy Skills (TOSLS): measuring undergraduates’ evaluation of scientific information and arguments. CBE Life Sci Educ. 2012;11: 364–377. doi: 10.1187/cbe.12-03-0026 23222832 PMC3516792

[60] Sirum K, Humburg J. The Experimental Design Ability Test (EDAT). Bioscene. 2011;37: 8–16.

[61] Lopatto D, Alvarez C, Barnard D, Chandrasekaran C, Chung J, Du C, et al. Education Forum: Genomics Education Partnership. Science. 2008;322: 684–685. 18974335 10.1126/science.1165351 PMC2953277

[62] Tyler-Wood T, Knezek G, Christensen R. Instruments for assessing interest in STEM content and careers. Journal of Technol and Teach Educ. 2010;18: 345–368.

[63] Glass GV, Peckham PD, Sanders JR. Consequences of failure to meet assumptions underlying the fixed effects analyses of variance and covariance. Rev Educ Res. 1972;42: 237–288.

[64] Lopatto D, Jaworski L. CURE Benchmarks 2015–2018. [cited 2021 Nov 11]. Database available from https://www.grinnell.edu/academics/resources/ctla/assessment/cure-survey

[65] Cooper KM, Blattman JN, Hendrix T, Brownell SE. The impact of broadly relevant novel discoveries on student project ownership in a traditional lab course turned CURE. CBE Life Sci Educ. 2019;18: ar57. 10.1187/cbe.19-06-0113 PMC6829067 31675275

[66] Stains M, Vickrey T. Fidelity of implementation: An overlooked yet critical construct to establish effectiveness of evidence-based instructional practices. CBE Life Sci Educ. 2017;16(1): rm1. doi: 10.1187/cbe.16-03-0113 28213585 PMC5332058

[67] Russell JE, D’Costa AR, Runck C, Barnes DW, Barrera AL, Hurst-Kennedy J, et al. Bridging the undergraduate curriculum using an integrated course-embedded undergraduate research Experience (ICURE). CBE Life Sci Educ. 2015;14: ar4. doi: 10.1187/cbe.14-09-0151 25681416 PMC4353079

[68] Siritunga D, Montero-Rojas M, Carrero K, Toro G, Vélez A, Carrero-Martínez FA. Culturally relevant inquiry-based laboratory module implementations in upper-division genetics and cell biology teaching laboratories. CBE Life Sci Educ. 2011;10: 287–297. doi: 10.1187/cbe.11-04-0035 21885825 PMC3164568

[69] Lipchock JM, Ginther PS, Douglas BB, Bird KE, Loria JP. Exploring protein structure and dynamics through a project‐oriented biochemistry laboratory module. Biochem Mol Biol Educ. 2017;45: 403–410. doi: 10.1002/bmb.21056 28294503

[70] Campbell AM, Eckdahl T, Cronk B, Andresen C, Frederick P, Huckuntod S, et al. pClone: synthetic biology tool makes promoter research accessible to beginning biology students. CBE Life Sci Educ. 2014;13: 285–296. doi: 10.1187/cbe.13-09-0189 26086659 PMC4041505

[71] Wiley EA, Stover NA. Immediate dissemination of student discoveries to a model organism database enhances classroom-based research experiences. CBE Life Sci Educ. 2014;13: 131–138. doi: 10.1187/cbe.13-07-0140 24591511 PMC3940453

[72] Pontrello JK. Bringing research into a first semester organic chemistry laboratory with the multistep synthesis of carbohydrate‐based HIV inhibitor mimics. Biochem Mol Biol Educ. 2015;43: 417–427. doi: 10.1002/bmb.20915 26449849

[73] Pontrello JK. Metalloprotease peptide inhibitors: A semester-long organic synthetic research project for the introductory laboratory course. J Chem Educ. 2015;92: 811–818.

[74] Staub NL, Poxleitner M, Braley A, Smith-Flores H, Pribbenow CM, Jaworski L, et al. Scaling up: adapting a phage-hunting course to increase participation of first-year students in research. CBE Life Sci Educ. 2016;15: ar13. doi: 10.1187/cbe.15-10-0211 27146160 PMC4909335

[75] Sommers AS, Miller AW, Gift AD, Richter-Egger DL, Darr JP, Cutucache CE. CURE disrupted! Takeaways from a CURE without a wet-lab experience. J Chem Educ. 2020;98: 357–367.

[76] Goeden TJ, Kurtz MJ, Quitadamo IJ, Thomas C. Community-based inquiry in allied health biochemistry promotes equity by improving critical thinking for women and showing promise for increasing content gains for ethnic minority students. J Chem Educ. 2015;92: 788–796.

[77] Sandquist E, Cervato C, Ogilvie C. Positive affective and behavioral gains of first-year students in course-based research across disciplines. Scholarsh Pract Undergrad Res. 2019;2: 45.

[78] Flaherty EA, Walker SM, Forrester JH, Ben‐David M. Effects of course‐based undergraduate research experiences (CURE) on wildlife students. Wildl Soc Bull. 2017;41: 701–711.

[79] Rodrigo-Peiris T, Xiang L, Cassone VM. A Low-Intensity, Hybrid design between a “traditional” and a “course-based” research experience yields positive outcomes for science undergraduate freshmen and shows potential for large-scale application. CBE Life Sci Educ. 2018;17: ar53. 10.1187/cbe.17-11-0248 PMC6755889 30335606

[80] Kerr MA, Yan F. Incorporating course-based undergraduate research experiences into analytical chemistry laboratory curricula. J Chem Educ. 2016;93:658–662.

[81] Zhou Y, Jung E, Arroyave R, Radovic M, Shamberger P. Incorporating research experiences into an introductory materials science course. Int J Eng Educ. 2015;31: 1491–1503.

[82] Chang MJ, Sharkness J, Hurtado S, Newman CB. What matters in college for retaining aspiring scientists and engineers from underrepresented racial groups. J Res Sci Teach. 2014;51: 555–580.

[83] Jones MT, Barlow AE, Villarejo M. Importance of undergraduate research for minority persistence and achievement in biology. J Higher Educ. 2010;81: 82–115.

[84] Hallgren KA. Computing inter-rater reliability for observational data: an overview and tutorial. Tutor Quant Methods Psychol. 2012;8: 23–34. doi: 10.20982/tqmp.08.1.p023 22833776 PMC3402032

[85] Boud D, Falchikov N. Quantitative studies of student self-assessment in higher education: A critical analysis of findings. High Educ (Dordr). 1989;18: 529–549.

[86] Falchikov N, Boud D. Student self-assessment in higher education: A meta-analysis. Rev Educ Res. 1989;59: 395–430.

[87] Lopatto D. Survey of undergraduate research experiences (SURE): First findings. Cell Biol Educ. 2004;3: 270–277. doi: 10.1187/cbe.04-07-0045 15592600 PMC533131

[88] Semsar K, Knight JK, Birol G, Smith MK. The Colorado Learning Attitudes about Science Survey (CLASS) for use in biology. CBE Life Sci Educ. 2011;10: 268–78. doi: 10.1187/cbe.10-10-0133 21885823 PMC3164566

[89] Glynn SM, Brickman P, Armstrong N, Taasoobshirazi G. Science motivation questionnaire II: Validation with science majors and non-majors. J Res Sci Teach. 2011;48: 1159–1176.

[90] Hanauer DI, Graham MJ, Hatfull GF. A measure of college student persistence in the sciences (PITS). CBE Life Sci Educ. 2016;15: ar54. doi: 10.1187/cbe.15-09-0185 27810869 PMC5132351

[91] Ebert-May D, Derting JH, Momsen JL, Long TM, Jardeleza SE. What we say is not what we do: Effective evaluation of faculty professional development programs. BioScience. 2011;61: 550–558.

[92] Fast Facts 2011. In: (2011). AAoCC, editor. 2011.

[93] McCook A. Two-year colleges are jumping into the US research pool. Science. 2011;333: 1572–1573. 21921175 10.1126/science.333.6049.1572

[94] Andrews T, Leonard M, Colgrove C, Kalinowski S. Active learning not associated with student learning in a random sample of college biology courses. CBE Life Sci Educ. 2011;10: 394–405. doi: 10.1187/cbe.11-07-0061 22135373 PMC3228657

