# Supplementary material for: Length of course-based undergraduate research experiences (CURE) impacts student learning and attitudinal outcomes: A study of the Malate dehydrogenase CUREs Community (MCC)
Source: PLoS One. 2023 Mar 9;18(3):e0282170. doi: 10.1371/journal.pone.0282170 (PMC9997910; doi:10.1371/journal.pone.0282170)
Supplement: S1 Table — On the post-test the students reported their experiences with three elements of a CURE. Iteration and discovery/relevance were comprised of five items scored 1 (Strongly Disagree) to 7 (Strongly Agree). Collaboration included six items scored using 1 (Never), 2 (One or two times), 3 (Monthly), and 4 (Weekly). (DOCX) [file pone.0282170.s001.docx]

**S1A Table. LCAS by CURE Condition.** On the post-test the students reported their experiences with three elements of a CURE. Iteration and discovery/relevance were comprised of five items scored 1 (Strongly Disagree) to 7 (Strongly Agree). Collaboration included six items scored using 1 (Never), 2 (One or two times), 3 (Monthly), and 4 (Weekly).

| LCAS Subscale | CURE Condition | *n* | Mean | SE | F | η_p_^2^ | Condition  *p*-values & Post-hoc test *p*-values |
| --- | --- | --- | --- | --- | --- | --- | --- |
| Collaboration | Control | 465 | 3.54 | 0.03 | F(2, 1133) = 11.11 | 0.02 | <0.001  cCURE>mCURE, <0.001  cCURE>control, <0.001 |
|  | mCURE | 383 | 3.51 | 0.03 |  |  |  |
|  | cCURE | 288 | 3.70 | 0.03 |  |  |  |
| Discovery/Relevance | Control | 463 | 4.66 | 0.07 | F(2,1131)=141.23 | 0.20 | <0.001  cCURE>mCURE>Control, <0.001 |
|  | mCURE | 384 | 5.69 | 0.06 |  |  |  |
|  | cCURE | 287 | 6.05 | 0.05 |  |  |  |
| Iteration | Control | 462 | 5.04 | 0.06 | F(2, 1130)=16.90 | 0.029 | <0.001  cCURE>mCURE, <0.05  cCURE>Control, <0.001  mCURE>Control, <0.01 |
|  | mCURE | 384 | 5.29 | 0.06 |  |  |  |
|  | cCURE | 287 | 5.55 | 0.07 |  |  |  |

**S1B Table. LCAS, by URM Status and Interaction of Status/Condition.**

| LCAS Subscale | CURE Condition | URM Students | | | White/Asian Students | | | URM Status | | | Interaction of Status/Condition | |
| --- | --- | --- | --- | --- | --- | --- | --- | --- | --- | --- | --- | --- |
|  |  | *n* | Mean | SE | *n* | Mean | SE | F | η_p_^2^ | *p*-value | F | *p*-value |
| Collaboration | Control | 109 | 3.46 | 0.06 | 322 | 3.58 | 0.03 | *F*(1,1045) = 0.73 |  | 0.392 | *F*(2,1045) = 1.28 | 0.278 |
|  | mCURE | 104 | 3.47 | 0.07 | 247 | 3.51 | 0.04 |  |  |  |  |  |
|  | cCURE | 46 | 3.76 | 0.06 | 223 | 3.71 | 0.03 |  |  |  |  |  |
|  | Overall | 259 | 3.52 | 0.04 | 792 | 3.59 | 0.02 |  |  |  |  |  |
| Discovery/  Relevance | Control | 108 | 4.77 | 0.12 | 321 | 4.60 | 0.08 | *F*(1,1045) = 0.21 |  | 0.651 | *F*(2,1045) = 0.70 | 0.496 |
|  | mCURE | 104 | 5.67 | 0.10 | 249 | 5.71 | 0.07 |  |  |  |  |  |
|  | cCURE | 45 | 6.06 | 0.12 | 224 | 6.07 | 0.05 |  |  |  |  |  |
|  | Overall | 794 | 5.36 | 0.05 | 794 | 5.36 | 0.05 |  |  |  |  |  |
| Iteration | Control | 107 | 5.23 | 0.11 | 321 | 4.96 | 0.07 | *F*(1,1044) = 7.76 | 0.007 | 0.005 | *F*(2,1044) = 0.27 | 0.766 |
|  | mCURE | 104 | 5.52 | 0.10 | 249 | 5.18 | 0.08 |  |  |  |  |  |
|  | cCURE | 45 | 5.72 | 0.14 | 224 | 5.56 | 0.08 |  |  |  |  |  |
|  | Overall | 256 | 5.43 | 0.07 | 794 | 5.20 | 0.04 |  |  |  |  |  |
