# Supplementary material for: Length of course-based undergraduate research experiences (CURE) impacts student learning and attitudinal outcomes: A study of the Malate dehydrogenase CUREs Community (MCC)
Source: PLoS One. 2023 Mar 9;18(3):e0282170. doi: 10.1371/journal.pone.0282170 (PMC9997910; doi:10.1371/journal.pone.0282170)
Supplement: S2 Table — On the pretest, students rated their experience with 25 activities that occur in science courses using a scale from 1 = “No experience or feel inexperienced” to 5 = “Extensive experience or mastered this element.” At posttest, students rated their learning gains related to each of the 25 activities using the scale 1 = “No gain or very small gain” to 5 = “Very large gain.” The 15 activities shown in this table were found to be not significantly different by CURE condition for students (Bonferroni correction p < 0.002 is significant). Faculty rated each item by 0 = “not applicable” to 3 = “major”. (DOCX) [file pone.0282170.s002.docx]

**S2A Table. Student and Faculty Learning Activities, Significant.** On the pretest, students rated their experience with 25 activities that occur in science courses using a scale from 1 = “No experience or feel inexperienced” to 5 = “Extensive experience or mastered this element.” At posttest, students rated their learning gains related to each of the 25 activities using the scale 1 = “No gain or very small gain” to 5 = “Very large gain.” The 10 activities shown in this table were found to be significantly different by CURE condition for students (Bonferroni correction *p*<0.002 is significant).

Faculty rated each item by 0 = “not applicable” to 3 = “major”. Statistical tests not run for faculty due to low sample size.

| **Category/Activity** | **CURE**  **Condition** | **Faculty** | | | **Students** | | | | | | | |
| --- | --- | --- | --- | --- | --- | --- | --- | --- | --- | --- | --- | --- |
|  |  | ***n*** | **x̄** | **SE** | ***n*** | **x̄**  **Pre** | **SE Pre** | **x̄**  **Post** | **SE Post** | **F** | **η_p_^2^** | **Condition *p*-values**  **& Post-hoc test *p*-values** |
| Knowledge of Experimental Outcomes: A scripted lab or project in which the students know the expected outcome | cCURE | 12 | 0.75 | 0.22 | 256 | 3.88 | 0.05 | 2.93 | 0.07 | F(2,1064)=17.28 | 0.03 | <0.001  cCURE<mCURE, <0.001  cCURE<control, <0.001 |
|  | mCURE | 11 | 1.36 | 0.28 | 365 | 3.64 | 0.04 | 3.28 | 0.05 |  |  |  |
|  | Control | 13 | 1.46 | 0.29 | 447 | 3.6 | 0.04 | 3.36 | 0.04 |  |  |  |
| Knowledge of Experimental Outcomes: A lab or project in which only the instructor knows the outcome | cCURE | 12 | 1 | 0.21 | 257 | 3.56 | 0.05 | 3.33 | 0.06 | F(2,1052)=7.19 | 0.01 | =0.001  cCURE<mCURE, <0.001  cCURE<control, <0.001 |
|  | mCURE | 11 | 1.45 | 0.31 | 362 | 3.43 | 0.04 | 3.39 | 0.05 |  |  |  |
|  | Control | 13 | 1.54 | 0.31 | 437 | 3.44 | 0.04 | 3.58 | 0.04 |  |  |  |
| Knowledge of Experimental Outcomes: A lab or project where no one knows the outcome | cCURE | 12 | 3 | 0 | 278 | 3.75 | 0.07 | 3.90 | 0.06 | F(2,1035)  =29.47 | 0.05 | <0.001  cCURE>mCURE>control, <0.001 |
|  | mCURE | 11 | 2.45 | 0.28 | 363 | 2.51 | 0.06 | 3.53 | 0.06 |  |  |  |
|  | Control | 13 | 1.31 | 0.35 | 398 | 2.51 | 0.06 | 3.156 | 0.06 |  |  |  |
| Student Involvement in Research Projects: A project in which students have some input into the research process and/or what is being studied | cCURE | 12 | 2.58 | 0.15 | 283 | 3.12 | 0.06 | 4.16 | 0.05 | F(2,1070) =11.86 | 0.02 | <0.001  cCURE<mCURE, =0.014  cCURE<control, <0.001 |
|  | mCURE | 11 | 2.09 | 0.34 | 369 | 2.91 | 0.05 | 3.93 | 0.05 |  |  |  |
|  | Control | 13 | 1.08 | 0.31 | 422 | 2.93 | 0.05 | 3.78 | 0.05 |  |  |  |
| Student Involvement in Research Projects: A project entirely of student design | cCURE | 12 | 1.83 | 0.32 | 276 | 2.45 | 0.07 | 3.84 | 0.07 | F(2,1015)=12.03 | 0.02 | <0.001  cCURE<mCURE, =0.015  cCURE<control, <0.001 |
|  | mCURE | 11 | 1.64 | 0.39 | 349 | 2.32 | 0.06 | 3.56 | 0.07 |  |  |  |
|  | Control | 13 | 0.54 | 0.24 | 394 | 2.39 | 0.06 | 3.37 | 0.06 |  |  |  |
| Student Involvement in Research Projects: Become responsible for part of a project | cCURE | 12 | 2.92 | 0.08 | 287 | 3.91 | 0.054 | 4.22 | 0.05 | F(2,116) =9.06 | 0.02 | <0.001  cCURE<control, <0.001 |
|  | mCURE | 11 | 2.64 | 0.2 | 378 | 3.84 | 0.04 | 4.08 | 0.05 |  |  |  |
|  | Control | 13 | 1.69 | 0.35 | 455 | 3.86 | 0.04 | 3.94 | 0.04 |  |  |  |
| Student Involvement in Research Projects: Read primary scientific literature | cCURE | 12 | 2.75 | 0.13 | 287 | 3.71 | 0.05 | 4.00 | 0.06 | F(2,1096) =7.04 | 0.01 | =0.001  cCURE<mCURE, =0.004  cCURE<control, <0.001 |
|  | mCURE | 11 | 2.18 | 0.18 | 369 | 3.22 | 0.05 | 3.60 | 0.06 |  |  |  |
|  | Control | 13 | 1.31 | 0.29 | 444 | 3.33 | 0.05 | 3.63 | 0.05 |  |  |  |
| Student Involvement in Research Projects: Write a research proposal | cCURE | 12 | 2.83 | 0.11 | 284 | 2.83 | 0.07 | 4.09 | 0.06 | F(2.1016) =26.97 | 0.05 | <0.001  cCURE>mCURE, <0.001  cCURE>control, <0.001  mCURE>control, =0.018 |
|  | mCURE | 11 | 2.09 | 0.31 | 355 | 2.54 | 0.06 | 3.63 | 0.06 |  |  |  |
|  | Control | 13 | 0.62 | 0.24 | 281 | 2.54 | 0.06 | 3.40 | 0.06 |  |  |  |
| Presenting Results: Present results orally | cCURE | 12 | 2.75 | 0.13 | 281 | 3.27 | 0.06 | 4.19 | 0.06 | F(2.1087)=15.17 | 0.03 | <0.001  cCURE<mCURE, <0.001  cCURE<control, <0.001 |
|  | mCURE | 11 | 2.45 | 0.21 | 370 | 3.02 | 0.06 | 3.81 | 0.06 |  |  |  |
|  | Control | 13 | 1.77 | 0.32 | 440 | 3.10 | 0.05 | 3.72 | 0.05 |  |  |  |
| Present Results: Present posters | cCURE | 12 | 1.42 | 0.42 | 281 | 3.27 | 0.06 | 4.19 | 0.06 | F(2,1087) =15.17 | 0.03 | <0.001  cCURE<mCURE, <0.001  cCURE<control, <0.001 |
|  | mCURE | 11 | 1.73 | 0.43 | 370 | 3.58 | 0.05 | 3.88 | 0.06 |  |  |  |
|  | Control | 13 | 1 | 0.32 | 448 | 3.71 | 0.04 | 3.85 | 0.05 |  |  |  |

**S2B Table. Student and Faculty Learning Activities, Non-Significant.** On the pretest, students rated their experience with 25 activities that occur in science courses using a scale from 1 = “No experience or feel inexperienced” to 5 = “Extensive experience or mastered this element.” At posttest, students rated their learning gains related to each of the 25 activities using the scale 1 = “No gain or very small gain” to 5 = “Very large gain.” The 15 activities shown in this table were found to be not significantly different by CURE condition for students (Bonferroni correction *p*<0.002 is significant).

Faculty rated each item by 0 = “not applicable” to 3 = “major”. Statistical tests not run for faculty due to low sample size.

| **Category/Activity** | **CURE**  **Condition** | **Faculty** | | | **Student** | | | | | | |
| --- | --- | --- | --- | --- | --- | --- | --- | --- | --- | --- | --- |
|  |  | ***n*** | **x̄** | **SE** | ***n*** | **x̄**  **Pre** | **SE Pre** | **x̄**  **Post** | **SE Post** | **Condition** | |
|  |  |  |  |  |  |  |  |  |  | **F** | ***p*** |
| Involvement in Research Process: At least one project that is assigned and  structured by the instructor | Control | 13 | 2.62 | 0.18 | 448 | 3.66 | 0.05 | 3.73 | 0.05 | F(2,1094) =0.75 | 0.471 |
|  | mCURE | 11 | 2.00 | 0.27 | 374 | 3.71 | 0.05 | 3.66 | 0.05 |  |  |
|  | cCURE | 12 | 1.33 | 0.26 | 276 | 3.89 | 0.04 | 3.70 | 0.04 |  |  |
| Involvement in Research Process: Critique the work of other students | Control | 13 | 0.69 | 0.26 | 410 | 2.97 | 0.05 | 3.39 | 0.06 | F(2,1028) =0.21 | 0.809 |
|  | mCURE | 11 | 1.73 | 0.36 | 350 | 2.90 | 0.06 | 3.34 | 0.06 |  |  |
|  | cCURE | 12 | 1.25 | 0.22 | 272 | 3.09 | 0.06 | 3.43 | 0.07 |  |  |
| Presenting Results: Present results in written papers or reports | Control | 13 | 2.77 | 0.12 | 448 | 3.71 | 0.04 | 3.85 | 0.05 | F(2,1097) =2.01 | 0.134 |
|  | mCURE | 11 | 2.82 | 0.12 | 370 | 3.58 | 0.05 | 3.88 | 0.06 |  |  |
|  | cCURE | 12 | 2.83 | 0.11 | 283 | 3.69 | 0.05 | 4.00 | 0.06 |  |  |
| Course Structure: Work individually | Control | 13 | 1.15 | 0.34 | 441 | 3.63 | 0.05 | 3.27 | 0.05 | F(2,1061) =0.54 | 0.585 |
|  | mCURE | 11 | 1.09 | 0.31 | 349 | 3.56 | 0.06 | 3.18 | 0.06 |  |  |
|  | cCURE | 12 | 0.67 | 0.19 | 275 | 3.59 | 0.06 | 3.22 | 0.07 |  |  |
| Course Structure: Work as a whole class | Control | 13 | 1.15 | 0.32 | 433 | 3.11 | 0.05 | 3.24 | 0.05 | F(2,1060) =0.83 | 0.435 |
|  | mCURE | 11 | 1.55 | 0.34 | 355 | 3.12 | 0.05 | 3.18 | 0.06 |  |  |
|  | cCURE | 12 | 1.25 | 0.18 | 276 | 3.09 | 0.06 | 3.12 | 0.07 |  |  |
| Course Structure: Work in small groups | Control | 13 | 2.46 | 0.24 | 457 | 3.93 | 0.04 | 3.95 | 0.04 | F(2,119) =3.39 | 0.034 |
|  | mCURE | 11 | 2.82 | 0.12 | 379 | 3.98 | 0.04 | 4.04 | 0.05 |  |  |
|  | cCURE | 12 | 2.92 | 0.08 | 287 | 3.97 | 0.04 | 4.14 | 0.05 |  |  |
| Course Structure: Listen to lectures | Control | 13 | 2.00 | 0.23 | 452 | 4.24 | 0.04 | 3.67 | 0.05 | F(2,1103) =1.25 | 0.288 |
|  | mCURE | 11 | 1.91 | 0.21 | 373 | 4.16 | 0.05 | 3.60 | 0.06 |  |  |
|  | cCURE | 12 | 1.33 | 0.19 | 282 | 4.33 | 0.04 | 3.55 | 0.06 |  |  |
| Course Structure Read a textbook | Control | 13 | 1.15 | 0.32 | 415 | 4.05 | 0.04 | 3.18 | 0.06 | F(2,1001) =6.23 | 0.002 |
|  | mCURE | 11 | 0.91 | 0.37 | 336 | 4.01 | 0.05 | 3.11 | 0.07 |  |  |
|  | cCURE | 12 | 0.25 | 0.13 | 254 | 4.20 | 0.05 | 2.85 | 0.07 |  |  |
| Course Structure Work on problem sets | Control | 13 | 1.46 | 0.29 | 437 | 4.03 | 0.04 | 3.57 | 0.05 | F(2,1037) =6.40 | 0.002 |
|  | mCURE | 11 | 1.64 | 0.34 | 349 | 3.99 | 0.04 | 3.49 | 0.06 |  |  |
|  | cCURE | 12 | 0.75 | 0.18 | 255 | 4.17 | 0.05 | 3.25 | 0.07 |  |  |
| Course Structure Take tests in class | Control | 13 | 1.62 | 0.31 | 431 | 4.26 | 0.04 | 3.24 | 0.06 | F(2,1055) =3.48 | 0.031 |
|  | mCURE | 11 | 1.73 | 0.33 | 355 | 4.27 | 0.04 | 3.36 | 0.06 |  |  |
|  | cCURE | 12 | 0.25 | 0.13 | 273 | 4.33 | 0.04 | 3.12 | 0.07 |  |  |
| Course Structure Discuss reading materials in class | Control | 13 | 0.77 | 0.20 | 425 | 3.92 | 0.04 | 3.63 | 0.05 | F(2,1037) =1.48 | 0.229 |
|  | mCURE | 11 | 1.27 | 0.33 | 341 | 3.88 | 0.05 | 3.52 | 0.06 |  |  |
|  | cCURE | 12 | 1.50 | 0.19 | 275 | 4.14 | 0.04 | 3.69 | 0.06 |  |  |
| Data Handling: Collect data | Control | 13 | 2.85 | 0.10 | 457 | 3.79 | 0.04 | 4.05 | 0.04 | F(2,1110) =6.24 | 0.002 |
|  | mCURE | 11 | 2.91 | 0.09 | 374 | 3.84 | 0.05 | 4.09 | 0.05 |  |  |
|  | cCURE | 12 | 3.00 | 0.00 | 283 | 3.95 | 0.05 | 4.29 | 0.05 |  |  |
| Data Handling: Analyze data | Control | 13 | 2.69 | 0.13 | 457 | 3.73 | 0.04 | 4.18 | 0.04 | F(2.1116) =3.00 | 0.050 |
|  | mCURE | 11 | 2.82 | 0.12 | 378 | 3.78 | 0.04 | 4.20 | 0.05 |  |  |
|  | cCURE | 12 | 3.00 | 0.00 | 285 | 3.84 | 0.05 | 4.34 | 0.05 |  |  |
| Data Handling: Maintain a lab notebook | Control | 13 | 2.38 | 0.24 | 438 | 3.99 | 0.04 | 3.68 | 0.05 | F(2,1072) =5.18 | 0.006 |
|  | mCURE | 11 | 2.45 | 0.25 | 356 | 3.88 | 0.05 | 3.53 | 0.07 |  |  |
|  | cCURE | 12 | 3.00 | 0.00 | 282 | 4.03 | 0.04 | 3.84 | 0.06 |  |  |
| Data Handling: Computer modeling | Control | 13 | 1.15 | 0.25 | 393 | 2.44 | 0.06 | 3.22 | 0.06 | F(2,999) =5.56 | 0.004 |
|  | mCURE | 11 | 1.82 | 0.35 | 348 | 2.40 | 0.06 | 3.34 | 0.07 |  |  |
|  | cCURE | 12 | 1.50 | 0.26 | 262 | 2.43 | 0.06 | 3.53 | 0.07 |  |  |
