# Supplementary material for: Length of course-based undergraduate research experiences (CURE) impacts student learning and attitudinal outcomes: A study of the Malate dehydrogenase CUREs Community (MCC)
Source: PLoS One. 2023 Mar 9;18(3):e0282170. doi: 10.1371/journal.pone.0282170 (PMC9997910; doi:10.1371/journal.pone.0282170)
Supplement: S3 Table — On the pretest, students rated their experience with 25 activities that occur in science courses using a scale from 1 = “No experience or feel inexperienced” to 5 = “Extensive experience or mastered this element.” At posttest, students rated their learning gains related to each of the 25 activities using the scale 1 = “No gain or very small gain” to 5 = “Very large gain.” Bonferroni correction p < 0.002 is significant. (DOCX) [file pone.0282170.s003.docx]

**S3 Table. Student Learning Activities by URM Status.** On the pretest, students rated their experience with 25 activities that occur in science courses using a scale from 1 = “No experience or feel inexperienced” to 5 = “Extensive experience or mastered this element.” At posttest, students rated their learning gains related to each of the 25 activities using the scale 1 = “No gain or very small gain” to 5 = “Very large gain.” Bonferroni correction *p* < 0.002 is significant.

| Category/Activity | CURE  Condition | URM Students | | | White/Asian Students | | |
| --- | --- | --- | --- | --- | --- | --- | --- |
|  |  | *n* | x̄ Pre | x̄ Post | *n* | x̄ Pre | x̄ Post |
| Knowing Outcome: A scripted lab or project in which the students know the expected outcome. | Control | 106 | 3.44 | 3.52 | 310 | 3.65 | 3.30 |
|  | mCURE | 99 | 3.49 | 3.27 | 237 | 3.72 | 3.26 |
|  | cCURE | 38 | 3.55 | 3.16 | 200 | 3.97 | 2.88 |
|  | Overall | 243 | 3.48 | 3.36 | 747 | 3.76 | 3.18 |
|  |  | | | F | | *p* | |
|  | Effect of URM Status | | | F(2,983) = 5.58 | | 0.018 | |
|  | Interaction of Status/Condition | | | F(1,983) = 1.19 | | 0.304 | |
| Knowing Outcome: A lab or project in which only the instructor knows the outcome. | CURE  Condition | URM Students | | | White/Asian Students | | |
|  |  | *n* | x̄ Pre | x̄ Post | *n* | x̄ Pre | x̄ Post |
|  | Control | 104 | 3.38 | 3.77 | 304 | 3.48 | 3.51 |
|  | mCURE | 96 | 3.54 | 3.61 | 237 | 3.42 | 3.31 |
|  | cCURE | 40 | 3.40 | 3.45 | 199 | 3.61 | 3.29 |
|  | Overall | 240 | 3.45 | 3.65 | 740 | 3.50 | 3.39 |
|  |  | | | F | | *p* | |
|  | Effect of URM Status | | | F(2,973) = 10.78 | | 0.001, URM>non | |
|  | Interaction of Status/Condition | | | F(1,973) = 0.20 | | 0.822 | |
| Knowing Outcome: A lab or project where no one knows the outcome. | CURE  Condition | URM Students | | | White/Asian Students | | |
|  |  | *n* | x̄ Pre | x̄ Post | *n* | x̄ Pre | x̄ Post |
|  | Control | 97 | 2.55 | 3.42 | 274 | 2.52 | 3.08 |
|  | mCURE | 99 | 2.47 | 3.65 | 236 | 2.50 | 3.47 |
|  | cCURE | 45 | 2.58 | 4.18 | 215 | 2.81 | 3.87 |
|  | Overall | 241 | 2.52 | 3.66 | 725 | 2.60 | 3.44 |
|  |  | | | F | | *p* | |
|  | Effect of URM Status | | | F(2,959) = 9.36 | | 0.002 | |
|  | Interaction of Status/Condition | | | F(1,959) = 0.39 | | 0.676 | |
| Involvement in Research Process: At least one project that is assigned and structured by the instructor. | CURE  Condition | URM Students | | | White/Asian Students | | |
|  |  | *n* | x̄ Pre | x̄ Post | *n* | x̄ Pre | x̄ Post |
|  | Control | 105 | 3.45 | 3.71 | 312 | 3.75 | 3.75 |
|  | mCURE | 99 | 3.81 | 3.84 | 245 | 3.70 | 3.59 |
|  | cCURE | 44 | 3.59 | 4.05 | 214 | 3.99 | 3.61 |
|  | Overall | 248 | 3.62 | 3.82 | 771 | 3.80 | 3.66 |
|  |  | | | F | | *p* | |
|  | Effect of URM Status | | | F(2,1012) = 10.83 | | 0.001, URM>non | |
|  | Interaction of Status/Condition | | | F(1,1012) = 3.84 | | 0.022 | |
| Involvement in Research Process: A project in which the students have some input into the research process and/or what is being | CURE  Condition | URM Students | | | White/Asian Students | | |
|  |  | *n* | x̄ Pre | x̄ Post | *n* | x̄ Pre | x̄ Post |
|  | Control | 102 | 2.78 | 3.81 | 289 | 3.00 | 3.75 |
|  | mCURE | 99 | 2.92 | 4.10 | 240 | 2.89 | 3.84 |
|  | cCURE | 43 | 2.87 | 4.30 | 219 | 3.18 | 4.12 |
|  | Overall | 247 | 2.85 | 4.02 | 748 | 3.02 | 3.89 |
|  |  | | | F | | *p* | |

| studied. | Effect of URM Status | | | F(2,988) = 6.17 | | 0.013 | |
| --- | --- | --- | --- | --- | --- | --- | --- |
|  | Interaction of Status/Condition | | | F(1,988) = 0.67 | | 0.514 | |
| Involvement in Research Process: A project entirely of student design. | CURE  Condition | URM Students | | | White/Asian Students | | |
|  |  | *n* | x̄ Pre | x̄ Post | *n* | x̄ Pre | x̄ Post |
|  | Control | 101 | 2.23 | 3.57 | 262 | 2.45 | 3.25 |
|  | mCURE | 95 | 2.37 | 3.64 | 227 | 2.27 | 3.49 |
|  | cCURE | 43 | 2.23 | 3.98 | 215 | 2.52 | 3.83 |
|  | Overall | 239 | 2.28 | 3.67 | 704 | 2.41 | 3.51 |
|  |  | | | F | | *p* | |
|  | Effect of URM Status | | | F(2,936) = 5.21 | | 0.023 | |
|  | Interaction of Status/Condition | | | F(1,936) = 0.54 | | 0.586 | |
| Involvement in Research Process: Become responsible for a part of the project. | CURE  Condition | URM Students | | | White/Asian Students | | |
|  |  | *n* | x̄ Pre | x̄ Post | *n* | x̄ Pre | x̄ Post |
|  | Control | 108 | 3.74 | 3.94 | 315 | 3.91 | 3.91 |
|  | mCURE | 102 | 3.83 | 4.17 | 246 | 3.85 | 4.03 |
|  | cCURE | 47 | 3.81 | 4.32 | 222 | 3.95 | 4.21 |
|  | Overall | 257 | 3.79 | 4.10 | 783 | 3.90 | 4.03 |
|  |  | | | F | | *p* | |
|  | Effect of URM Status | | | F(2,1033) = 2.46 | | 0.117 | |
|  | Interaction of Status/Condition | | | F(1,1033) = 0.19 | | 0.826 | |
| Involvement in Research Process: Read primary scientific literature. | CURE  Condition | URM Students | | | White/Asian Students | | |
|  |  | *n* | x̄ Pre | x̄ Post | *n* | x̄ Pre | x̄ Post |
|  | Control | 105 | 3.10 | 3.76 | 307 | 3.38 | 3.55 |
|  | mCURE | 97 | 3.13 | 3.76 | 242 | 3.27 | 3.52 |
|  | cCURE | 47 | 3.47 | 4.09 | 222 | 3.81 | 3.99 |
|  | Overall | 249 | 3.18 | 3.82 | 771 | 3.47 | 3.67 |
|  |  | | | F | | *p* | |
|  | Effect of URM Status | | | F(2,1013) = 9.24 | | 0.002 | |
|  | Interaction of Status/Condition | | | F(1,1013) = 0.12 | | 0.885 | |
| Involvement in Research Process: Write a research proposal. | CURE  Condition | URM Students | | | White/Asian Students | | |
|  |  | *n* | x̄ Pre | x̄ Post | *n* | x̄ Pre | x̄ Post |
|  | Control | 94 | 2.48 | 3.61 | 256 | 2.53 | 3.29 |
|  | mCURE | 96 | 2.59 | 3.81 | 230 | 2.51 | 3.53 |
|  | cCURE | 47 | 2.45 | 4.17 | 219 | 2.95 | 4.07 |
|  | Overall | 237 | 2.52 | 3.80 | 705 | 2.66 | 3.61 |
|  |  | | | F | | *p* | |
|  | Effect of URM Status | | | F(2,935) = 8.35 | | 0.004 | |
|  | Interaction of Status/Condition | | | F(1,935) = 0.24 | | 0.790 | |
| Involvement in Research Process: Critique the work of other students. | CURE  Condition | URM Students | | | White/Asian Students | | |
|  |  | *n* | x̄ Pre | x̄ Post | *n* | x̄ Pre | x̄ Post |
|  | Control | 100 | 2.90 | 3.50 | 280 | 2.98 | 3.36 |
|  | mCURE | 93 | 3.04 | 3.53 | 230 | 2.81 | 3.24 |
|  | cCURE | 46 | 3.11 | 3.59 | 208 | 3.10 | 3.43 |
|  | Overall | 239 | 3.00 | 3.53 | 718 | 2.96 | 3.34 |
|  |  | | | F | | *p* | |
|  | Effect of URM Status | | | F(2,950) = 4.24 | | 0.040 | |
|  | Interaction of Status/Condition | | | F(1,950) = 0.14 | | 0.874 | |
| Presenting Results: | CURE  Condition | URM Students | | | White/Asian Students | | |
|  |  | *n* | x̄ Pre | x̄ Post | *n* | x̄ Pre | x̄ Post |

| Present results orally. | Control | 105 | 2.95 | 3.77 | 303 | 3.15 | 3.69 |
| --- | --- | --- | --- | --- | --- | --- | --- |
|  | mCURE | 98 | 3.01 | 3.99 | 243 | 3.01 | 3.74 |
|  | cCURE | 46 | 3.20 | 4.22 | 217 | 3.34 | 4.19 |
|  | Overall | 249 | 3.02 | 3.94 | 763 | 3.16 | 3.85 |
|  |  | | | F | | *p* | |
|  | Effect of URM Status | | | F(2,1005) = 3.08 | | 0.096 | |
|  | Interaction of Status/Condition | | | F(1,1005) = 0.51 | | 0.601 | |
| Presenting Results: Present results in written papers or reports | CURE  Condition | URM Students | | | White/Asian Students | | |
|  |  | *n* | x̄ Pre | x̄ Post | *n* | x̄ Pre | x̄ Post |
|  | Control | 108 | 3.70 | 4.01 | 308 | 3.71 | 3.78 |
|  | mCURE | 97 | 3.63 | 3.98 | 243 | 3.56 | 3.81 |
|  | cCURE | 47 | 3.51 | 4.11 | 218 | 3.78 | 3.99 |
|  | Overall | 252 | 3.64 | 4.02 | 769 | 3.68 | 3.85 |
|  |  | | | F | | *p* | |
|  | Effect of URM Status | | | F(2,1014) = 5.21 | | 0.023 | |
|  | Interaction of Status/Condition | | | F(1,1014) = 0.12 | | 0.888 | |
| Presenting Results: Present posters. | CURE  Condition | URM Students | | | White/Asian Students | | |
|  |  | *n* | x̄ Pre | x̄ Post | *n* | x̄ Pre | x̄ Post |
|  | Control | 95 | 2.92 | 3.48 | 261 | 2.79 | 3.35 |
|  | mCURE | 89 | 3.04 | 3.48 | 198 | 2.82 | 3.20 |
|  | cCURE | 39 | 2.74 | 9.85 | 188 | 3.09 | 3.64 |
|  | Overall | 223 | 2.94 | 3.55 | 674 | 2.89 | 3.39 |
|  |  | | | F | | *p* | |
|  | Effect of URM Status | | | F(2,863) = 3.86 | | 0.050 | |
|  | Interaction of Status/Condition | | | F(1,863) = 0.22 | | 0.801 | |
| Course Structure: Work individually. | CURE  Condition | URM Students | | | White/Asian Students | | |
|  |  | *n* | x̄ Pre | x̄ Post | *n* | x̄ Pre | x̄ Post |
|  | Control | 107 | 3.64 | 3.61 | 302 | 3.64 | 3.14 |
|  | mCURE | 94 | 3.78 | 3.19 | 226 | 3.46 | 3.18 |
|  | cCURE | 42 | 3.45 | 3.26 | 215 | 3.67 | 3.22 |
|  | Overall | 243 | 3.66 | 3.39 | 743 | 3.59 | 3.17 |
|  |  | | | F | | *p* | |
|  | Effect of URM Status | | | F(2,979) = 3.88 | | 0.049 | |
|  | Interaction of Status/Condition | | | F(1,979) = 4.03 | | 0.018 | |
| Course Structure: Work as a whole class. | CURE  Condition | URM Students | | | White/Asian Students | | |
|  |  | *n* | x̄ Pre | x̄ Post | *n* | x̄ Pre | x̄ Post |
|  | Control | 105 | 3.15 | 3.53 | 296 | 3.08 | 3.15 |
|  | mCURE | 95 | 3.17 | 3.35 | 230 | 3.12 | 3.06 |
|  | cCURE | 43 | 3.19 | 3.21 | 215 | 3.12 | 3.16 |
|  | Overall | 243 | 3.16 | 3.40 | 741 | 3.10 | 3.12 |
|  |  | | | F | | *p* | |
|  | Effect of URM Status | | | F(2,977) = 7.46 | | 0.006 | |
|  | Interaction of Status/Condition | | | F(1,977) = 1.24 | | 0.291 | |
| Course Structure: Work in small groups. | CURE  Condition | URM Students | | | White/Asian Students | | |
|  |  | *n* | x̄ Pre | x̄ Post | *n* | x̄ Pre | x̄ Post |
|  | Control | 108 | 3.85 | 3.98 | 317 | 3.96 | 3.91 |
|  | mCURE | 103 | 4.02 | 4.16 | 246 | 3.98 | 3.97 |
|  | cCURE | 47 | 3.98 | 4.13 | 222 | 4.00 | 4.13 |
|  | Overall | 258 | 3.94 | 4.08 | 785 | 3.98 | 3.99 |

|  |  | | | F | | *p* | |
| --- | --- | --- | --- | --- | --- | --- | --- |
|  | Effect of URM Status | | | F(2,1036) = 1.69 | | 0.194 | |
|  | Interaction of Status/Condition | | | F(1,1036) = 0.49 | | 0.612 | |
| Course Structure: Listen to lectures. | CURE  Condition | URM Students | | | White/Asian Students | | |
|  |  | *n* | x̄ Pre | x̄ Post | *n* | x̄ Pre | x̄ Post |
|  | Control | 106 | 4.15 | 4.07 | 314 | 4.27 | 3.52 |
|  | mCURE | 102 | 4.20 | 3.74 | 241 | 4.15 | 3.49 |
|  | cCURE | 47 | 4.30 | 3.66 | 217 | 4.35 | 3.48 |
|  | Overall | 255 | 4.20 | 3.86 | 772 | 4.26 | 3.50 |
|  |  | | | F | | *p* | |
|  | Effect of URM Status | | | F2(2,1020) = 16.98 | | <0.001, URM>non | |
|  | Interaction of Status/Condition | | | F2(1,1020) = 2.34 | | 0.097 | |
| Course Structure: Read a textbook. | CURE  Condition | URM Students | | | White/Asian Students | | |
|  |  | *n* | x̄ Pre | x̄ Post | *n* | x̄ Pre | x̄ Post |
|  | Control | 105 | 4.01 | 3.70 | 279 | 4.05 | 2.99 |
|  | mCURE | 98 | 3.98 | 3.43 | 209 | 4.01 | 2.89 |
|  | cCURE | 41 | 4.27 | 2.90 | 196 | 4.20 | 2.83 |
|  | Overall | 244 | 4.04 | 3.46 | 684 | 4.08 | 2.92 |
|  |  | | | F(2,951) | | *p* | |
|  | Effect of URM Status | | | F(2,951) = 16.98 | | <0.001, URM>non | |
|  | Interaction of Status/Condition | | | F(1,951) = 3.52 | | 0.030 | |
| Course Structure: Work on problem sets. | CURE  Condition | URM Students | | | White/Asian Students | | |
|  |  | *n* | x̄ Pre | x̄ Post | *n* | x̄ Pre | x̄ Post |
|  | Control | 105 | 3.97 | 4.00 | 300 | 4.04 | 3.41 |
|  | mCURE | 99 | 3.97 | 3.87 | 220 | 4.03 | 3.30 |
|  | cCURE | 42 | 4.17 | 3.67 | 196 | 4.20 | 3.15 |
|  | Overall | 246 | 4.00 | 3.89 | 716 | 4.08 | 3.30 |
|  |  | | | F | | *p* | |
|  | Effect of URM Status | | | F(2,955) = 38.67 | | <0.001, URM>non | |
|  | Interaction of Status/Condition | | | F(1,955) = 0.05 | | 0.948 | |
| Course Structure: Take tests in class. | CURE  Condition | URM Students | | | White/Asian Students | | |
|  |  | *n* | x̄ Pre | x̄ Post | *n* | x̄ Pre | x̄ Post |
|  | Control | 104 | 4.31 | 3.67 | 296 | 4.26 | 3.06 |
|  | mCURE | 101 | 4.32 | 3.50 | 226 | 4.28 | 3.23 |
|  | cCURE | 43 | 4.37 | 3.42 | 212 | 4.35 | 3.08 |
|  | Overall | 248 | 4.32 | 3.56 | 734 | 4.29 | 3.12 |
|  |  | | | F | | *p* | |
|  | Effect of URM Status | | | F(2,975) = 19.96 | | <0.001, URM>non | |
|  | Interaction of Status/Condition | | | F(1,975) = 1.84 | | 0.159 | |
| Course Structure: Discuss reading materials in class. | CURE  Condition | URM Students | | | White/Asian Students | | |
|  |  | *n* | x̄ Pre | x̄ Post | *n* | x̄ Pre | x̄ Post |
|  | Control | 104 | 3.94 | 4.00 | 291 | 3.92 | 3.47 |
|  | mCURE | 96 | 3.86 | 3.75 | 215 | 3.89 | 3.39 |
|  | cCURE | 45 | 4.11 | 3.71 | 213 | 4.18 | 3.69 |
|  | Overall | 245 | 3.94 | 3.85 | 719 | 3.99 | 3.51 |
|  |  | | | F | | *p* | |
|  | Effect of URM Status | | | F(2,957) = 12.22 | | <0.001, URM>non | |
|  | Interaction of Status/Condition | | | F(1,957) = 2.56 | | 0.078 | |
|  |  | URM Students | | | White/Asian Students | | |

| Data Handling: Collect data. | CURE  Condition | *n* | x̄ Pre | x̄ Post | *n* | x̄ Pre | x̄ Post |
| --- | --- | --- | --- | --- | --- | --- | --- |
|  | Control | 107 | 3.67 | 4.08 | 318 | 3.84 | 4.01 |
|  | mCURE | 102 | 3.82 | 4.14 | 243 | 3.84 | 4.08 |
|  | cCURE | 45 | 3.84 | 4.42 | 220 | 4.02 | 4.27 |
|  | Overall | 254 | 3.77 | 4.17 | 781 | 3.89 | 4.11 |
|  |  | | | F | | *p* | |
|  | Effect of URM Status | | | F(2,1028) = 2.70 | | 0.101 | |
|  | Interaction of Status/Condition | | | F(1,1028) = 0.21 | | 0.807 | |
| Data Handling: Analyze data. | CURE  Condition | URM Students | | | White/Asian Students | | |
|  |  | *n* | x̄ Pre | x̄ Post | *n* | x̄ Pre | x̄ Post |
|  | Control | 108 | 3.63 | 4.26 | 317 | 3.78 | 4.15 |
|  | mCURE | 103 | 3.83 | 4.25 | 245 | 3.76 | 4.18 |
|  | cCURE | 47 | 3.77 | 4.47 | 220 | 3.90 | 4.31 |
|  | Overall | 258 | 3.73 | 4.29 | 782 | 3.81 | 4.21 |
|  |  | | | F | | *p* | |
|  | Effect of URM Status | | | F(2,1033) = 3.89 | | 0.049 | |
|  | Interaction of Status/Condition | | | F(1,1033) = 0.23 | | 0.796 | |
| Data Handling: Maintain a lab notebook. | CURE  Condition | URM Students | | | White/Asian Students | | |
|  |  | *n* | x̄ Pre | x̄ Post | *n* | x̄ Pre | x̄ Post |
|  | Control | 106 | 4.07 | 4.15 | 302 | 3.96 | 3.49 |
|  | mCURE | 98 | 3.92 | 3.74 | 228 | 3.89 | 3.39 |
|  | cCURE | 47 | 4.09 | 3.85 | 218 | 4.03 | 3.84 |
|  | Overall | 251 | 4.01 | 3.94 | 748 | 3.96 | 3.56 |
|  |  | | | F | | *p* | |
|  | Effect of URM Status | | | F(2,992) = 14.99 | | <0.001, URM>non | |
|  | Interaction of Status/Condition | | | F(1,992) = 4.49 | | 0.011 | |
| Data Handling: Computer modeling. | CURE  Condition | URM Students | | | White/Asian Students | | |
|  |  | *n* | x̄ Pre | x̄ Post | *n* | x̄ Pre | x̄ Post |
|  | Control | 98 | 2.71 | 3.66 | 266 | 2.31 | 3.03 |
|  | mCURE | 96 | 2.51 | 3.49 | 223 | 2.30 | 3.25 |
|  | cCURE | 45 | 2.40 | 3.73 | 201 | 2.46 | 3.48 |
|  | Overall | 239 | 2.57 | 3.61 | 690 | 2.35 | 3.23 |
|  |  | | | F(2,922) | | *p* | |
|  | Effect of URM Status | | | F(2,922) = 12.79 | | <0.001, URM>non | |
|  | Interaction of Status/Condition | | | F(1,922) = 1.63 | | 0.197 | |
