# Supplementary material for: Length of course-based undergraduate research experiences (CURE) impacts student learning and attitudinal outcomes: A study of the Malate dehydrogenase CUREs Community (MCC)
Source: PLoS One. 2023 Mar 9;18(3):e0282170. doi: 10.1371/journal.pone.0282170 (PMC9997910; doi:10.1371/journal.pone.0282170)
Supplement: S4 Table — Table A: CURE post learning gains by CURE condition. Table B: CURE learning gains by URM status and interaction of status/condition. Table C: CURE individual post learning gains (Bonferroni correction for 21 statements, p<0.002). (DOCX) [file pone.0282170.s004.docx]

**S4 Table. CURE Learning Gains.** Table A: CURE Post Learning Gains by CURE condition. Table B: CURE Learning Gains by URM Status and Interaction of Status/Condition. Table C: CURE Individual Post Learning Gains (Bonferroni correction for 21 statements, *p*<0.02).

Table A: CURE Post Learning Gains by CURE condition.

| CURE  Condition | *n* | Mean | SE | Condition | |
| --- | --- | --- | --- | --- | --- |
|  |  |  |  | F | *p* |
| Control | 457 | 3.58 | 0.04 | F(2,1120) = 1.20 | 0.302 |
| mCURE | 379 | 3.57 | 0.05 |  |  |
| cCURE | 287 | 3.67 | 0.05 |  |  |

Table B: CURE Post Learning Gains by URM Status and Interaction of Status/Condition.

| CURE  Condition | URM Students | | | White/Asian Students | | | URM Status | | Interaction of  Status/Condition | |
| --- | --- | --- | --- | --- | --- | --- | --- | --- | --- | --- |
|  | *n* | Mean | SE | *n* | Mean | SE | F | *p* | F | *p* |
| Control | 108 | 3.87 | 0.07 | 317 | 3.47 | 0.05 | F(1,1037) = 26.23 | <0.001 URM>non | F(2,1037) = 0.51 | 0.602 |
| mCURE | 103 | 3.77 | 0.09 | 246 | 3.47 | 0.06 |  |  |  |  |
| cCURE | 47 | 3.88 | 0.11 | 222 | 3.62 | 0.06 |  |  |  |  |
| Overall | 258 | 3.83 | 0.05 | 785 | 3.51 | 0.06 |  |  |  |  |

Table C: CURE individual post learning gains (Bonferroni correction for 21 statements, *p*<0.002)

| Learning statement | Control | | mCURE | | cCURE | | Condition | |
| --- | --- | --- | --- | --- | --- | --- | --- | --- |
|  | *n* | Mean | *n* | Mean | *n* | Mean | F | *p* |
| Clarification of career path | 419 | 3.14 | 355 | 3.03 | 268 | 2.83 | 4.893 | 0.008 |
| Skill in interpretation of results | 454 | 3.70 | 378 | 3.65 | 285 | 3.78 | 1.448 | 0.235 |
| Tolerance for obstacles faced in the research  process | 449 | 3.62 | 375 | 3.70 | 283 | 3.78 | 2.237 | 0.107 |
| Readiness for more demanding research | 450 | 3.50 | 375 | 3.53 | 284 | 3.68 | 2.798 | 0.061 |
| Understanding how knowledge is constructed | 452 | 3.62 | 373 | 3.65 | 286 | 3.66 | 0.234 | 0.791 |
| Understanding of the research process in your field | 451 | 3.61 | 372 | 3.64 | 283 | 3.78 | 2.323 | 0.098 |
| Ability to integrate theory and practice | 450 | 3.65 | 375 | 3.70 | 284 | 3.73 | 0.480 | 0.619 |
| Understanding of how scientists work on real  problems | 453 | 3.77 | 375 | 3.81 | 285 | 3.85 | 0.528 | 0.590 |
| Understanding that scientific assertions require  supporting evidence | 452 | 3.80 | 376 | 3.84 | 282 | 3.88 | 0.533 | 0.587 |
| Ability to analyze data and other information | 454 | 3.88 | 376 | 3.88 | 287 | 3.93 | 0.256 | 0.774 |
| Understanding science | 453 | 3.88 | 376 | 3.79 | 286 | 3.87 | 0.953 | 0.386 |
| Learning ethical conduct in your field | 428 | 3.36 | 361 | 3.24 | 274 | 3.20 | 1.825 | 0.162 |
| Learning laboratory techniques | 455 | 4.13 | 375 | 4.05 | 286 | 4.15 | 1.039 | 0.354 |
| Ability to read and understand primary literature | 441 | 3.48 | 368 | 3.48 | 282 | 3.65 | 2.641 | 0.072 |
| Skill in how to give an effective oral presentation | 428 | 3.32 | 370 | 3.38 | 281 | 3.62 | 5.936 | 0.003 |
| Skill in science writing | 440 | 3.56 | 372 | 3.53 | 283 | 3.70 | 1.901 | 0.150 |
| Self-confidence | 444 | 3.43 | 374 | 3.43 | 279 | 3.51 | 0.531 | 0.588 |
| Understanding how scientists think | 447 | 3.60 | 377 | 3.57 | 284 | 3.74 | 2.275 | 0.103 |
| Learning to work independently | 442 | 3.48 | 369 | 3.45 | 280 | 3.64 | 2.680 | 0.069 |
| Becoming part of a learning community | 439 | 3.63 | 371 | 3.66 | 285 | 3.67 | 0.144 | 0.866 |
| Confidence in my ability to be a teacher of science | 416 | 3.18 | 352 | 3.18 | 267 | 3.29 | 0.672 | 0.511 |
