# Supplementary material for: Length of course-based undergraduate research experiences (CURE) impacts student learning and attitudinal outcomes: A study of the Malate dehydrogenase CUREs Community (MCC)
Source: PLoS One. 2023 Mar 9;18(3):e0282170. doi: 10.1371/journal.pone.0282170 (PMC9997910; doi:10.1371/journal.pone.0282170)
Supplement: S5 Table — Table A: TOSLS by CURE condition. Table B: TOSLS by URM status and interaction of status/condition. (DOCX) [file pone.0282170.s005.docx]

**S5 Table. TOSLS Data.** Table A:TOSLS by CURE condition. Table B: TOSLS by URM Status and Interaction of Status/Condition.

Table A:TOSLS by CURE condition.

| CURE Condition | | Pretest | | Posttest | | F | *p* |
| --- | --- | --- | --- | --- | --- | --- | --- |
|  | n | Mean | SE | Mean | SE |  |  |
| Control | 450 | 64.74 | 0.91 | 62.67 | 1.02 | F(1,1111) = 1.78 | 0.169 |
| mCURE | 373 | 65.16 | 0.98 | 63.54 | 1.18 |  |  |
| cCURE | 292 | 74.14 | 0.96 | 72.41 | 1.11 |  |  |

Table B: TOSLS by URM Status and Interaction of Status/Condition.

|  | CURE Condition | | Pretest | | Posttest | | URM Status | | Interaction of  Status/Condition | |
| --- | --- | --- | --- | --- | --- | --- | --- | --- | --- | --- |
|  |  | *n* | Mean | SE | Mean (SD) | SE | F | *p* | F | *p* |
| URM  students | Control | 107 | 60.78 | 1.71 | 58.28 | 1.91 | F(1,1029) = 1.45 | 0.229 | F(2,1029) = 1.53 | 0.216 |
|  | mCURE | 99 | 59.81 | 1.91 | 60.71 | 2.22 |  |  |  |  |
|  | cCURE | 48 | 67.78 | 2.81 | 64.29 | 3.11 |  |  |  |  |
|  | Overall | 254 | 61.73 | 1.32 | 60.39 | 1.32 |  |  |  |  |
| White/ Asian students | Control | 312 | 66.53 | 1.24 | 64.56 | 1.24 |  |  |  |  |
|  | mCURE | 244 | 68.40 | 1.16 | 66.20 | 1.46 |  |  |  |  |
|  | cCURE | 226 | 75.92 | 0.99 | 74.54 | 1.14 |  |  |  |  |
|  | Overall | 782 | 69.83 | 0.66 | 67.96 | 0.77 |  |  |  |  |
