# Supplementary material for: Length of course-based undergraduate research experiences (CURE) impacts student learning and attitudinal outcomes: A study of the Malate dehydrogenase CUREs Community (MCC)
Source: PLoS One. 2023 Mar 9;18(3):e0282170. doi: 10.1371/journal.pone.0282170 (PMC9997910; doi:10.1371/journal.pone.0282170)
Supplement: S6 Table — On the pretest, students responded to five items concerning their attitudes towards science using a scale from 1 = “Strongly disagree” to 7 = “Strongly agree.” On the posttest, students responded to the same five items using a scale from 1 = “Strongly disagree” to 5 = “Strongly agree”. (DOCX) [file pone.0282170.s006.docx]

**S6Table. Positive and Negative Attitudes.** On the pretest, students responded to five items concerning their attitudes towards science using a scale from 1 = “Strongly disagree” to 7 = “Strongly agree.” On the posttest, students responded to the same five items using a scale from 1 = “Strongly disagree” to 5 = “Strongly agree.” Please note that the scales pretest and posttest were not the same.

Table A: Overall Positive and Negative Attitudes

| Attitudes | CURE Condition | n | Pretest Mean* | Pretest SE | Posttest Mean* | Posttest SE |
| --- | --- | --- | --- | --- | --- | --- |
| Positive | Control | 455 | 5.94 | 0.03 | 4.19 | 0.02 |
|  | mCURE | 380 | 6.04 | 0.03 | 4.15 | 0.03 |
|  | cCURE | 287 | 6.08 | 0.04 | 4.30 | 0.03 |
|  | F(2,1116) = 7.78 | | *p* <0 .001 | η_p_^2^ = 0.014 | cCURE>mCURE, *p* < 0.001  mCURE<control, *p* = 0.018 | |
| Negative | Control | 456 | 3.06 | 0.04 | 2.55 | 0.04 |
|  | mCURE | 380 | 2.95 | 0.04 | 2.54 | 0.04 |
|  | cCURE | 287 | 2.75 | 0.05 | 2.53 | 0.04 |
|  | F(2,1116) = 2.14 | | *p* = 0.118 |  |  | |
| *Pretest and posttest were on different scales. Pretest from 1 to 7 and posttest from 1 to 5 | | | | | | |

Table B: URM Positive and Negative Attitudes

| Attitudes | CURE  Condition | URM Students | | | | | White/Asian Students | | | | |  |
| --- | --- | --- | --- | --- | --- | --- | --- | --- | --- | --- | --- | --- |
|  |  | *n* | x̄ Pre | SE | x̄ Post | SE | | *n* | x̄ Pre | SE | x̄ Post | SE |
| Positive | Control | 108 | 5.97 | 0.07 | 4.24 | 0.05 | | 316 | 5.96 | 0.04 | 4.17 | 0.03 |
|  | mCURE | 103 | 6.04 | 0.07 | 4.16 | 0.06 | | 248 | 6.08 | 0.07 | 4.15 | 0.06 |
|  | cCURE | 47 | 6.14 | 0.09 | 4.29 | 0.08 | | 222 | 6.10 | 0.09 | 4.31 | 0.08 |
|  | Overall | 258 | 6.03 | 0.04 | 4.21 | 0.03 | | 786 | 6.04 | 0.04 | 4.20 | 0.03 |
|  |  | | | | F | | | | *p* | | | |
|  | Effect of URM Status | | | | F(1,1037) = 0.21 | | | | 0.646 | | | |
|  | Interaction of Status/Condition | | | | F(2,1037) = 0.60 | | | | 0.548 | | | |
| Negative | CURE  Condition | URM Students | | | | | | White/Asian Students | | | |  |
|  |  | *n* | x̄ Pre | SE | x̄ Post | SE | | *n* | x̄ Pre | SE | x̄ Post | SE |
|  | Control | 108 | 3.04 | 0.08 | 2.61 | 0.07 | | 317 | 3.04 | 0.05 | 2.52 | 0.04 |
|  | mCURE | 103 | 2.99 | 0.07 | 2.51 | 0.07 | | 248 | 2.90 | 0.06 | 2.53 | 0.05 |
|  | cCURE | 47 | 2.88 | 0.16 | 2.41 | 0.12 | | 222 | 2.71 | 0.05 | 2.33 | 0.05 |
|  | Overall | 258 | 2.99 | 0.05 | 2.53 | 0.05 | | 787 | 2.90 | 0.03 | 2.47 | 0.03 |
|  |  | | |  | F | | | | *p* | | | |
|  | Effect of URM Status | | | | F(1,1038) = 0.03 | | | | 0.781 | | | |
|  | Interaction of Status/Condition | | | | F(2,1038) =1.02 | | | | 0.362 | | | |
