# Supplementary material for: Length of course-based undergraduate research experiences (CURE) impacts student learning and attitudinal outcomes: A study of the Malate dehydrogenase CUREs Community (MCC)
Source: PLoS One. 2023 Mar 9;18(3):e0282170. doi: 10.1371/journal.pone.0282170 (PMC9997910; doi:10.1371/journal.pone.0282170)
Supplement: S7 Table — On the pretest, students responded to eleven items concerning their attitudes towards science using a scale from 1 = “Strongly disagree” to 7 = “Strongly agree.” On the posttest, students responded to the same eleven items using a scale from 1 = “Strongly disagree” to 5 = “Strongly agree.” Table A: Beliefs about Science and Science Learning by CURE Condition. Table B: Beliefs about science and science learning by URM status and interaction of status/condition. (DOCX) [file pone.0282170.s007.docx]

**S7 Table. Beliefs About Science and Science Learning.** On the pretest, students responded to eleven items concerning their attitudes towards science using a scale from 1 = “Strongly disagree” to 7 = “Strongly agree.” On the posttest, students responded to the same eleven items using a scale from 1 = “Strongly disagree” to 5 = “Strongly agree.” Bonferroni correction for 11 tests results in *p* <0.005.

Table A: Beliefs About Science and Science Learning by CURE Condition.

| Statement | CURE  Condition | *n* | x̄ | SE Pre | x̄ | SE  Post | Condition | |
| --- | --- | --- | --- | --- | --- | --- | --- | --- |
|  |  |  | Pre* |  | Post* |  | F | *p* |
| You can rely on scientific results to be true and correct | Control | 453 | 4.65 | 0.07 | 3.41 | 0.05 | F(2,115) = 1.68 | 0.188 |
|  | mCURE | 379 | 4.67 | 0.08 | 3.32 | 0.05 |  |  |
|  | cCURE | 287 | 4.74 | 0.08 | 3.33 | 0.05 |  |  |
| When scientific results conflict with my personal experience, I follow my experience in making choices | Control | 423 | 3.92 | 0.08 | 3.25 | 0.05 | F(2,1048) = 1.20 | 0.302 |
|  | mCURE | 356 | 4.03 | 0.08 | 3.33 | 0.05 |  |  |
|  | cCURE | 273 | 3.64 | 0.10 | 3.14 | 0.16 |  |  |
| Students who do not major/concentrate in science should not have to take science courses | Control | 453 | 2.60 | 0.08 | 2.42 | 0.06 | F(2,1114) = 1.68 | 0.187 |
|  | mCURE | 378 | 2.48 | 0.08 | 2.40 | 0.06 |  |  |
|  | cCURE | 287 | 2.51 | 0.10 | 2.26 | 0.07 |  |  |
| When experts disagree on a science question, it’s because they don’t know all the facts yet | Control | 449 | 4.05 | 0.08 | 3.18 | 0.05 | F(2,1106) = 4.90 | 0.008 |
|  | mCURE | 374 | 3.94 | 0.09 | 2.95 | 0.05 |  |  |
|  | cCURE | 287 | 3.84 | 0.10 | 2.99 | 0.06 |  |  |
| Since nothing in science is known for certain, all theories are equally valid | Control | 448 | 3.33 | 0.08 | 2.65 | 0.05 | F(2,1104) = 2.74 | 0.065 |
|  | mCURE | 375 | 3.33 | 0.09 | 2.73 | 0.06 |  |  |
|  | cCURE | 285 | 3.09 | 0.08 | 2.47 | 0.06 |  |  |
| Real scientists don’t follow the scientific method in a straight line | Control | 444 | 4.18 | 0.08 | 3.32 | 0.05 | F(2,1089) = 0.46 | 0.629 |
|  | mCURE | 366 | 4.35 | 0.08 | 3.34 | 0.05 |  |  |
|  | cCURE | 283 | 4.23 | 0.06 | 3.38 | 0.06 |  |  |
| Only scientific experts are qualified to make judgements on scientific issues | Control | 452 | 3.08 | 0.08 | 2.64 | 0.05 | F(2,1107) = 0.71 | 0.490 |
|  | mCURE | 374 | 2.85 | 0.08 | 2.59 | 0.06 |  |  |
|  | cCURE | 285 | 3.12 | 0.10 | 2.58 | 0.06 |  |  |

| Scientists know what the results of their experiments will be before they start | Control | 454 | 2.14 | 0.06 | 2.13 | 0.05 | F(2,1112) = 4.66 | 0.010 |
| --- | --- | --- | --- | --- | --- | --- | --- | --- |
|  | mCURE | 375 | 2.05 | 0.06 | 2.07 | 0.05 |  |  |
|  | cCURE | 287 | 1.97 | 0.07 | 1.87 | 0.06 |  |  |
| The main job of the instructor is to structure the work so that we can learn it ourselves | Control | 454 | 4.42 | 0.08 | 3.38 | 0.05 | F(2,1114) = 0.89 | 0.409 |
|  | mCURE | 378 | 4.37 | 0.09 | 3.41 | 0.05 |  |  |
|  | cCURE | 286 | 4.47 | 0.10 | 3.49 | 0.06 |  |  |
| Scientists play with statistics to support their own ideas | Control | 443 | 3.81 | 0.09 | 2.88 | 0.05 | F(2,1085) = 0.78 | 0.458 |
|  | mCURE | 363 | 3.94 | 0.09 | 2.94 | 0.06 |  |  |
|  | cCURE | 283 | 3.53 | 0.10 | 2.73 | 0.07 |  |  |
| Lab experiments are used to confirm information studied in science class | Control | 452 | 5.52 | 0.06 | 3.86 | 0.04 | F(2,1108) = 3.44 | 0.032 |
|  | mCURE | 377 | 5.55 | 0.07 | 3.84 | 0.05 |  |  |
|  | cCURE | 283 | 5.24 | 0.08 | 3.64 | 0.06 |  |  |

*Pretest and Posttest are on different scales. Pretest is 1 to 7 and Posttest is 1 to 5.

Table B: Beliefs About Science and Science Learning by URM Status and Interaction of Status/Condition.

| Statement | CURE  Type | URM Students | | | | | White/Asian Students | | | | |
| --- | --- | --- | --- | --- | --- | --- | --- | --- | --- | --- | --- |
|  |  | *n* | x̄ Pre* | SE | x̄  Post* | SE | *n* | x̄ Pre* | SE | x̄ Post* | SE |
| You can rely on scientific results to be true and correct | Control | 107 | 4.74 | 0.16 | 3.47 | 0.10 | 315 | 4.62 | 0.09 | 3.38 | 0.05 |
|  | mCURE | 102 | 4.40 | 0.14 | 3.20 | 0.11 | 248 | 4.79 | 0.10 | 3.31 | 0.06 |
|  | cCURE | 47 | 4.91 | 0.21 | 3.34 | 0.15 | 222 | 4.77 | 0.09 | 3.35 | 0.06 |
|  | Overall | 256 | 4.64 | 0.10 | 3.34 | 0.07 | 785 | 4.71 | 0.05 | 3.35 | 0.03 |
|  |  | | | | | F | | | *p* | | |
|  | Effect of URM Status | | | | | F(1.1034) < 0.01 | | | 0.991 | | |
|  | Interaction of Status/Condition | | | | | F(2,1034) = 0.24 | | | 0.785 | | |
| When scientific results conflict with my personal experience, I follow my experience in making choices | CURE  Type | URM Students | | | | | White/Asian Students | | | | |
|  |  | *n* | x̄ Pre | SE | x̄ Post | SE | *n* | x̄ Pre | SE | x̄ Post | SE |
|  | Control | 99 | 3.76 | 0.17 | 3.32 | 0.10 | 294 | 3.94 | 0.09 | 3.20 | 0.06 |
|  | mCURE | 97 | 3.93 | 0.15 | 3.38 | 0.10 | 232 | 4.06 | 0.10 | 3.28 | 0.07 |
|  | cCURE | 45 | 3.87 | 0.25 | 3.36 | 0.15 | 211 | 3.63 | 0.11 | 3.09 | 0.07 |
|  | Overall | 241 | 3.85 | 0.10 | 3.35 | 0.06 | 737 | 3.89 | 0.06 | 3.20 | 0.04 |
|  |  | | | | | F | | | *p* | | |
|  | Effect of URM Status | | | | | F(1,971) = 4.66 | | | 0.031 | | |
|  | Interaction of Status/Condition | | | | | F(2,971) = 0.12 | | | 0.889 | | |
| Statement | CURE  Type | URM Students | | | | | White/Asian Students | | | | |
|  |  | *n* | x̄ Pre* | SE | x̄  Post* | SE | *n* | x̄ Pre* | SE | x̄ Post* | SE |
| Students who do not major/concentrate in science should not have to take science courses | Control | 107 | 2.61 | 0.16 | 2.55 | 0.12 | 315 | 2.60 | 0.09 | 2.37 | 0.06 |
|  | mCURE | 101 | 2.62 | 0.17 | 2.40 | 0.12 | 248 | 2.45 | 0.09 | 2.37 | 0.08 |
|  | cCURE | 47 | 2.51 | 0.29 | 2.36 | 0.17 | 222 | 2.52 | 0.11 | 2.23 | 0.08 |
|  | Overall | 255 | 2.60 | 0.11 | 2.45 | 0.08 | 785 | 2.53 | 0.06 | 2.33 | 0.04 |
|  |  | | | | | F | | | *p* | | |
|  | Effect of URM Status | | | | | F(1,1033) = 1.28 | | | 0.258 | | |
|  | Interaction of Status/Condition | | | | | F(2,1033) = 0.67 | | | 0.514 | | |
| When experts disagree on a science question, it’s because they don’t know all the facts yet | CURE  Type | URM Students | | | | | White/Asian Students | | | | |
|  |  | *n* | x̄ Pre* | SE | x̄ Post* | SE | *n* | x̄ Pre* | SE | x̄ Post* | SE |
|  | Control | 107 | 4.22 | 0.17 | 3.34 | 0.09 | 312 | 4.00 | 0.10 | 3.13 | 0.06 |
|  | mCURE | 101 | 3.88 | 0.17 | 2.89 | 0.11 | 244 | 4.02 | 0.11 | 2.98 | 0.06 |
|  | cCURE | 47 | 4.04 | 0.26 | 3.26 | 0.15 | 222 | 3.84 | 0.11 | 2.96 | 0.07 |
|  | Overall | 255 | 4.05 | 0.11 | 3.15 | 0.07 | 778 | 3.96 | 0.06 | 3.03 | 0.04 |
|  |  | | | | | F | | | *p* | | |
|  | Effect of URM Status | | | | | F(1,1026) = 2.59 | | | 0.108 | | |
|  | Interaction of Status/Condition | | | | | F(2,1026) = 1.69 | | | 0.185 | | |

| Since nothing in science is known for certain, all theories are equally valid | CURE  Type | URM Students | | | | | White/Asian Students | | | | |
| --- | --- | --- | --- | --- | --- | --- | --- | --- | --- | --- | --- |
|  |  | *n* | x̄ Pre | SE | x̄ Post | SE | *n* | x̄ Pre | SE | x̄ Post | SE |
|  | Control | 104 | 3.36 | 0.17 | 2.74 | 0.16 | 313 | 3.30 | 0.09 | 3.30 | 0.06 |
|  | mCURE | 101 | 3.42 | 0.16 | 2.76 | 0.12 | 245 | 3.27 | 0.17 | 3.27 | 0.07 |
|  | cCURE | 47 | 3.15 | 0.27 | 2.69 | 0.17 | 220 | 3.07 | 0.11 | 3.07 | 0.07 |
|  | Overall | 252 | 3.34 | 0.11 | 2.69 | 0.07 | 778 | 3.23 | 0.06 | 3.23 | 0.04 |
|  |  | | | | | F | | | *p* | | |
|  | Effect of URM Status | | | | | F(1.1023) = 0.43 | | | 0.512 | | |
|  | Interaction of Status/Condition | | | | | F(2.1023) = 0.46 | | | 0.635 | | |
| Statement | CURE  Type | URM Students | | | | | White/Asian Students | | | | |
|  |  | *n* | x̄ Pre* | SE | x̄  Post* | SE | *n* | x̄ Pre* | SE | x̄ Post* | SE |
| Real scientists don’t follow the scientific method in a straight line | Control | 102 | 4.00 | 0.17 | 3.29 | 0.11 | 311 | 4.24 | 0.09 | 3.33 | 0.06 |
|  | mCURE | 100 | 4.16 | 0.17 | 3.26 | 0.10 | 237 | 4.45 | 0.10 | 3.33 | 0.06 |
|  | cCURE | 47 | 4.57 | 0.26 | 3.38 | 0.15 | 219 | 4.18 | 0.10 | 3.41 | 0.07 |
|  | Overall | 249 | 4.17 | 0.11 | 3.30 | 0.07 | 767 | 4.29 | 0.06 | 3.35 | 0.04 |
|  |  | | | | | F | | | *p* | | |
|  | Effect of URM Status | | | | | F(1,1009) = 0.22 | | | 0.639 | | |
|  | Interaction of Status/Condition | | | | | F(2,1009) = 0.38 | | | 0.683 | | |

| Only scientific experts are qualified to make judgements on scientific issues | CURE  Type | URM Students | | | | | White/Asian Students | | | | |
| --- | --- | --- | --- | --- | --- | --- | --- | --- | --- | --- | --- |
|  |  | *n* | x̄ Pre* | SE | x̄ Post* | SE | *n* | x̄ Pre* | SE | x̄ Post* | SE |
|  | Control | 107 | 3.17 | 0.17 | 2.68 | 0.11 | 314 | 3.04 | 0.09 | 2.60 | 0.06 |
|  | mCURE | 102 | 2.53 | 0.13 | 2.37 | 0.10 | 243 | 2.95 | 0.10 | 2.65 | 0.07 |
|  | cCURE | 47 | 3.38 | 0.29 | 2.47 | 0.17 | 221 | 3.07 | 0.10 | 2.57 | 0.07 |
|  | Overall | 256 | 2.95 | 0.11 | 2.52 | 0.07 | 778 | 3.02 | 0.06 | 2.61 | 0.04 |
|  |  | | | | | F | | | *p* | | |
|  | Effect of URM Status | | | | | F(1,1027) = 1.73 | | | 0.189 | | |
|  | Interaction of Status/Condition | | | | | F(2,1027) = 1.14 | | | 0.322 | | |

| Scientists know what the results of their experiments will be before they start | CURE  Type | URM Students | | | | | White/Asian Students | | | | |
| --- | --- | --- | --- | --- | --- | --- | --- | --- | --- | --- | --- |
|  |  | *n* | x̄ Pre | SE | x̄ Post | SE | *n* | x̄ Pre | SE | x̄ Post | SE |
|  | Control | 108 | 2.06 | 0.12 | 2.13 | 0.09 | 315 | 2.13 | 0.07 | 2.11 | 0.06 |
|  | mCURE | 102 | 2.09 | 0.12 | 2.04 | 0.10 | 245 | 2.01 | 0.07 | 2.05 | 0.07 |
|  | cCURE | 47 | 1.91 | 0.18 | 1.94 | 0.16 | 222 | 1.98 | 0.07 | 1.86 | 0.06 |
|  | Overall | 257 | 2.05 | 0.08 | 2.06 | 0.06 | 782 | 2.05 | 0.04 | 2.02 | 0.04 |
|  |  | | | | | F | | | *p* | | |
|  | Effect of URM Status | | | | | F(1.1032) = 0.21 | | | 0.647 | | |
|  | Interaction of Status/Condition | | | | | F(2,1032) = 0.24 | | | 0.791 | | |
| Statement | CURE  Type | URM Students | | | | | White/Asian Students | | | | |
|  |  | *n* | x̄ Pre* | SE | x̄  Post* | SE | *n* | x̄ Pre* | SE | x̄ Post* | SE |
| The main job of the instructor is to structure the work so that we can learn it ourselves | Control | 108 | 4.09 | 0.18 | 3.31 | 0.10 | 315 | 4.51 | 0.09 | 3.38 | 0.09 |
|  | mCURE | 103 | 4.31 | 0.18 | 3.39 | 0.11 | 246 | 4.39 | 0.11 | 3.40 | 0.06 |
|  | cCURE | 47 | 4.53 | 0.26 | 3.49 | 0.15 | 221 | 4.46 | 0.07 | 3.46 | 0.07 |
|  | Overall | 258 | 4.26 | 0.11 | 3.37 | 0.07 | 782 | 4.46 | 0.06 | 3.41 | 0.04 |
|  |  | | | | | F | | | *p* | | |
|  | Effect of URM Status | | | | | F(1,1033) = 0.02 | | | 0.890 | | |
|  | Interaction of Status/Condition | | | | | F(2,1033) < 0.01 | | | 0.997 | | |
| Scientists play with statistics to support their own ideas | CURE  Type | URM Students | | | | | White/Asian Students | | | | |
|  |  | *n* | x̄ Pre* | SE | x̄ Post* | SE | *n* | x̄ Pre* | SE | x̄ Post* | SE |
|  | Control | 104 | 3.98 | 0.18 | 3.05 | 0.12 | 308 | 3.69 | 0.10 | 2.79 | 0.06 |
|  | mCURE | 100 | 4.33 | 0.17 | 3.02 | 0.12 | 234 | 3.72 | 0.11 | 2.84 | 0.07 |
|  | cCURE | 47 | 3.81 | 0.27 | 2.98 | 0.17 | 219 | 3.47 | 0.11 | 2.67 | 0.08 |
|  | Overall | 251 | 4.09 | 0.11 | 3.02 | 0.08 | 761 | 3.64 | 0.06 | 2.77 | 0.04 |
|  |  | | | | | F | | | *p* | | |
|  | Effect of URM Status | | | | | F(1,1005) = 2.90 | | | 0.089 | | |
|  | Interaction of Status/Condition | | | | | F(2,1005) = 0.70 | | | 0.497 | | |

| Statement | CURE  Type | URM Students | | | | | White/Asian Students | | | | |
| --- | --- | --- | --- | --- | --- | --- | --- | --- | --- | --- | --- |
|  |  | *n* | x̄ Pre* | SE | x̄  Post* | SE | *n* | x̄ Pre* | SE | x̄ Post* | SE |
| Lab experiments are used to confirm information studied in science class | Control | 105 | 5.69 | 0.14 | 3.96 | 0.08 | 316 | 5.47 | 0.07 | 3.81 | 0.05 |
|  | mCURE | 103 | 5.87 | 0.18 | 3.96 | 0.09 | 245 | 5.44 | 0.08 | 3.79 | 0.06 |
|  | cCURE | 47 | 5.57 | 0.19 | 3.87 | 0.12 | 219 | 5.21 | 0.09 | 3.61 | 0.06 |
|  | Overall | 255 | 5.74 | 0.08 | 3.95 | 0.05 | 780 | 5.39 | 0.05 | 3.74 | 0.03 |
|  |  | | | | | F | | | *p* | | |
|  | Effect of URM Status | | | | | F(1,1028) = 4.06 | | | 0.044 | | |
|  | Interaction of Status/Condition | | | | | F(2,1028) = 0.19 | | | 0.827 | | |

*Pretest and Posttest are on different scales. Pretest is 1 to 7 and Posttest is 1 to 5.
