# Supplementary material for: Length of course-based undergraduate research experiences (CURE) impacts student learning and attitudinal outcomes: A study of the Malate dehydrogenase CUREs Community (MCC)
Source: PLoS One. 2023 Mar 9;18(3):e0282170. doi: 10.1371/journal.pone.0282170 (PMC9997910; doi:10.1371/journal.pone.0282170)
Supplement: S8 Table — Table A: STEM support, career interest, importance by CURE condition. Table B: STEM support, career interest, importance by CURE post benefits by URM status and interaction of status/condition. (DOCX) [file pone.0282170.s008.docx]

**S8 Table. STEM Support, Career Interest, Importance.** Table A: STEM Support, Career Interest, Importance by CURE condition. Table B: STEM Support, Career Interest, Importance by CURE Post Benefits by URM Status and Interaction of Status/Condition.

**Table A: STEM Support, Career Interest, Importance by CURE condition**.

| Posttest Items | CURE  Condition | *n* | Mean | SE | F | η_p_^2^ | Condition  *p-*values & Post-hoc test *p-*values |
| --- | --- | --- | --- | --- | --- | --- | --- |
| STEM support | Control | 455 | 3.90 | 0.04 | F(2,1119) =5.13 | 0.009 | 0.006  cCURE>mCURE, *p*=0.006  cCURE>control, *p*=0.030 |
|  | mCURE | 371 | 3.86 | 0.05 |  |  |  |
|  | cCURE | 296 | 4.06 | 0.04 |  |  |  |
| STEM career interest | Control | 455 | 3.98 | 0.04 | F(2,1119) =8.98 | 0.016 | <0.001  cCURE>mCURE, *p*=0.001  cCURE>control, *p*=0.013 |
|  | mCURE | 371 | 3.88 | 0.05 |  |  |  |
|  | cCURE | 296 | 4.15 | 0.04 |  |  |  |
| STEM importance | Control | 455 | 4.21 | 0.03 | F(2,1119) =7.11 | 0.013 | 0.001  cCURE>mCURE, *p*=0.005  cCURE>control, *p*=0.001 |
|  | mCURE | 371 | 4.22 | 0.04 |  |  |  |
|  | cCURE | 296 | 4.38 | 0.03 |  |  |  |

**Table B: STEM Support, Career Interest, Importance by CURE Post Benefits by URM Status and Interaction of Status/Condition.**

| Posttest Items | CURE  Condition | URM  Students | | | White/Asian  Students | | | URM Status | | | Interaction of  Status/Condition | |
| --- | --- | --- | --- | --- | --- | --- | --- | --- | --- | --- | --- | --- |
|  |  | *n* | Mean | SE | *n* | Mean | SE | F | η_p_^2^ | *p* | F | *p* |
| STEM support | Control | 108 | 4.02 | 0.06 | 314 | 3.85 | 0.05 | F(1,1034) =1.47 |  | 0.226 | F(2,1034) =0.93 | 0.396 |
|  | mCURE | 101 | 3.87 | 0.10 | 239 | 3.87 | 0.06 |  |  |  |  |  |
|  | cCURE | 48 | 4.10 | 0.09 | 230 | 4.05 | 0.05 |  |  |  |  |  |
|  | Overall | 257 | 3.98 | 0.05 | 783 | 3.92 | 0.03 |  |  |  |  |  |
| STEM career interest | Control | 108 | 4.14 | 0.06 | 314 | 3.91 | 0.05 | F(1,1034) =4.24 | 0.004 | 0.040  URM>non | F(2,1034) =0.82 | 0.439 |
|  | mCURE | 101 | 3.94 | 0.09 | 239 | 3.88 | 0.06 |  |  |  |  |  |
|  | cCURE | 48 | 4.25 | 0.10 | 230 | 4.14 | 0.05 |  |  |  |  |  |
|  | Overall | 257 | 4.08 | 0.05 | 783 | 3.97 | 0.03 |  |  |  |  |  |
| STEM importance | Control | 108 | 4.34 | 0.05 | 314 | 4.15 | 0.04 | F(1,1034)  =5.37 | 0.005 | 0.021  URM>non | F(2,1034)  =0.93 | 0.397 |
|  | mCURE | 101 | 4.28 | 0.06 | 239 | 4.22 | 0.05 |  |  |  |  |  |
|  | cCURE | 48 | 4.46 | 0.07 | 230 | 4.37 | 0.04 |  |  |  |  |  |
|  | Overall | 257 | 4.34 | 0.04 | 783 | 4.24 | 0.02 |  |  |  |  |  |
