# Supplementary material for: Length of course-based undergraduate research experiences (CURE) impacts student learning and attitudinal outcomes: A study of the Malate dehydrogenase CUREs Community (MCC)
Source: PLoS One. 2023 Mar 9;18(3):e0282170. doi: 10.1371/journal.pone.0282170 (PMC9997910; doi:10.1371/journal.pone.0282170)
Supplement: S9 Table — Data collected from institutional data. Not all institutions responded to request for this data. Table A: GPA. One year post-CURE cumulative GPA from students still enrolled (excludes graduates). Table B: Retention. One year post-CURE enrollment or graduation status. Students who graduated or were still enrolled were included. (DOCX) [file pone.0282170.s009.docx]

S9 Table. GPA and Retention. Data collected from institutional data. Not all institutions responded to request for this data. Table A: GPA. One year post-CURE cumulative GPA from students still enrolled (excludes graduates). Table B: Retention. One year post-CURE enrollment or graduation status. Students who graduated or were still enrolled were included.

Table A. GPA

| CURE Condition | n | Mean | SE | F | *p* |
| --- | --- | --- | --- | --- | --- |
| Control | 183 | 3.45 | 0.03 | F(2,279) = 0.35 | 0.709 |
| mCURE | 87 | 3.40 | 0.05 |  |  |
| cCURE | 12 | 3.41 | 0.16 |  |  |

Table B. Retention

| CURE Condition | n | Retained | Not Retained | χ^2^, *df* = 2 | *p* |
| --- | --- | --- | --- | --- | --- |
| Control | 361 | 88.6% | 11.4% | χ^2^ = 5.85 | 0.054 |
| mCURE | 180 | 95.0% | 5.0% |  |  |
| cCURE | 47 | 91.5% | 8.5% |  |  |
