# Supplementary material for: Length of course-based undergraduate research experiences (CURE) impacts student learning and attitudinal outcomes: A study of the Malate dehydrogenase CUREs Community (MCC)
Source: PLoS One. 2023 Mar 9;18(3):e0282170. doi: 10.1371/journal.pone.0282170 (PMC9997910; doi:10.1371/journal.pone.0282170)
Supplement: S10 Table — Table A. Overall Evaluation. Table B. Overall Evaluation by URM status and interaction of status/condition. For the four statements, Bonferroni correction indicated that p < 0.013 was significant. (DOCX) [file pone.0282170.s010.docx]

**S10 Table. Overall Evaluation.** Table A. Overall Evaluation. Table B. Overall Evaluation by URM status and interaction of status/condition. For the four statements, Bonferroni correction indicated that *p* < 0.013 was significant.

**Table A. Overall Evaluation**

| Item  This course… | CURE Condition | *n* | Mean | SE | F | *p* | η_p_^2^ |
| --- | --- | --- | --- | --- | --- | --- | --- |
| was a good way of learning about the subject matter. | Control | 458 | 5.79 | 0.06 | F(2,1124) = 3.79 | 0.023 |  |
|  | mCURE | 381 | 5.66 | 0.08 |  |  |  |
|  | cCURE | 288 | 5.96 | 0.07 |  |  |  |
| was a good way of learning about the process of scientific research | Control | 458 | 5.78 | 0.06 | F(2,1123) = 7.65 | <0.001 | 0.013  cCURE> control, <0.001  cCURE>mCURE, 0.005 |
|  | mCURE | 380 | 5.82 | 0.07 |  |  |  |
|  | cCURE | 288 | 6.15 | 0.07 |  |  |  |
| had a positive effect on my interest in science | Control | 455 | 5.68 | 0.06 | F(2,1119) = 2.87 | 0.057 |  |
|  | mCURE | 381 | 5.46 | 0.09 |  |  |  |
|  | cCURE | 286 | 5.71 | 0.09 |  |  |  |
| I was able to ask question in this class and get helpful responses. | Control | 441 | 6.05 | 0.06 | F(2,1060) = 3.00 | 0.050 |  |
|  | mCURE | 353 | 5.95 | 0.07 |  |  |  |
|  | cCURE | 269 | 6.20 | 0.08 |  |  |  |

**Table B. Overall Evaluation by URM Status and Interaction of Status/Condition**

| Item  This course… | CURE Condition | Student status | *n* | Mean | SE | URM status | | | Interaction status/condition | |
| --- | --- | --- | --- | --- | --- | --- | --- | --- | --- | --- |
|  |  |  |  |  |  | F | *p* | η_p_^2^ | F | *p* |
| was a good way of learning about the subject matter. | Control | URM | 108 | 6.13 | 0.10 | F(1,1041) = 15.73 | <0.001 | 0.015 | F(2,1041) = 0.09 | 0.916 |
|  |  | White/Asian | 318 | 5.68 | 0.08 |  |  |  |  |  |
|  | mCURE | URM | 103 | 5.97 | 0.13 |  |  |  |  |  |
|  |  | White/Asian | 248 | 5.52 | 0.10 |  |  |  |  |  |
|  | cCURE | URM | 47 | 6.26 | 0.16 |  |  |  |  |  |
|  |  | White/Asian | 223 | 5.91 | 0.09 |  |  |  |  |  |
|  | Overall | URM | 258 | 6.09 | 0.07 |  |  |  |  |  |
|  |  | White/Asian | 789 | 5.70 | 0.05 |  |  |  |  |  |
| was a good way of learning about the process of scientific research | Control | URM | 108 | 6.05 | 0.11 | F(1,1040) = 18.23 | <0.001 | 0.017 | F(2,1040) = 0.15 | 0.859 |
|  |  | White/Asian | 318 | 5.69 | 0.07 |  |  |  |  |  |
|  | mCURE | URM | 102 | 6.16 | 0.13 |  |  |  |  |  |
|  |  | White/Asian | 248 | 5.68 | 0.09 |  |  |  |  |  |
|  | cCURE | URM | 67 | 6.53 | 0.11 |  |  |  |  |  |
|  |  | White/Asian | 223 | 6.09 | 0.09 |  |  |  |  |  |
|  | Overall | URM | 257 | 6.18 | 0.07 |  |  |  |  |  |
|  |  | White/Asian | 789 | 5.80 | 0.05 |  |  |  |  |  |
| had a positive effect on my interest in science | Control | URM | 106 | 6.00 | 0.11 | F(1,1037) = 19.15 | <0.001 | 0.018 | F(2,1037) = 0.10 | 0.908 |
|  |  | White/Asian | 317 | 5.56 | 0.09 |  |  |  |  |  |
|  | mCURE | URM | 103 | 5.81 | 0.16 |  |  |  |  |  |
|  |  | White/Asian | 248 | 5.28 | 0.11 |  |  |  |  |  |
|  | cCURE | URM | 47 | 6.17 | 0.18 |  |  |  |  |  |
|  |  | White/Asian | 222 | 5.60 | 0.10 |  |  |  |  |  |
|  | Overall | URM | 256 | 5.95 | 0.09 |  |  |  |  |  |
|  |  | White/Asian | 787 | 5.48 | 0.06 |  |  |  |  |  |
| I was able to ask question in this class and get helpful responses. | Control | URM | 105 | 6.24 | 0.10 | F(1,983) = 6.67 | 0.010 | 0.007 | F(2,983) = 0.03 | 0.969 |
|  |  | White/Asian | 305 | 6.00 | 0.08 |  |  |  |  |  |
|  | mCURE | URM | 98 | 6.15 | 0.12 |  |  |  |  |  |
|  |  | White/Asian | 228 | 5.86 | 0.09 |  |  |  |  |  |
|  | cCURE | URM | 44 | 6.41 | 0.14 |  |  |  |  |  |
|  |  | White/Asian | 209 | 6.16 | 0.09 |  |  |  |  |  |
|  | Overall | URM | 247 | 6.23 | 0.07 |  |  |  |  |  |
|  |  | White/Asian | 742 | 6.00 | 0.05 |  |  |  |  |  |
