# Supplementary material for: Length of course-based undergraduate research experiences (CURE) impacts student learning and attitudinal outcomes: A study of the Malate dehydrogenase CUREs Community (MCC)
Source: PLoS One. 2023 Mar 9;18(3):e0282170. doi: 10.1371/journal.pone.0282170 (PMC9997910; doi:10.1371/journal.pone.0282170)
Supplement: S11 Table — Table A: EDAT by CURE condition. Table B: EDAT by URM Status and Interaction of Status/Condition. (DOCX) [file pone.0282170.s011.docx]

**S11 Table. EDAT Data.** Table A: EDAT by CURE condition. Table B: EDAT by URM Status and Interaction of Status/Condition.

Table A: EDAT by CURE condition.

| CURE Condition | | Pretest | | Posttest | | F | *p* | η_p_^2^ |
| --- | --- | --- | --- | --- | --- | --- | --- | --- |
|  | n | Mean | SE | Mean | SE |  |  |  |
| Control | 397 | 4.05 | 0.09 | 4.59 | 0.09 | F(2,1040) = 3.572  cCURE>Control | <0.05 | 0.007 |
| mCURE | 389 | 4.27 | 0.09 | 4,89 | 0.09 |  |  |  |
| cCURE | 258 | 4.97 | 0.11 | 5.24 | 0.09 |  |  |  |

Table B: EDAT by URM Status and Interaction of Status/Condition.

|  | CURE Condition | | Pretest | | Posttest | | URM Status | | Interaction of  Status/Condition | |
| --- | --- | --- | --- | --- | --- | --- | --- | --- | --- | --- |
|  |  | *n* | Mean | SE | Mean | SE | F | *p* | F | *p* |
| URM  students | Control | 83 | 3.76 | 0.25 | 4.75 | 0.2 | F(1.964) = 0.30 | 0.585 | F(2,964) = 1.32 | 0.268 |
|  | mCURE | 122 | 3.87 | 0.17 | 4.69 | 0.15 |  |  |  |  |
|  | cCURE | 33 | 4.50 | 0.29 | 4.08 | 0.29 |  |  |  |  |
|  | Overall | 238 | 3.92 | 0.13 | 4.77 | 0.10 |  |  |  |  |
| White/  Asian students | Control | 288 | 4.20 | 0.11 | 4.59 | 0.10 |  |  |  |  |
|  | mCURE | 232 | 4.53 | 0.12 | 5.03 | 0.12 |  |  |  |  |
|  | cCURE | 213 | 5.10 | 0.12 | 5.26 | 0.12 |  |  |  |  |
|  | Overall | 733 | 4.57 | 0.07 | 4.92 | 0.06 |  |  |  |  |
