# Supplementary material for: Length of course-based undergraduate research experiences (CURE) impacts student learning and attitudinal outcomes: A study of the Malate dehydrogenase CUREs Community (MCC)
Source: PLoS One. 2023 Mar 9;18(3):e0282170. doi: 10.1371/journal.pone.0282170 (PMC9997910; doi:10.1371/journal.pone.0282170)
Supplement: S12 Table — Table A: Plans to conduct research by CURE condition. Table B: Plans to conduct research by URM status and interaction of status/condition. At the end of the semester, students were asked if they planned to conduct research in the future (yes or no). (DOCX) [file pone.0282170.s012.docx]

**S12 Table. Plans to Conduct Research**. Table A: Plans to conduct research by CURE condition. Table B: Plans to conduct research by URM status and interaction of status/condition. At the end of the semester, students were asked if they planned to conduct research in the future (yes or no).

Table A. Plans to conduct research by CURE condition.

| CURE Condition | *n* | Yes | No | *χ*^2^, *df* = 2 | *p* |
| --- | --- | --- | --- | --- | --- |
| Control | 455 | 55.2% | 44.8% | *χ*^2^ = 20.70 | <0.001 |
| mCURE | 371 | 53.6% | 46.4% |  |  |
| cCURE | 296 | 69.6% | 30.4% |  |  |

Table B. Plans to conduct research by URM status and interaction of status/condition.

|  | CURE Condition | Students | N | Yes | No | *χ*^2^, *df* = 1 | *p* |
| --- | --- | --- | --- | --- | --- | --- | --- |
| URM Status | All conditions | URM | 257 | 59.1% | 40.8% | *χ*^2^ = 0.132 | 0.716 |
|  |  | White/Asian | 783 | 57.9% | 42.1% |  |  |
| Interaction of status/ condition | Control | URM | 108 | 54.6% | 45.4% | *χ*^2^ = 0.02 | 0.888 |
|  |  | White/Asian | 314 | 55.4% | 44.6% |  |  |
|  | mCURE | URM | 101 | 63.4% | 36.6% | *χ*^2^ = 6.27 | 0.012 |
|  |  | White/Asian | 239 | 48.5% | 51.5% |  |  |
|  | cCURE | URM | 48 | 60.4% | 39.6% | *χ*^2^ = 2.03 | 0.154 |
|  |  | White/Asian | 230 | 70.9% | 29.1% |  |  |
